# Supplementary figures and images for: miRNA normalization enables joint analysis of several datasets to increase sensitivity and to reveal novel miRNAs differentially expressed in breast cancer (part 1 of 2)
Source: PLoS Comput Biol. 2021 Feb 10;17(2):e1008608. doi: 10.1371/journal.pcbi.1008608 (PMC7901788; doi:10.1371/journal.pcbi.1008608)

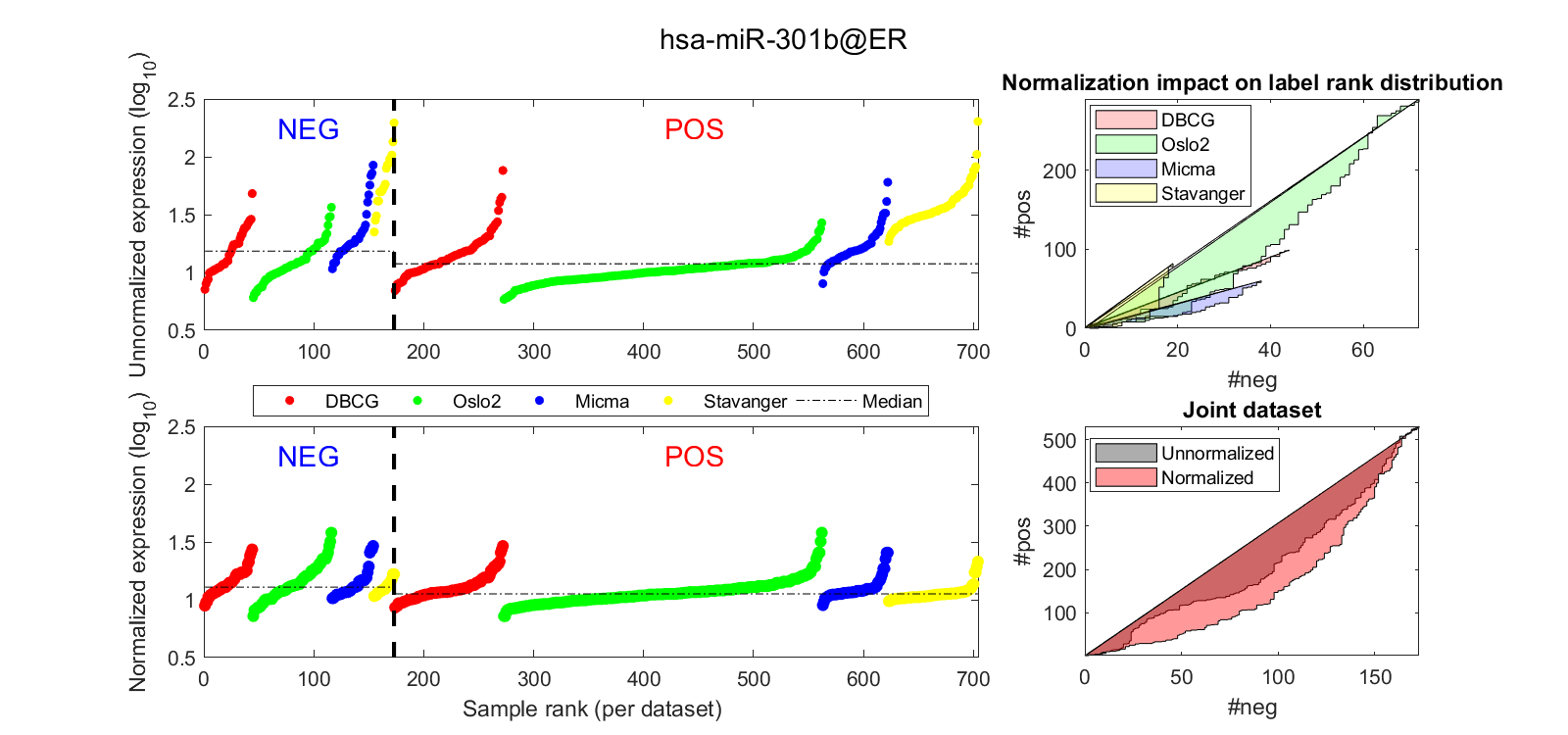

Supplement: S1 Data — Contains additional figures per miRNA pertaining to the analysis presented in Fig 2. (ZIP) [file pcbi.1008608.s001.zip › hsa-miR-301b.png]

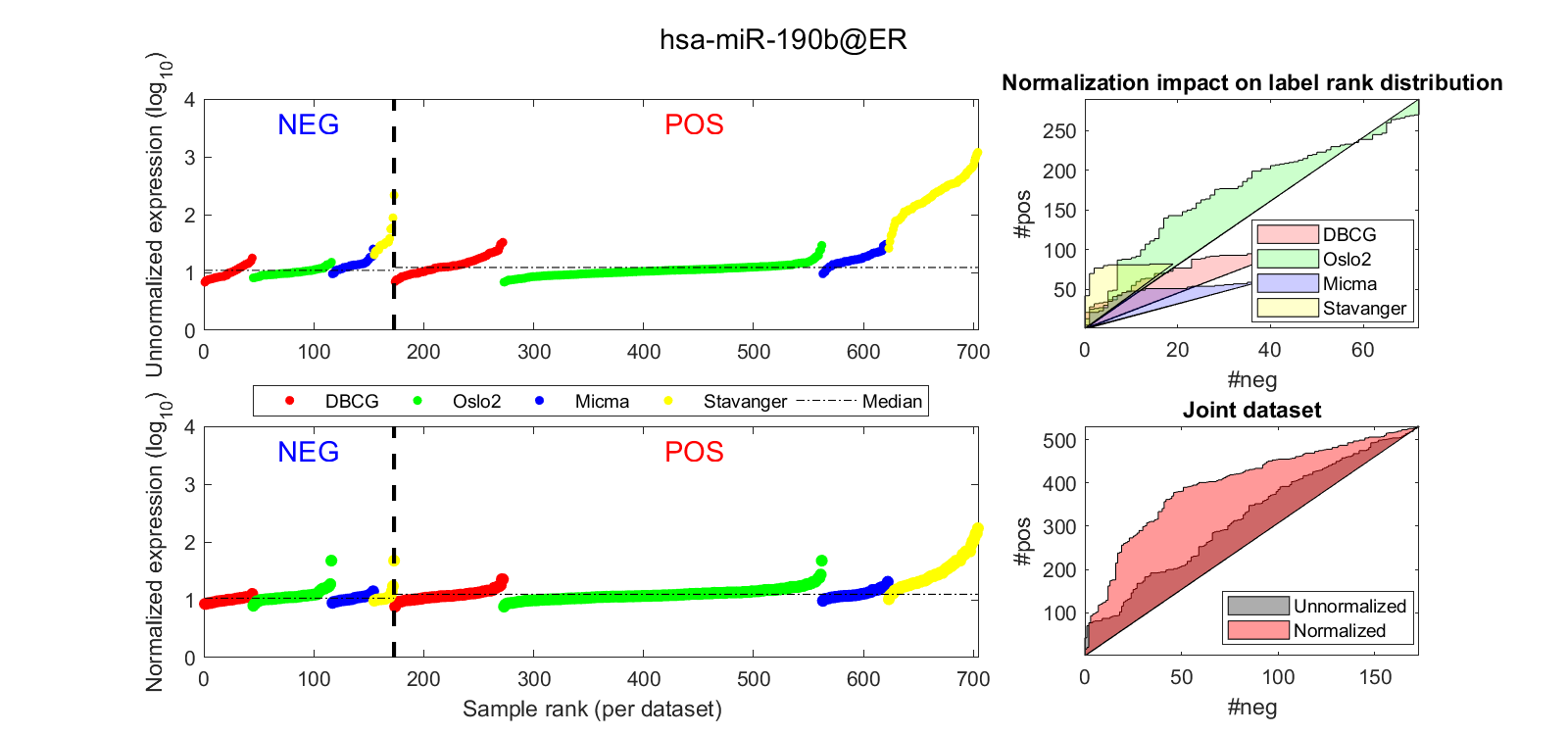

Supplement: S1 Data — Contains additional figures per miRNA pertaining to the analysis presented in Fig 2. (ZIP) [file pcbi.1008608.s001.zip › hsa-miR-190b.png]

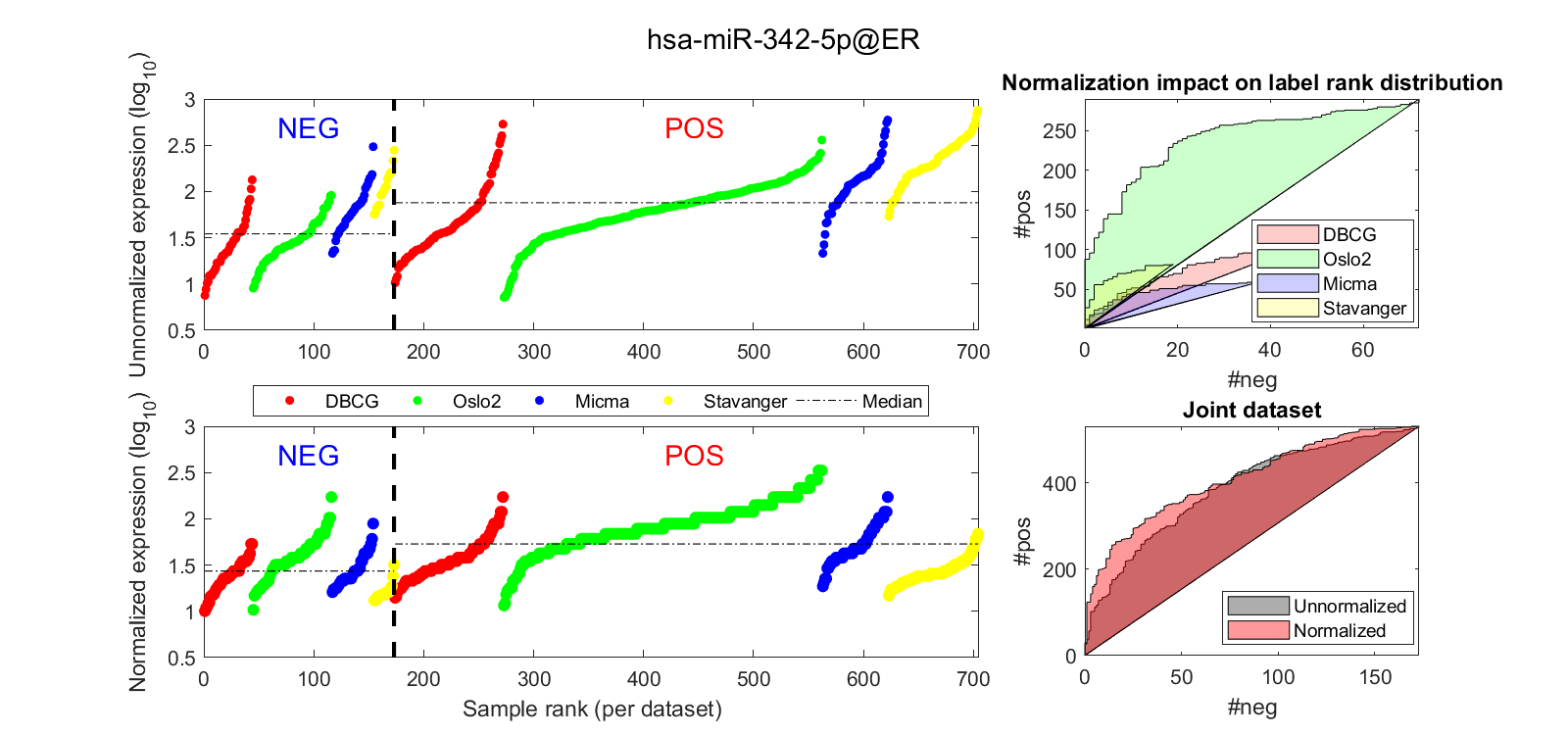

Supplement: S1 Data — Contains additional figures per miRNA pertaining to the analysis presented in Fig 2. (ZIP) [file pcbi.1008608.s001.zip › hsa-miR-342-5p.png]

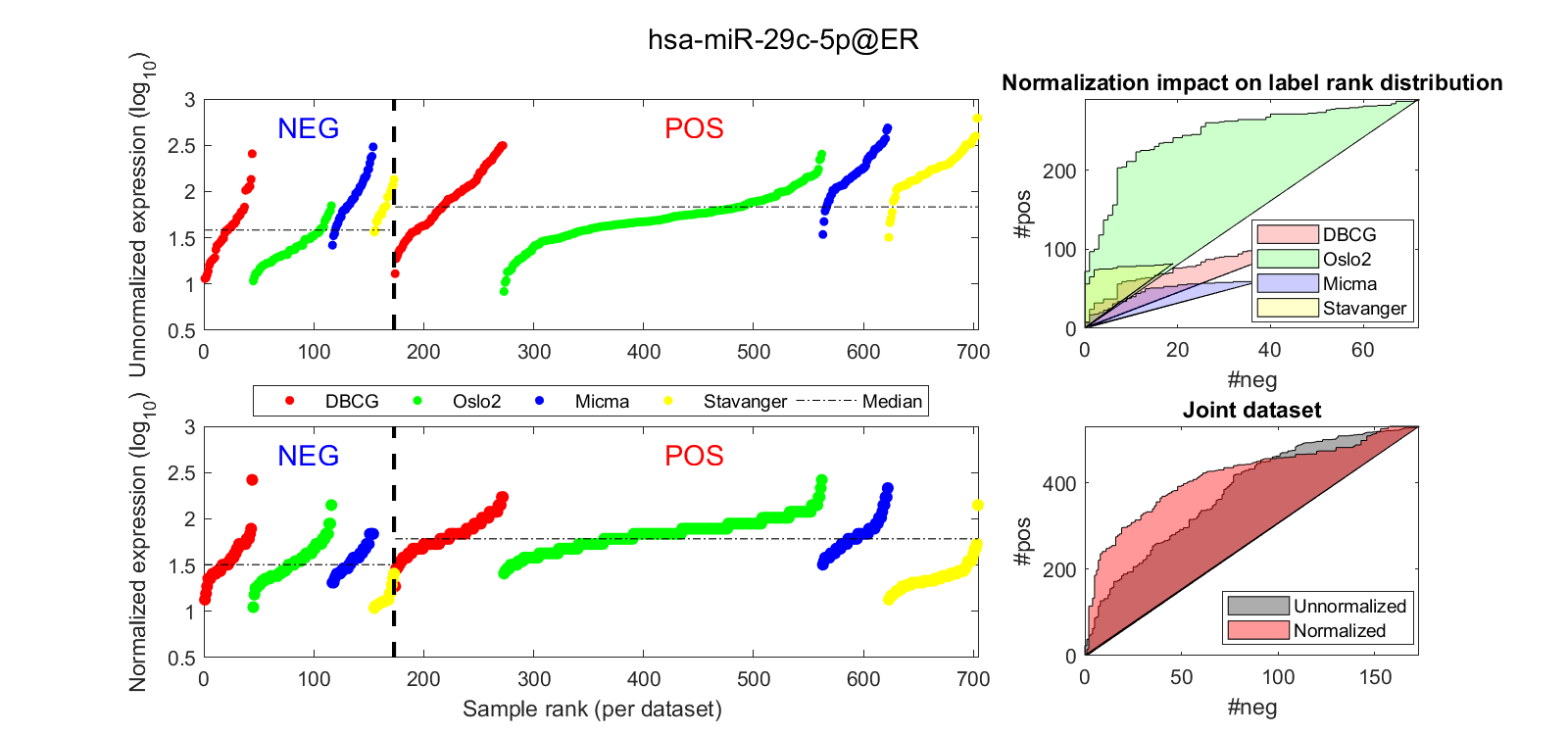

Supplement: S1 Data — Contains additional figures per miRNA pertaining to the analysis presented in Fig 2. (ZIP) [file pcbi.1008608.s001.zip › hsa-miR-29c-5p.png]

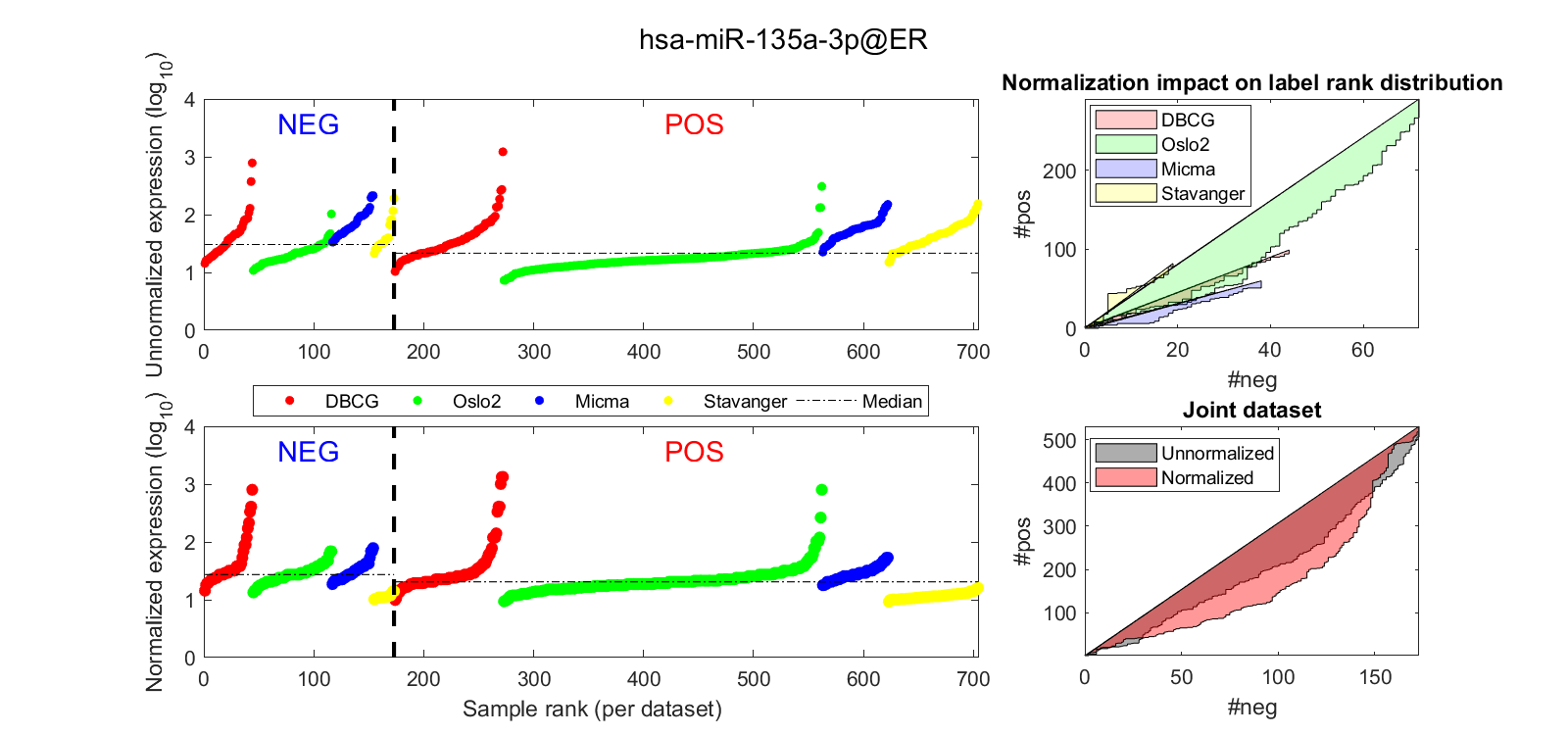

Supplement: S1 Data — Contains additional figures per miRNA pertaining to the analysis presented in Fig 2. (ZIP) [file pcbi.1008608.s001.zip › hsa-miR-135a-3p.png]

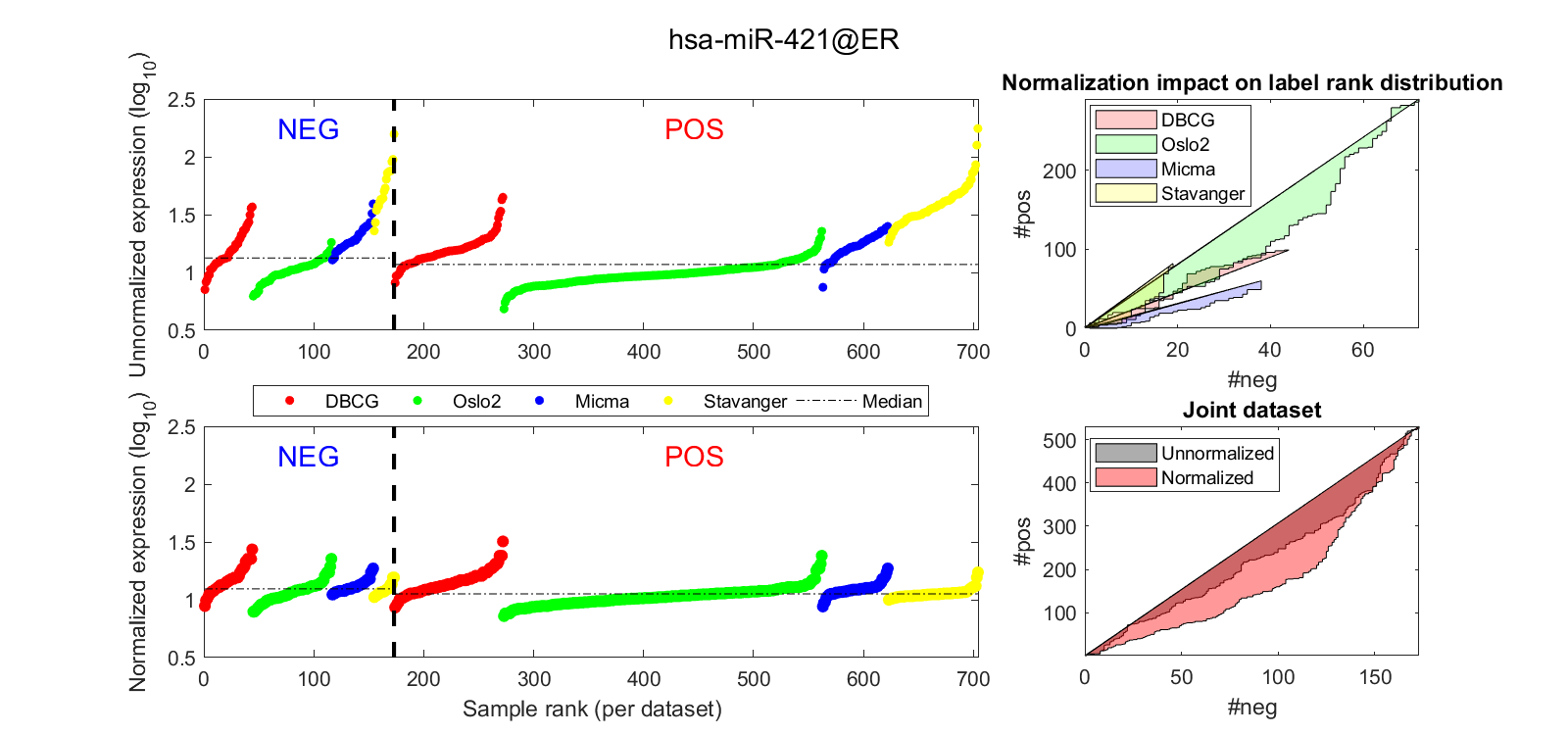

Supplement: S1 Data — Contains additional figures per miRNA pertaining to the analysis presented in Fig 2. (ZIP) [file pcbi.1008608.s001.zip › hsa-miR-421.png]

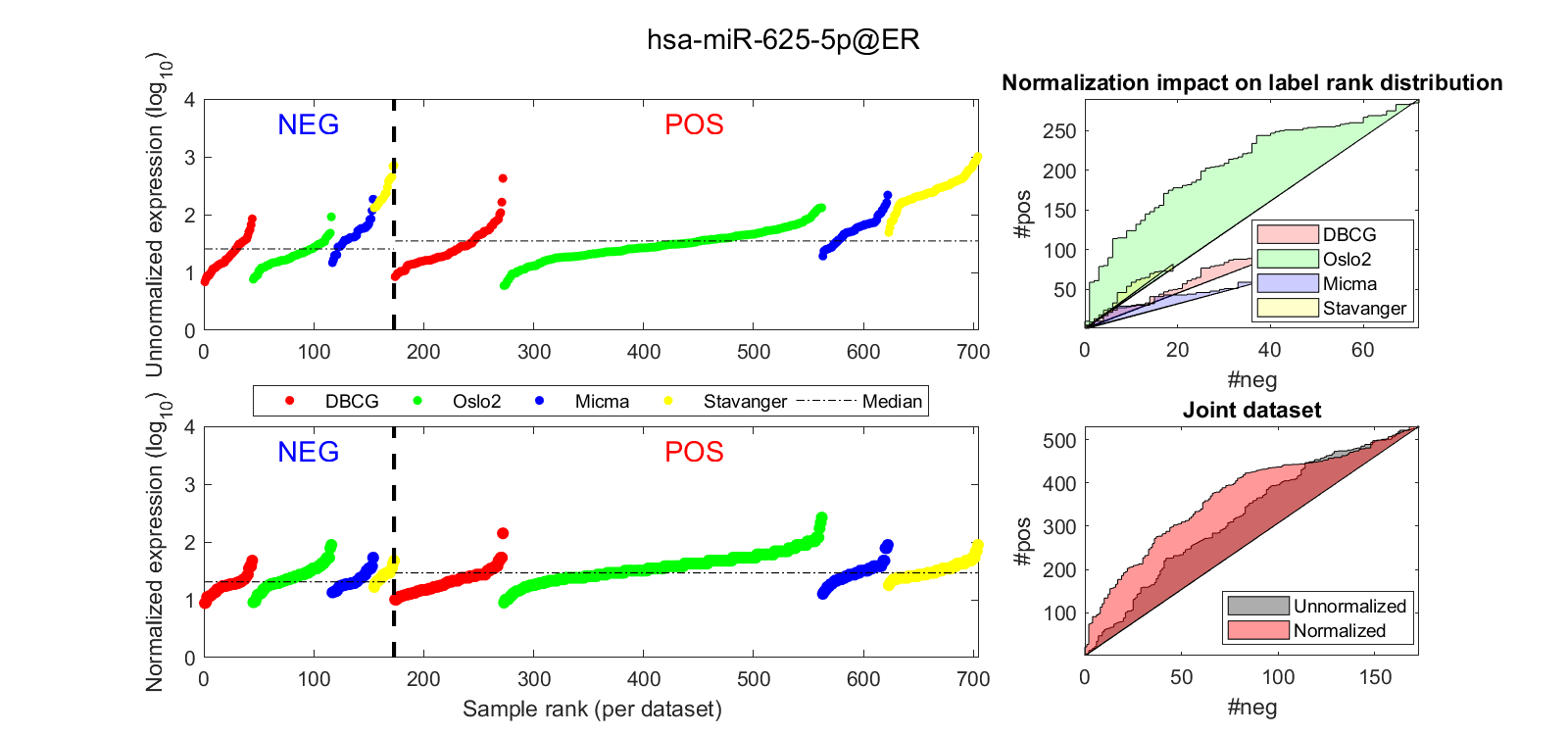

Supplement: S1 Data — Contains additional figures per miRNA pertaining to the analysis presented in Fig 2. (ZIP) [file pcbi.1008608.s001.zip › hsa-miR-625-5p.png]

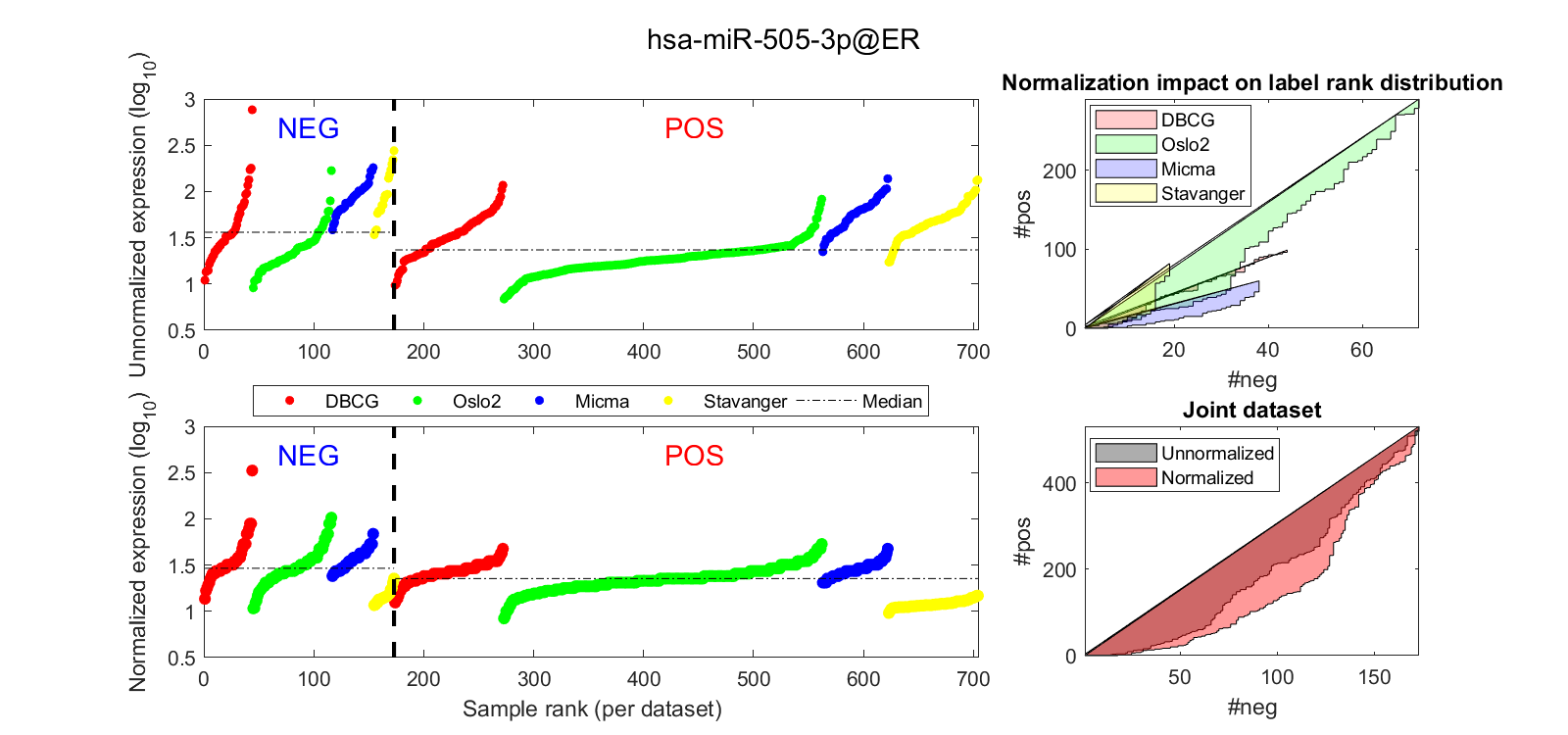

Supplement: S1 Data — Contains additional figures per miRNA pertaining to the analysis presented in Fig 2. (ZIP) [file pcbi.1008608.s001.zip › hsa-miR-505-3p.png]

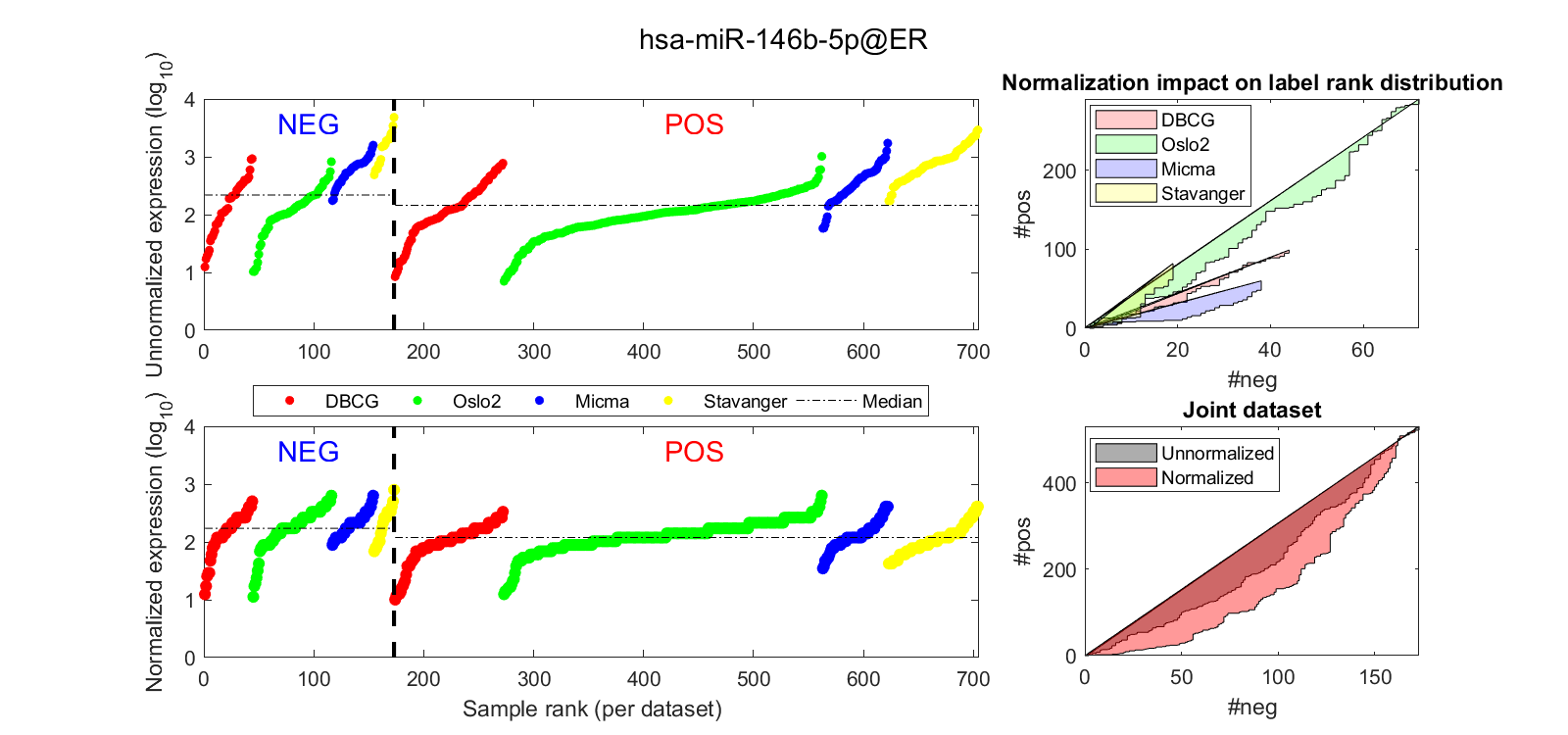

Supplement: S1 Data — Contains additional figures per miRNA pertaining to the analysis presented in Fig 2. (ZIP) [file pcbi.1008608.s001.zip › hsa-miR-146b-5p.png]

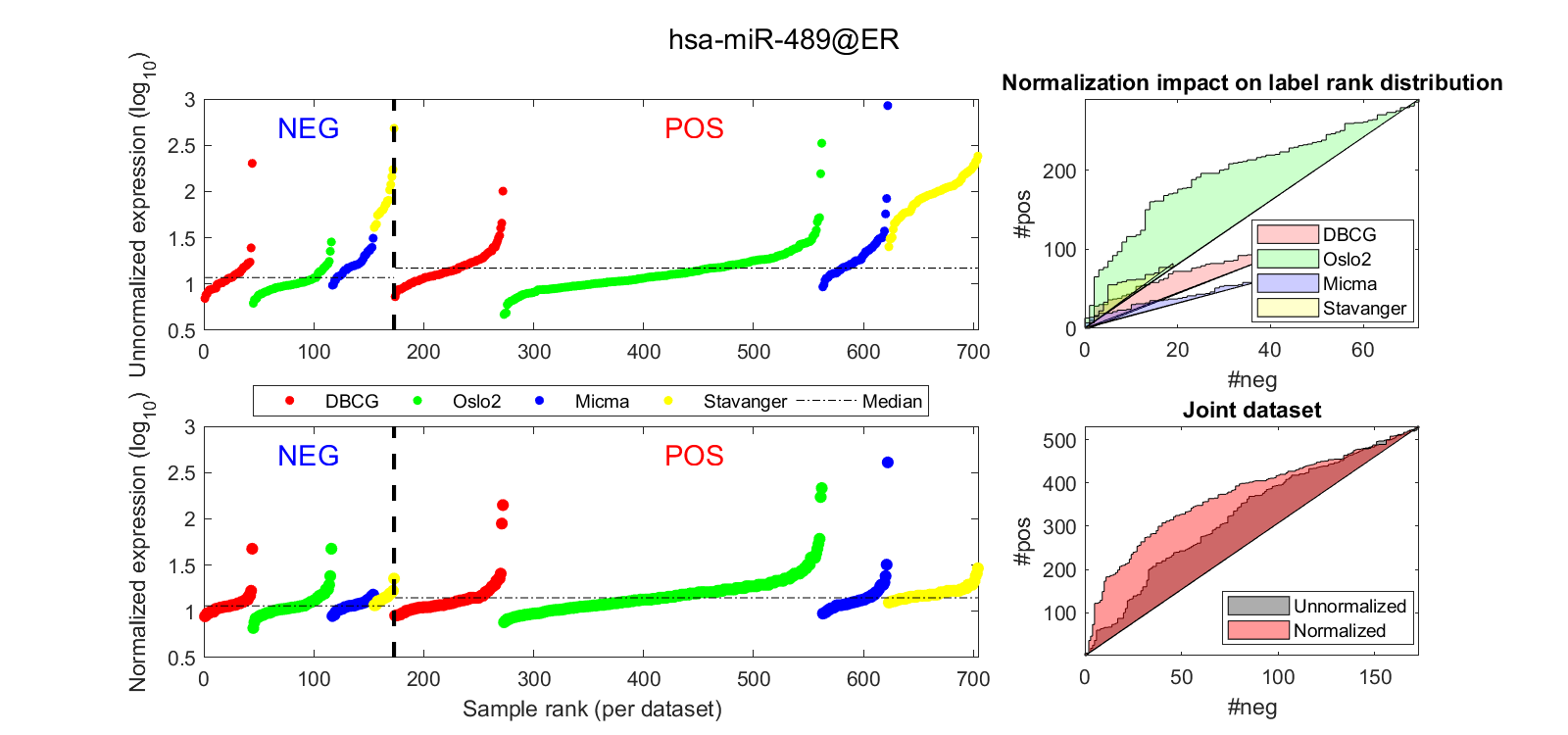

Supplement: S1 Data — Contains additional figures per miRNA pertaining to the analysis presented in Fig 2. (ZIP) [file pcbi.1008608.s001.zip › hsa-miR-489.png]

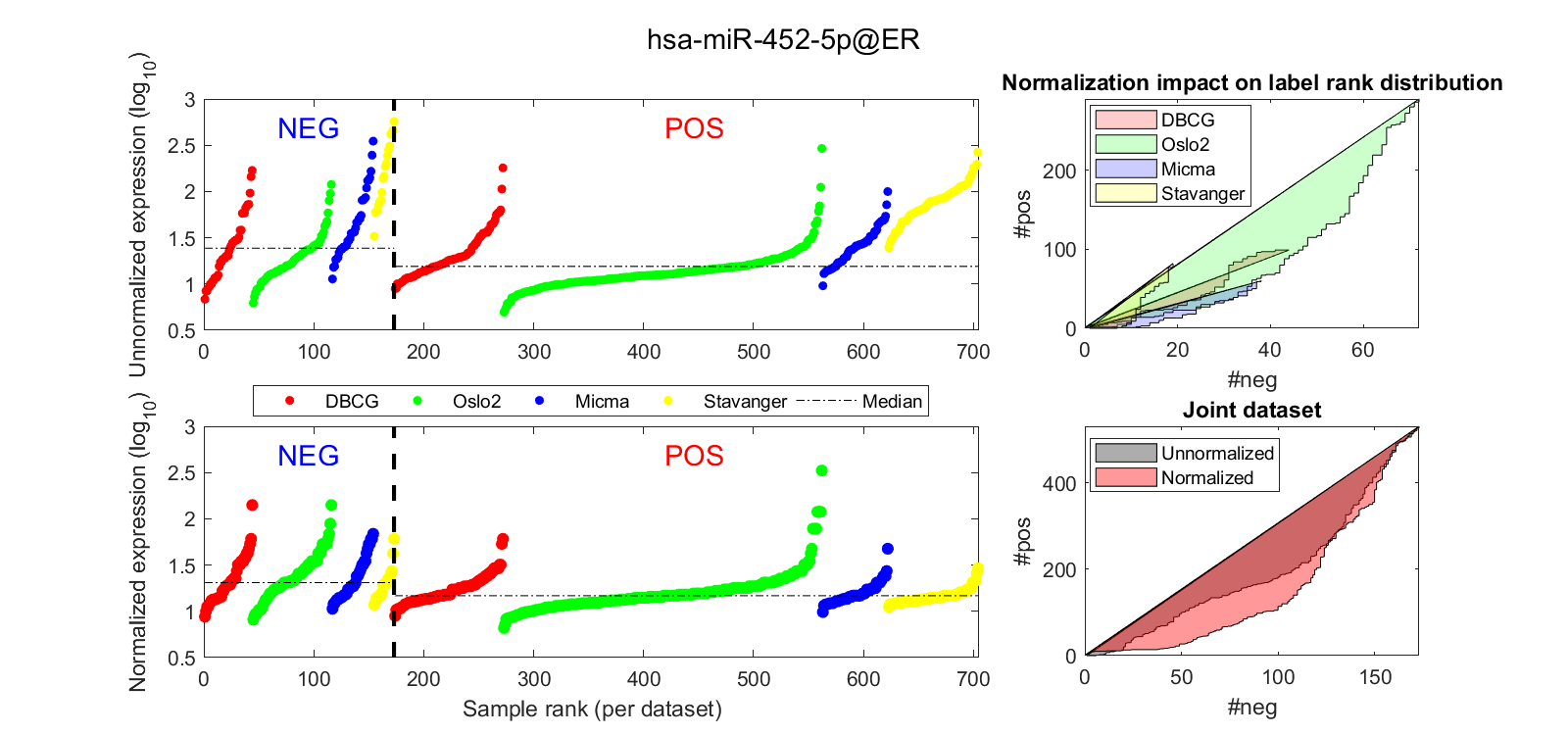

Supplement: S1 Data — Contains additional figures per miRNA pertaining to the analysis presented in Fig 2. (ZIP) [file pcbi.1008608.s001.zip › hsa-miR-452-5p.png]

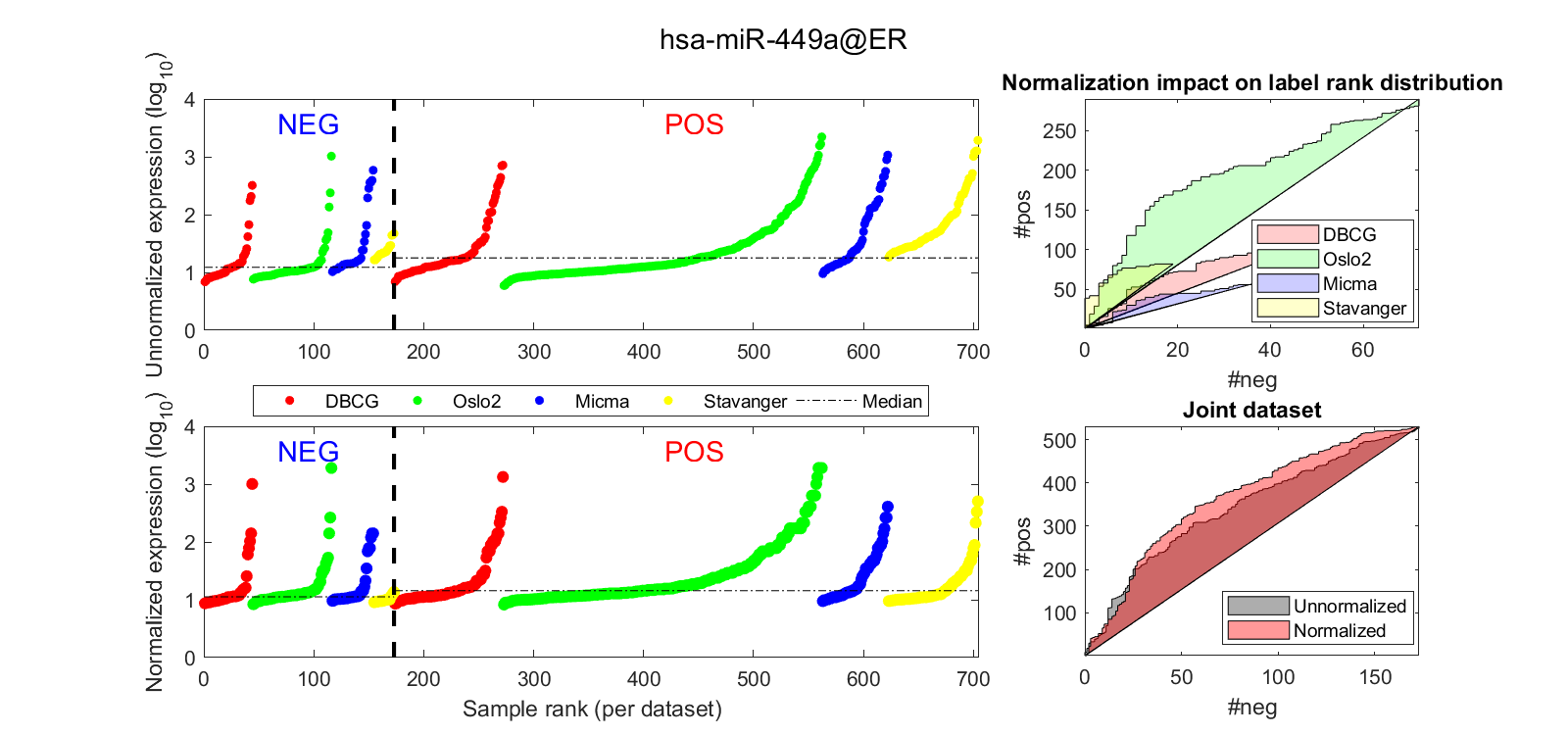

Supplement: S1 Data — Contains additional figures per miRNA pertaining to the analysis presented in Fig 2. (ZIP) [file pcbi.1008608.s001.zip › hsa-miR-449a.png]

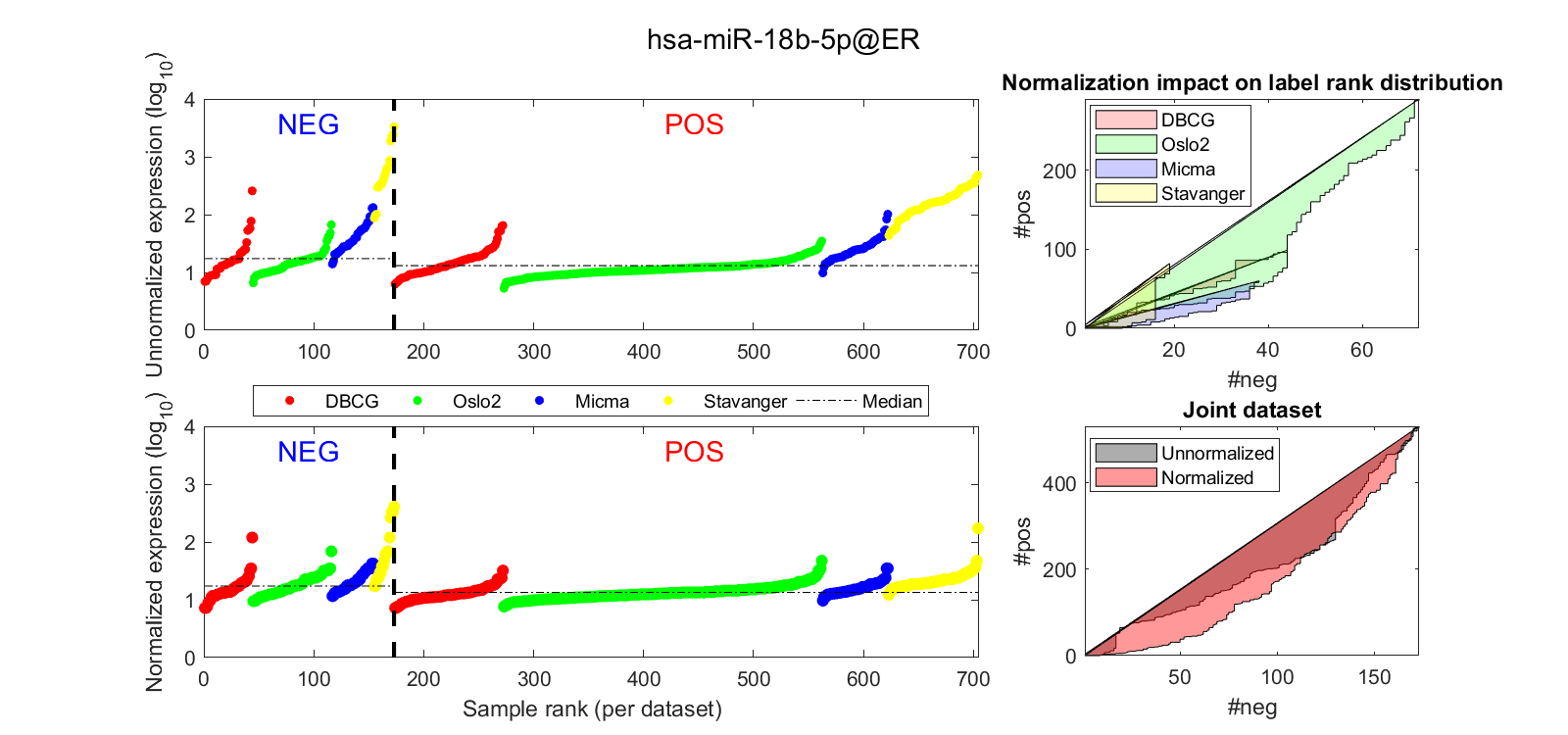

Supplement: S1 Data — Contains additional figures per miRNA pertaining to the analysis presented in Fig 2. (ZIP) [file pcbi.1008608.s001.zip › hsa-miR-18b-5p.png]

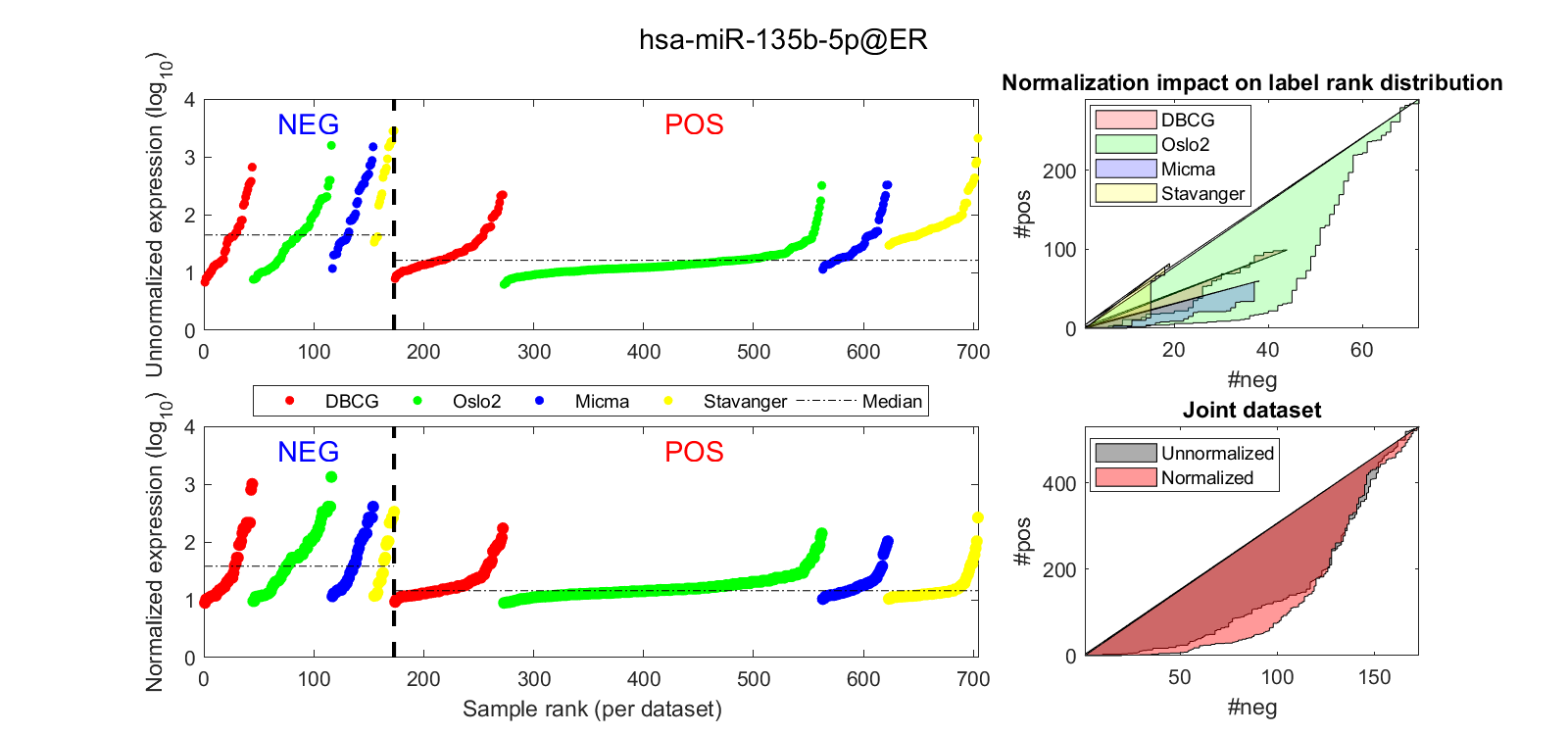

Supplement: S1 Data — Contains additional figures per miRNA pertaining to the analysis presented in Fig 2. (ZIP) [file pcbi.1008608.s001.zip › hsa-miR-135b-5p.png]

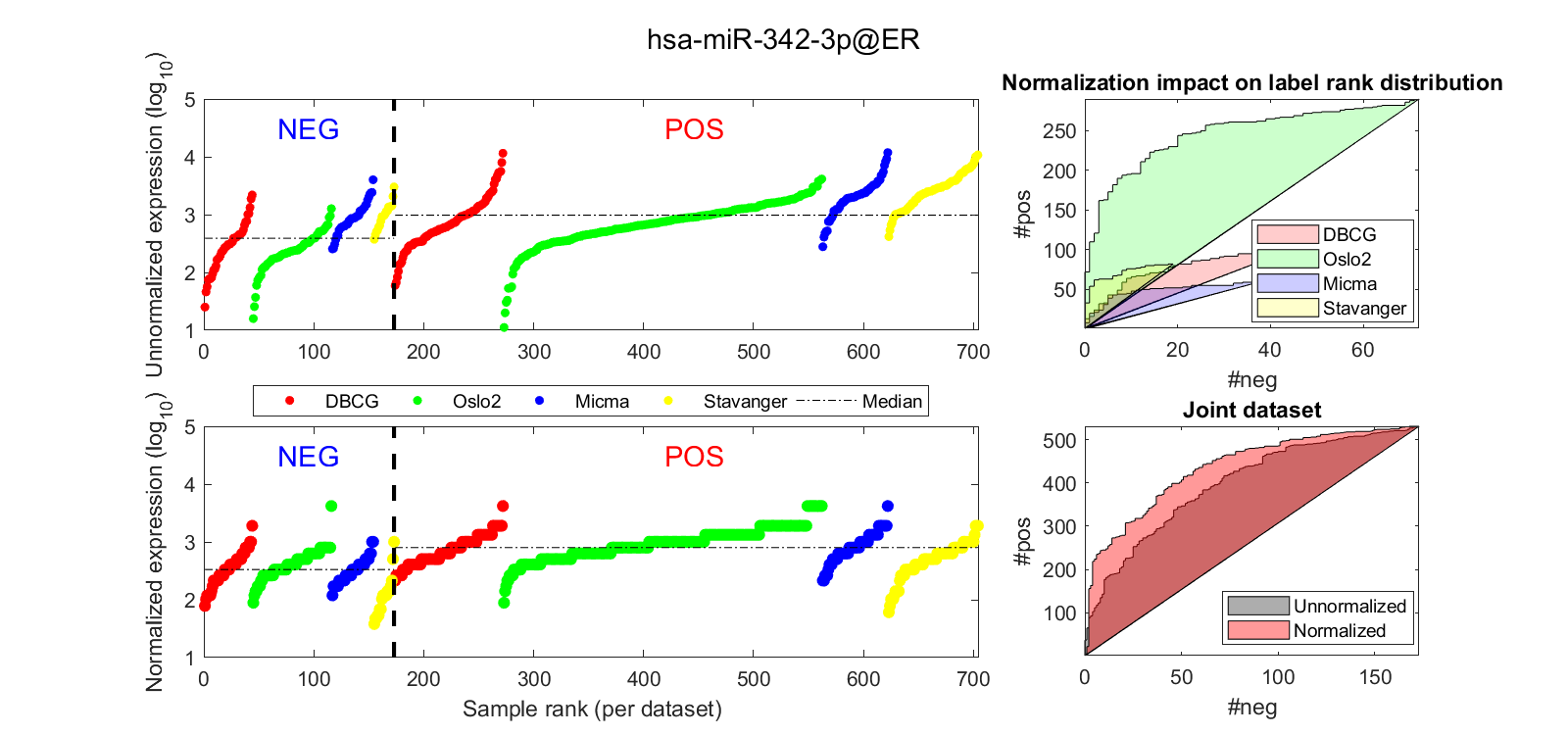

Supplement: S1 Data — Contains additional figures per miRNA pertaining to the analysis presented in Fig 2. (ZIP) [file pcbi.1008608.s001.zip › hsa-miR-342-3p.png]

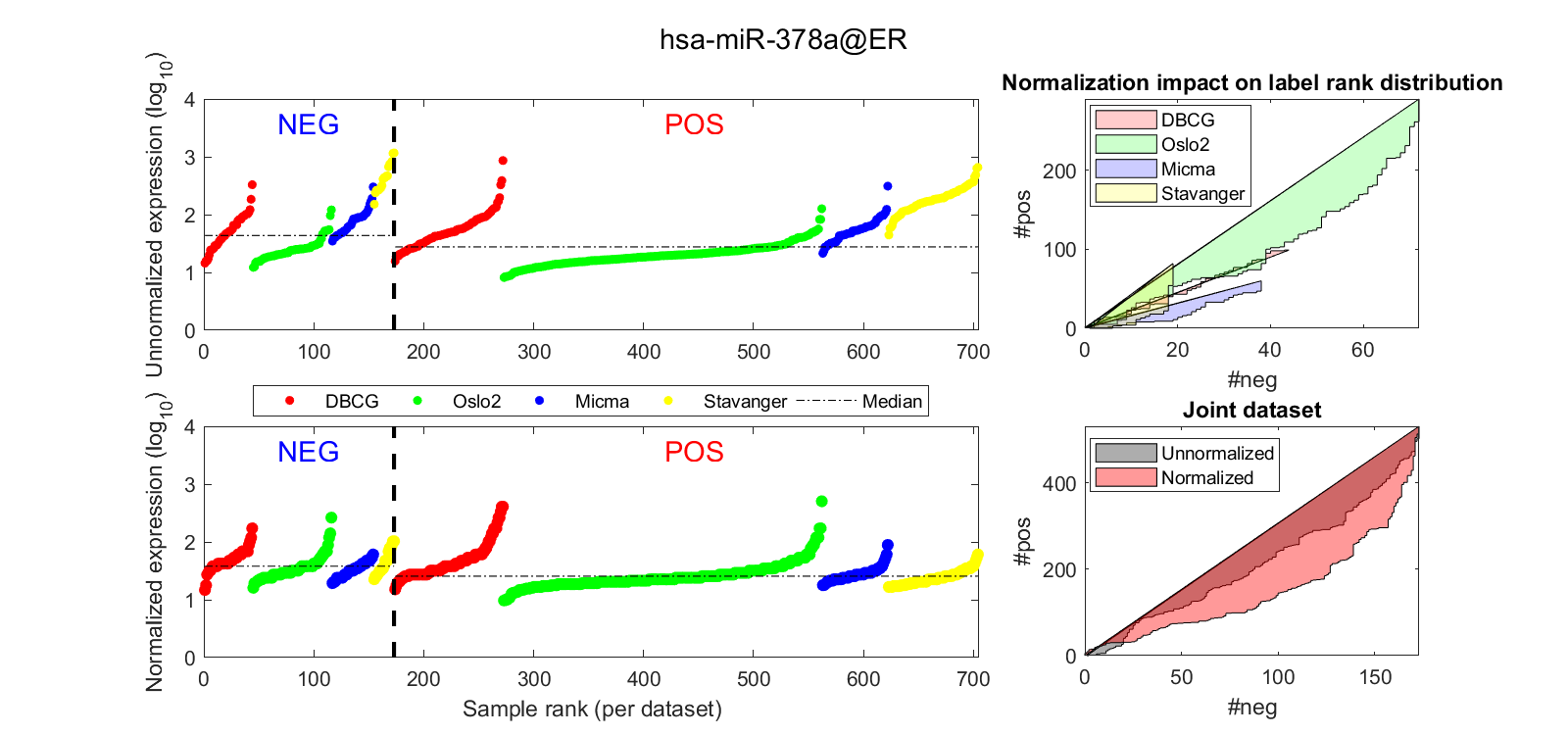

Supplement: S1 Data — Contains additional figures per miRNA pertaining to the analysis presented in Fig 2. (ZIP) [file pcbi.1008608.s001.zip › hsa-miR-378a.png]

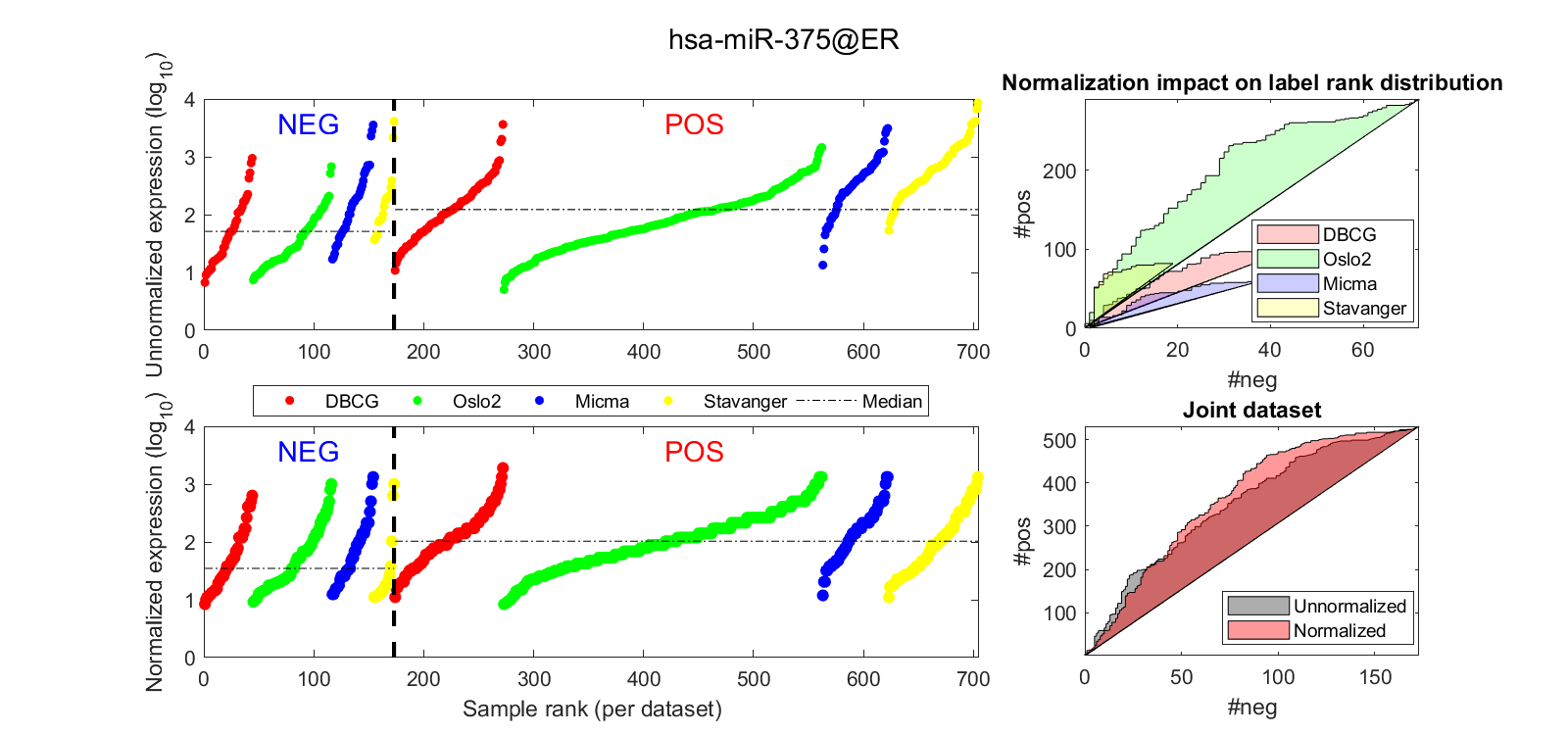

Supplement: S1 Data — Contains additional figures per miRNA pertaining to the analysis presented in Fig 2. (ZIP) [file pcbi.1008608.s001.zip › hsa-miR-375.png]

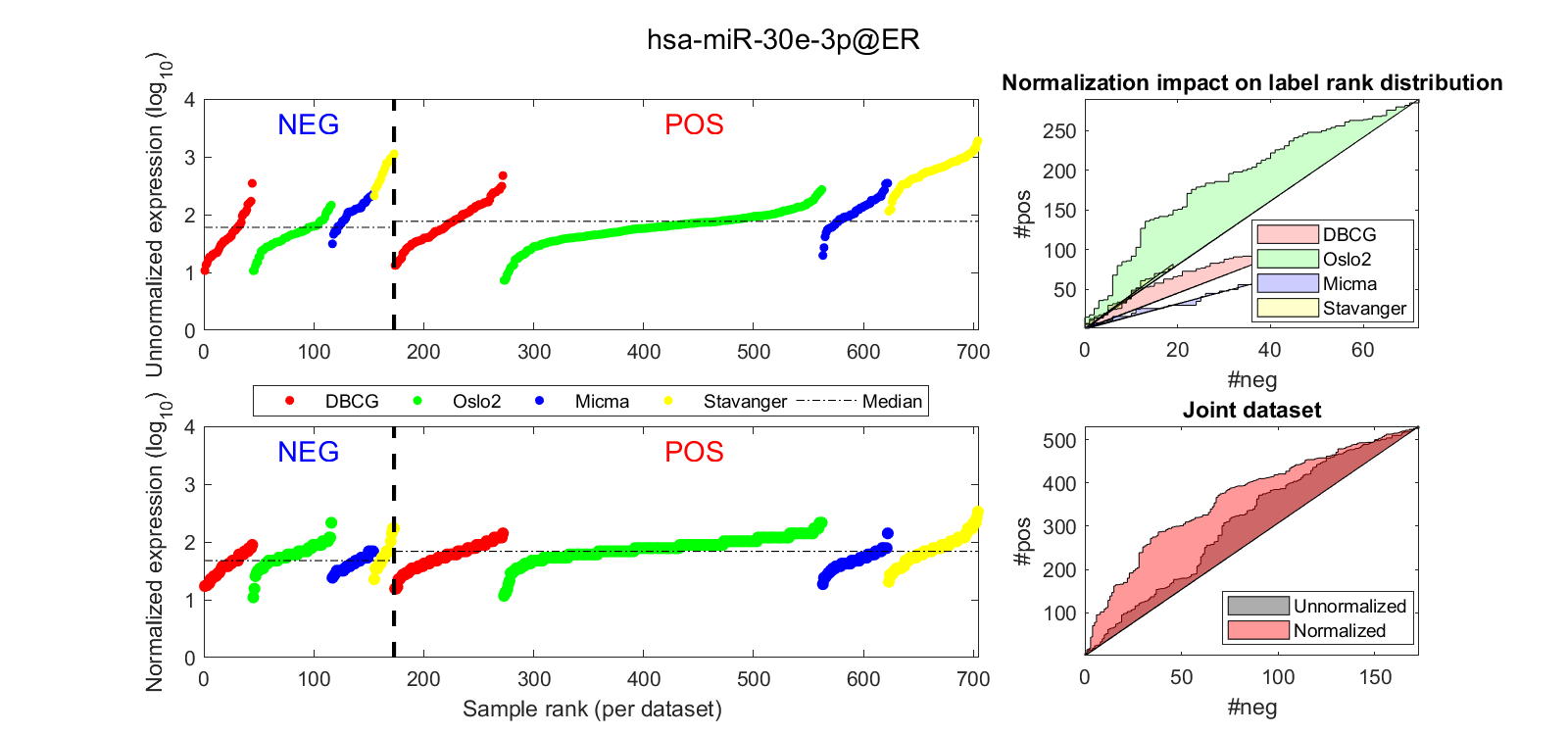

Supplement: S1 Data — Contains additional figures per miRNA pertaining to the analysis presented in Fig 2. (ZIP) [file pcbi.1008608.s001.zip › hsa-miR-30e-3p.png]

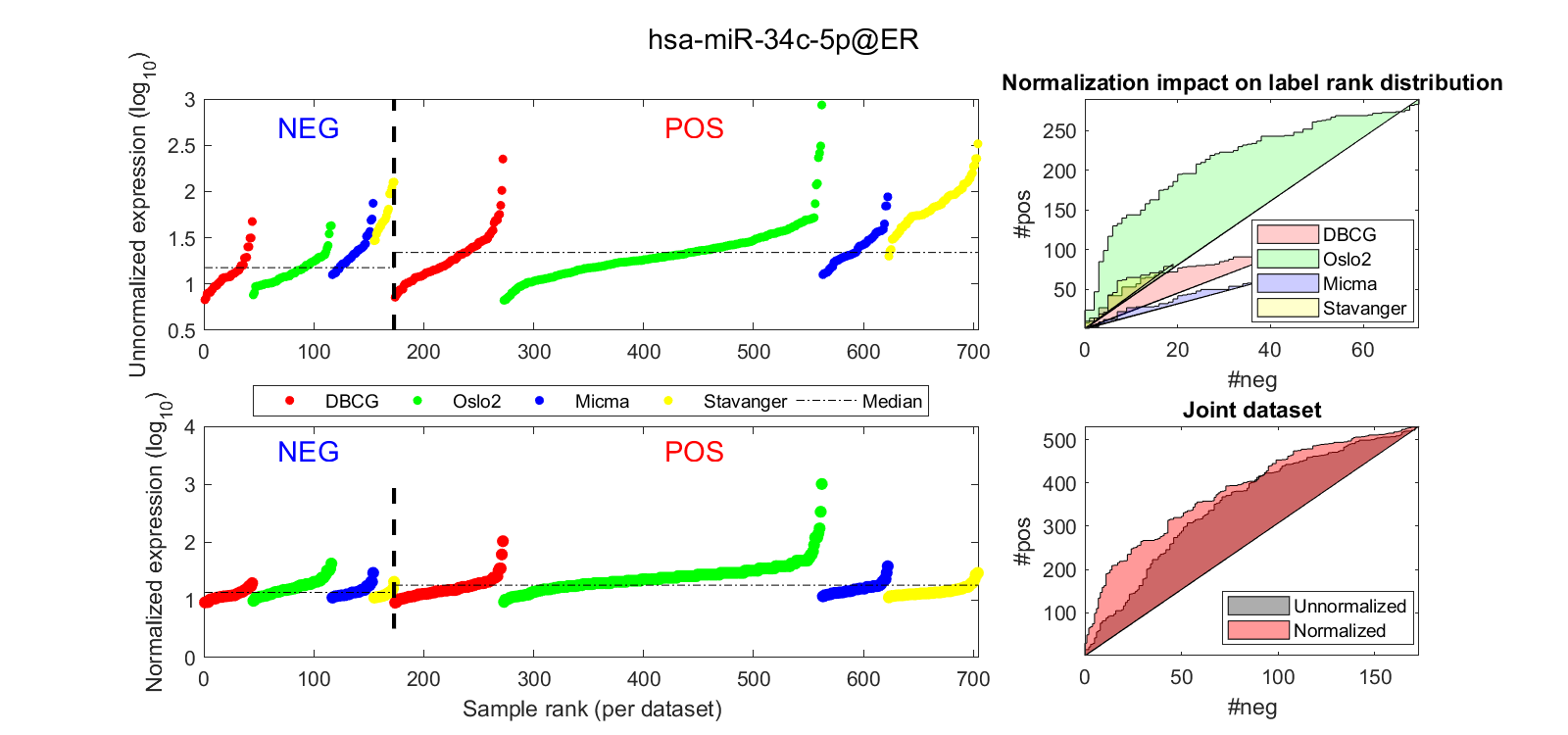

Supplement: S1 Data — Contains additional figures per miRNA pertaining to the analysis presented in Fig 2. (ZIP) [file pcbi.1008608.s001.zip › hsa-miR-34c-5p.png]

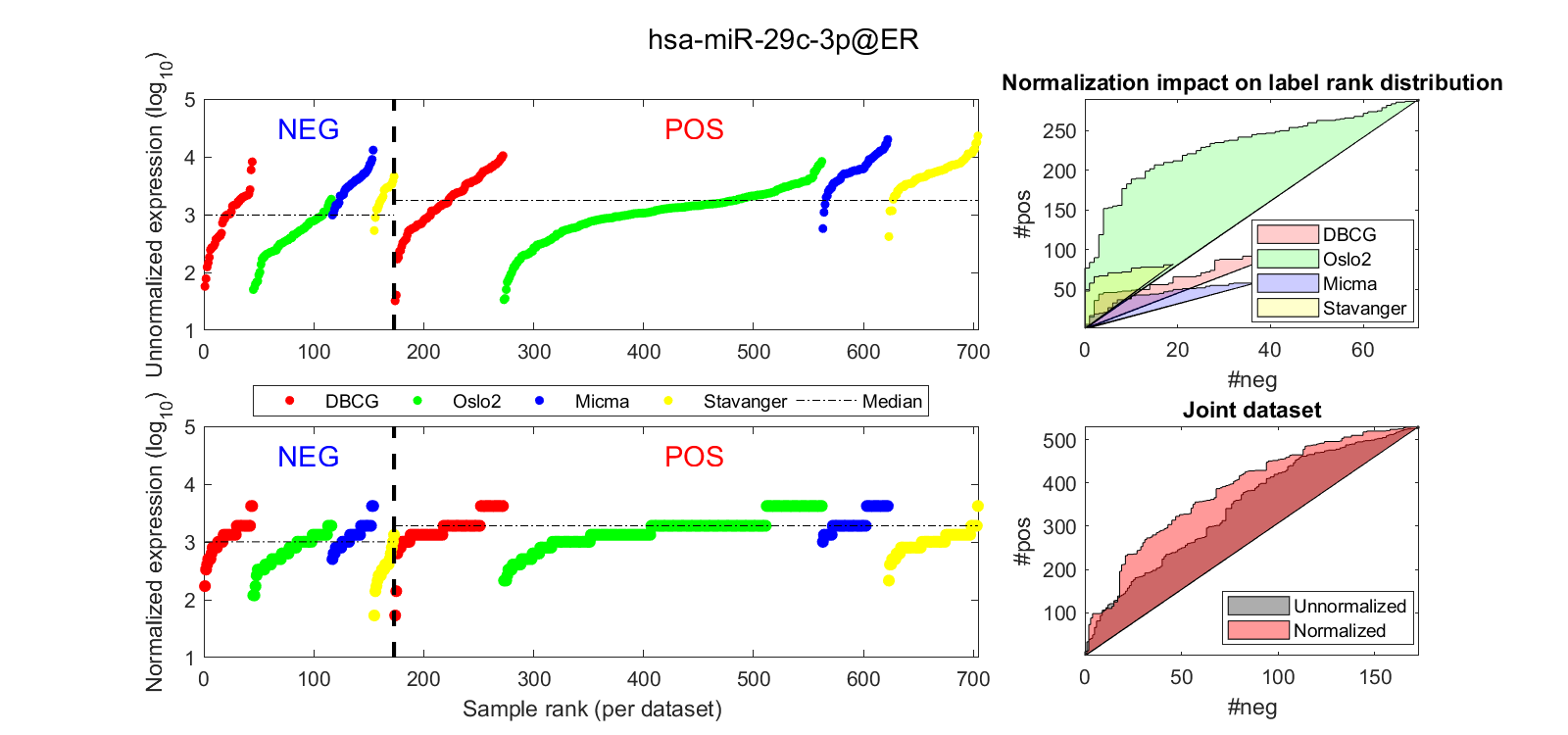

Supplement: S1 Data — Contains additional figures per miRNA pertaining to the analysis presented in Fig 2. (ZIP) [file pcbi.1008608.s001.zip › hsa-miR-29c-3p.png]

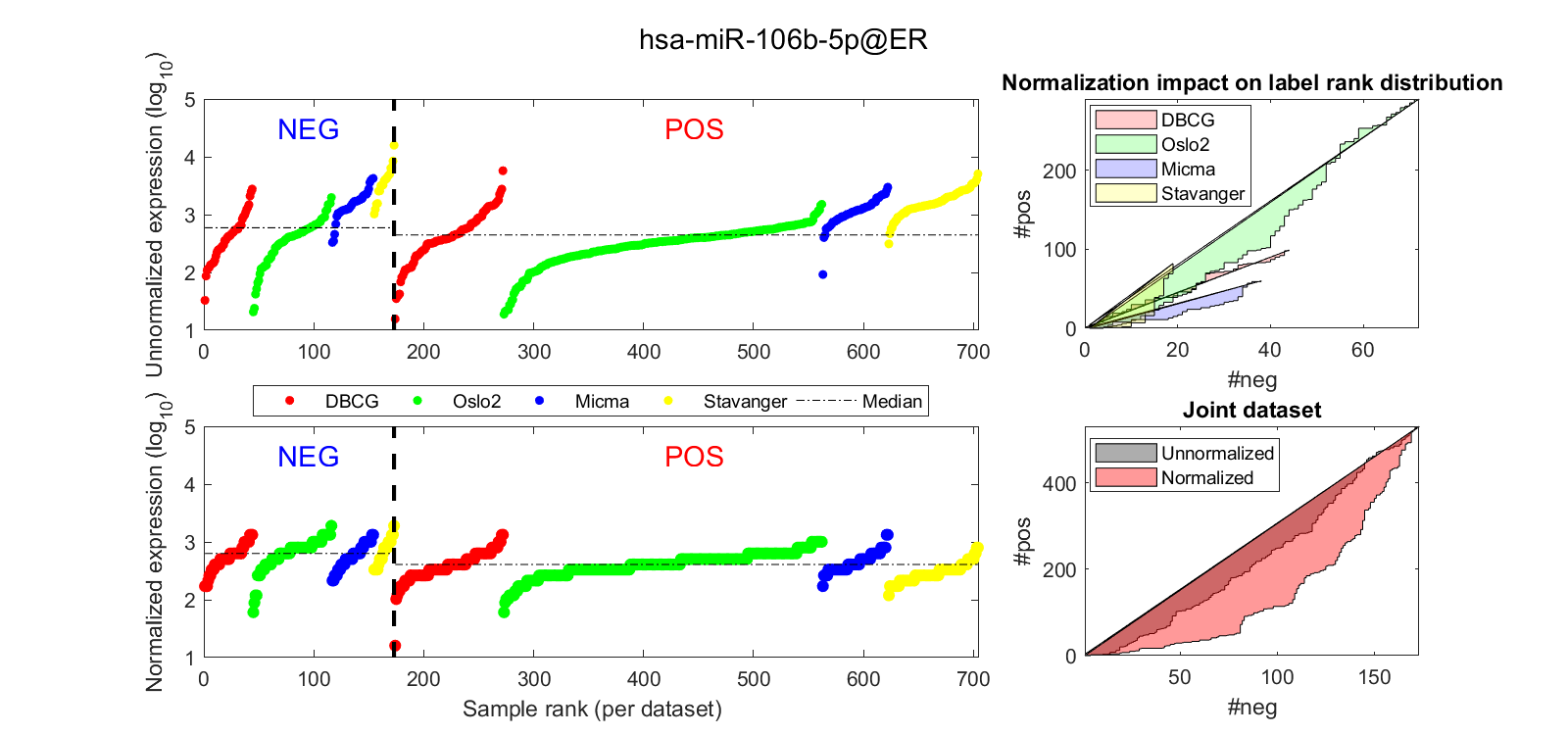

Supplement: S1 Data — Contains additional figures per miRNA pertaining to the analysis presented in Fig 2. (ZIP) [file pcbi.1008608.s001.zip › hsa-miR-106b-5p.png]

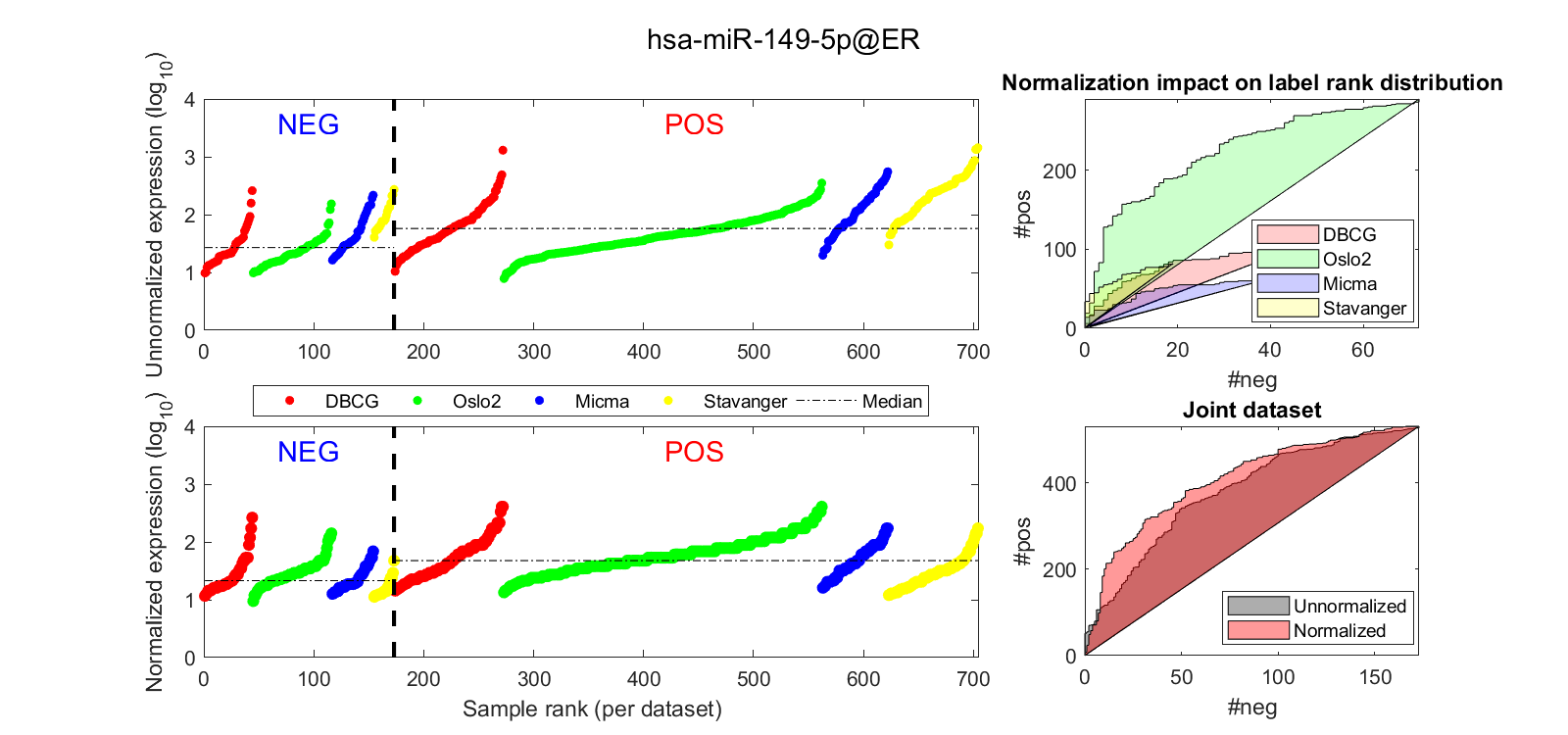

Supplement: S1 Data — Contains additional figures per miRNA pertaining to the analysis presented in Fig 2. (ZIP) [file pcbi.1008608.s001.zip › hsa-miR-149-5p.png]

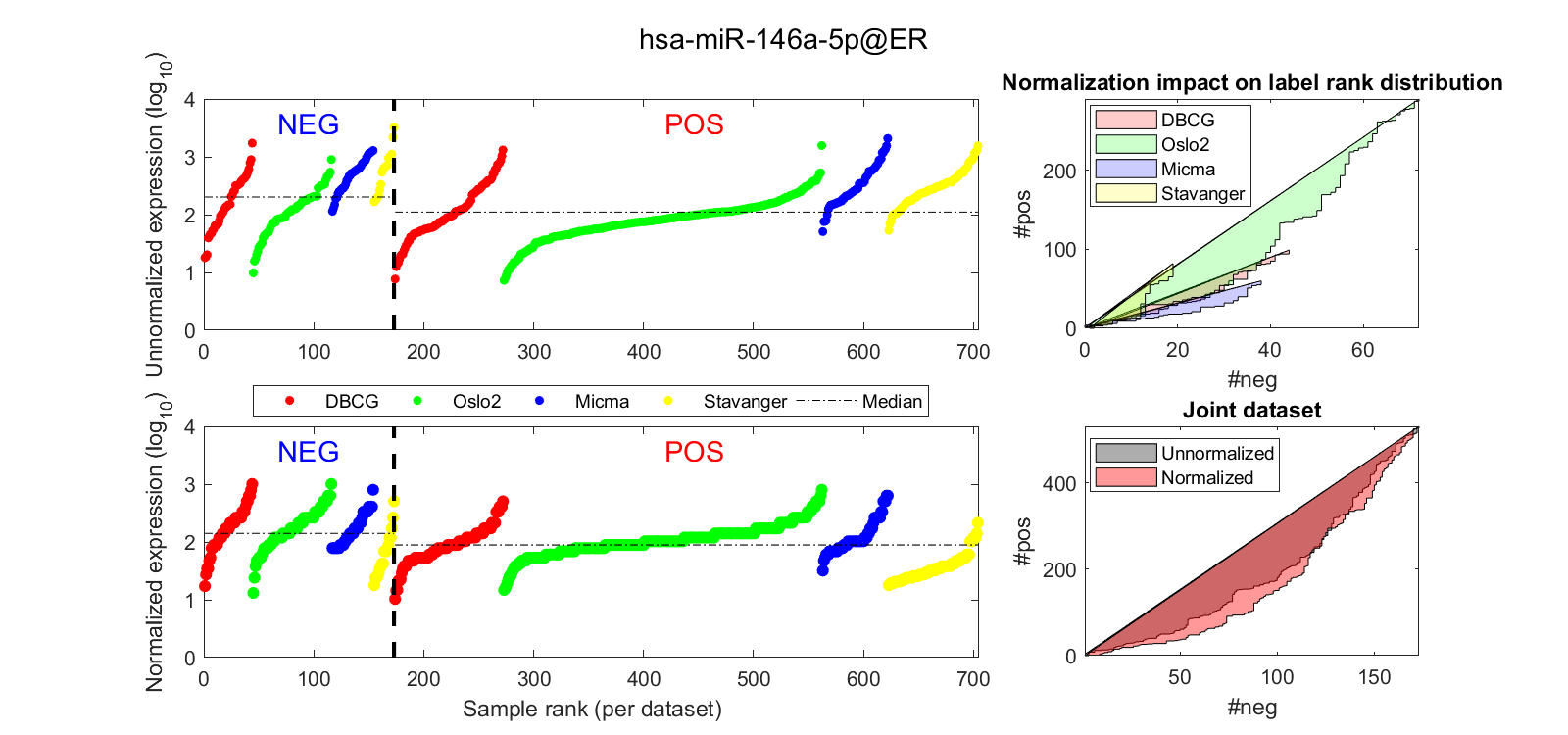

Supplement: S1 Data — Contains additional figures per miRNA pertaining to the analysis presented in Fig 2. (ZIP) [file pcbi.1008608.s001.zip › hsa-miR-146a-5p.png]

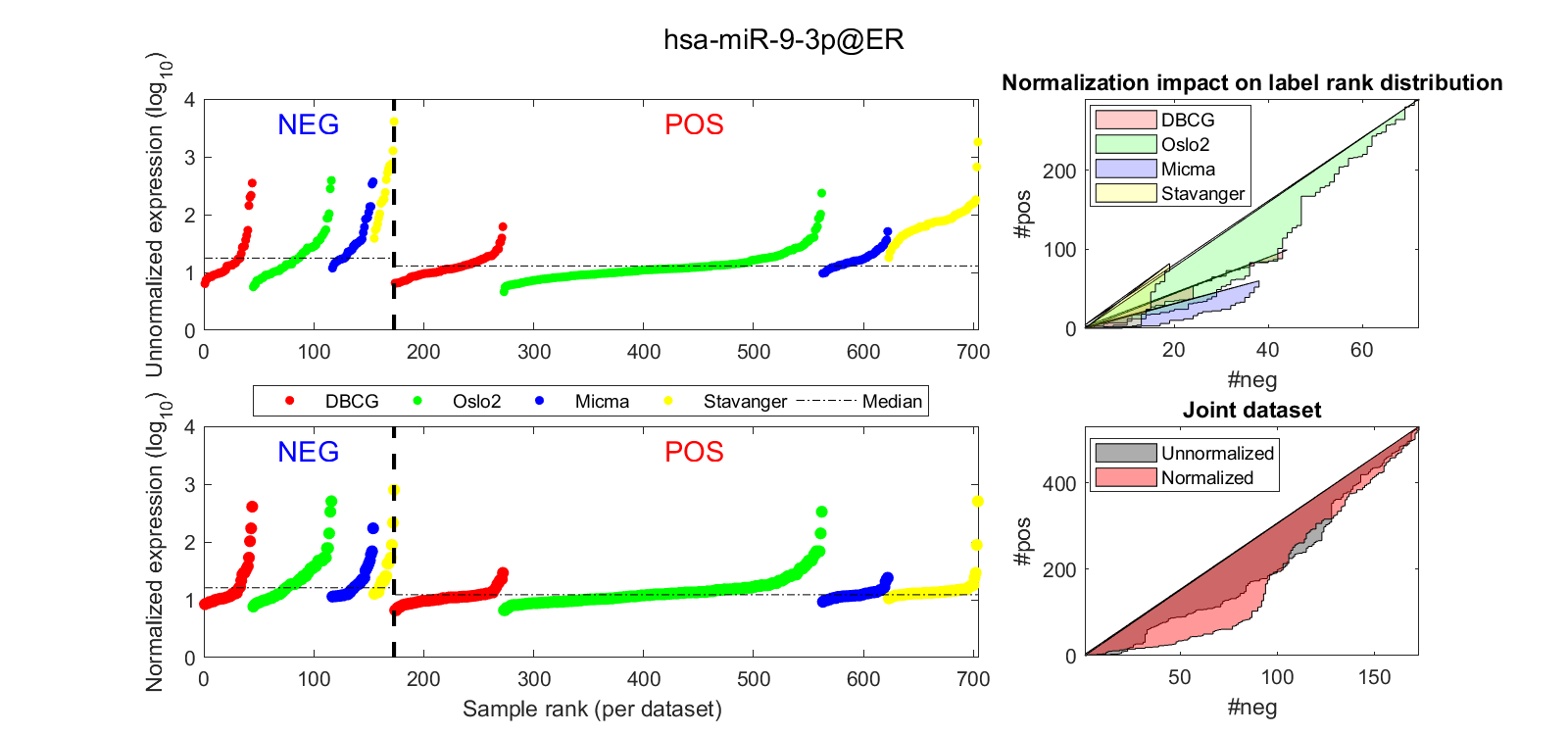

Supplement: S1 Data — Contains additional figures per miRNA pertaining to the analysis presented in Fig 2. (ZIP) [file pcbi.1008608.s001.zip › hsa-miR-9-3p.png]

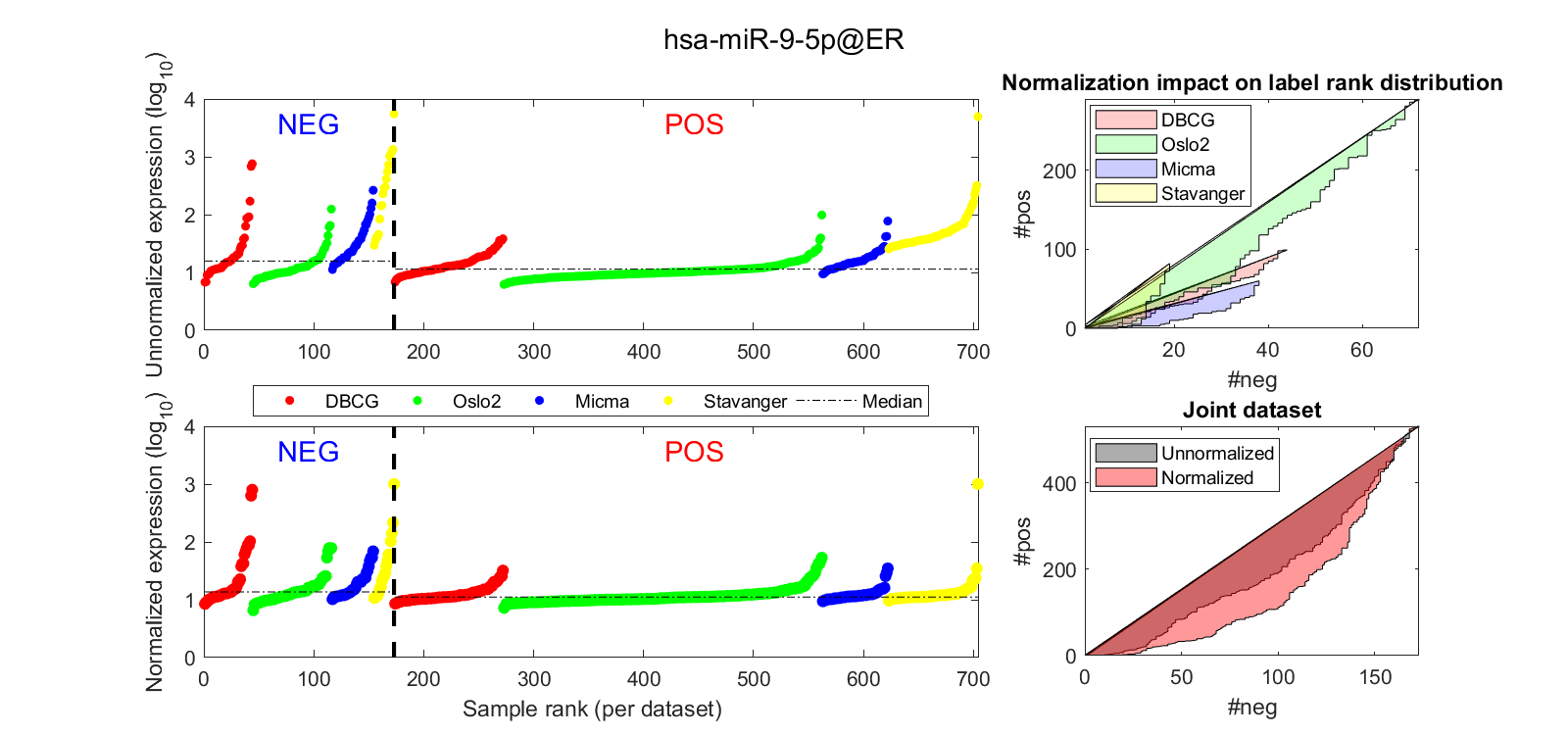

Supplement: S1 Data — Contains additional figures per miRNA pertaining to the analysis presented in Fig 2. (ZIP) [file pcbi.1008608.s001.zip › hsa-miR-9-5p.png]

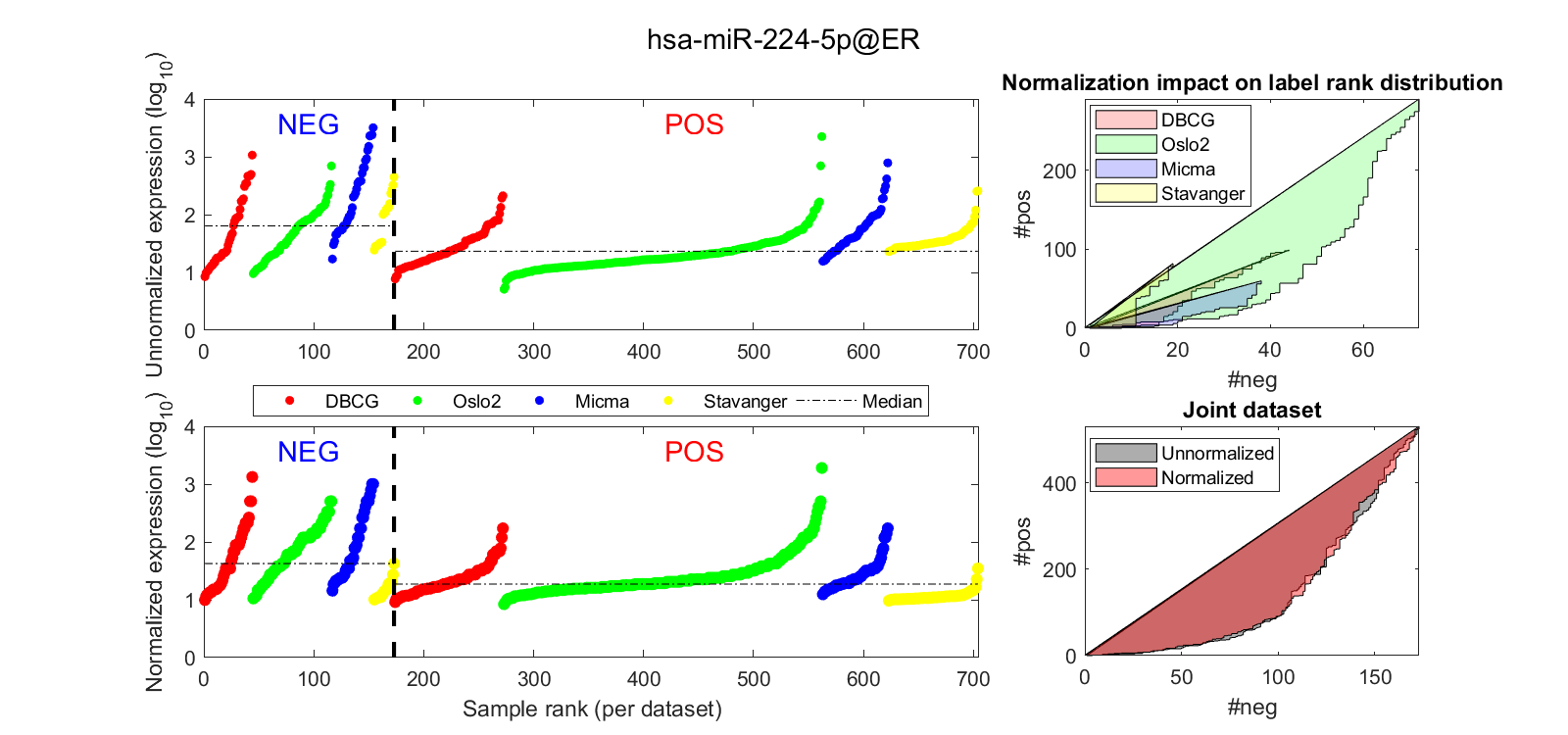

Supplement: S1 Data — Contains additional figures per miRNA pertaining to the analysis presented in Fig 2. (ZIP) [file pcbi.1008608.s001.zip › hsa-miR-224-5p.png]

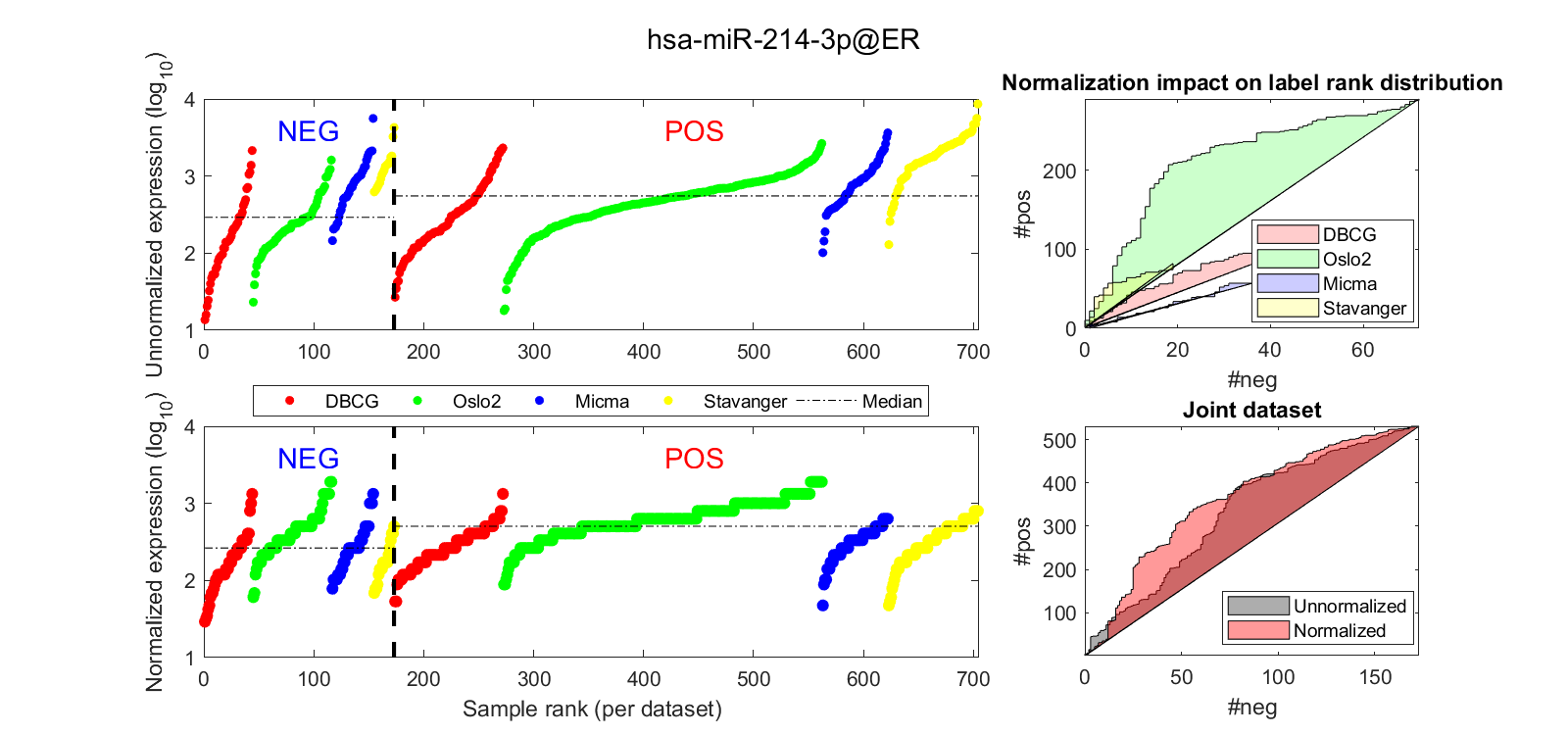

Supplement: S1 Data — Contains additional figures per miRNA pertaining to the analysis presented in Fig 2. (ZIP) [file pcbi.1008608.s001.zip › hsa-miR-214-3p.png]

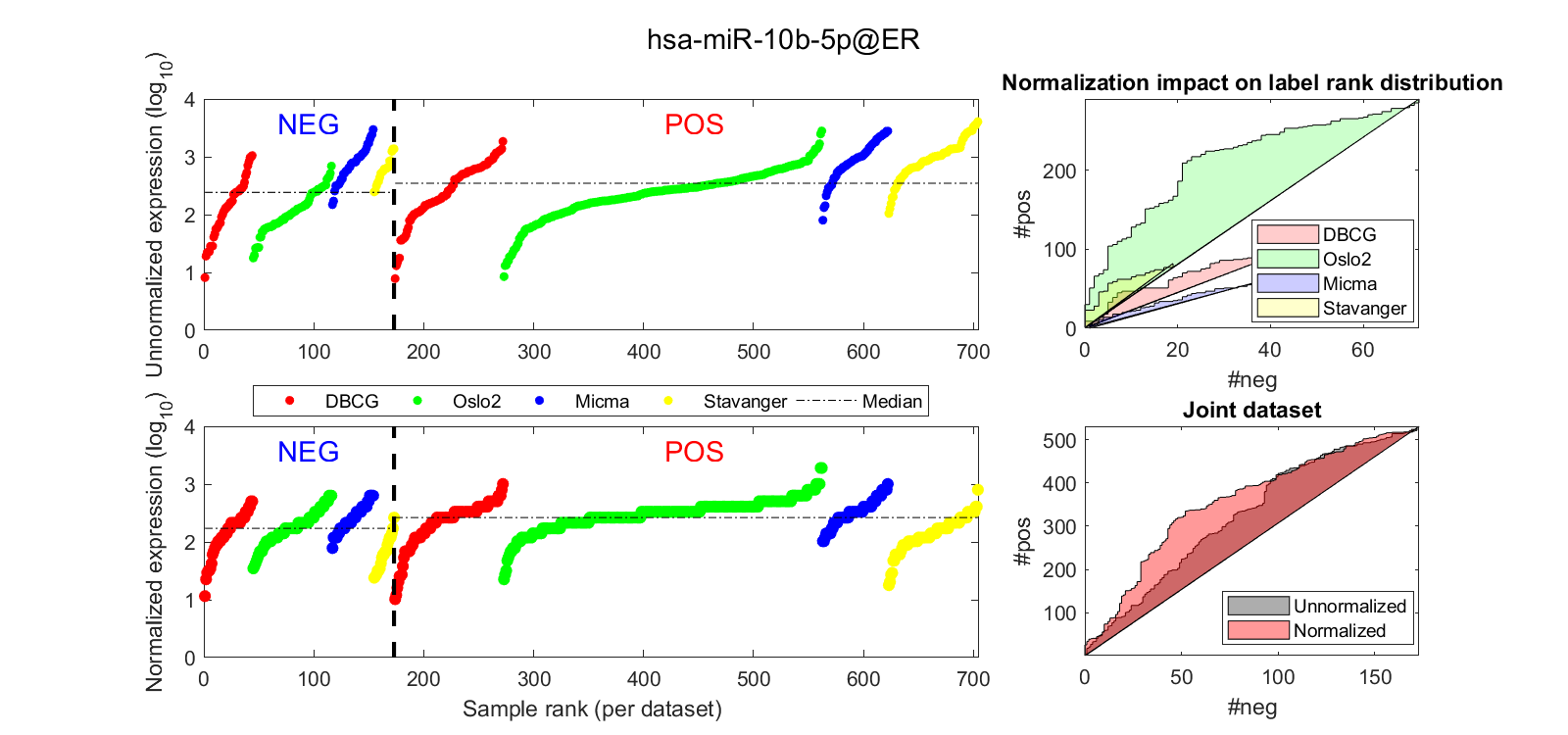

Supplement: S1 Data — Contains additional figures per miRNA pertaining to the analysis presented in Fig 2. (ZIP) [file pcbi.1008608.s001.zip › hsa-miR-10b-5p.png]

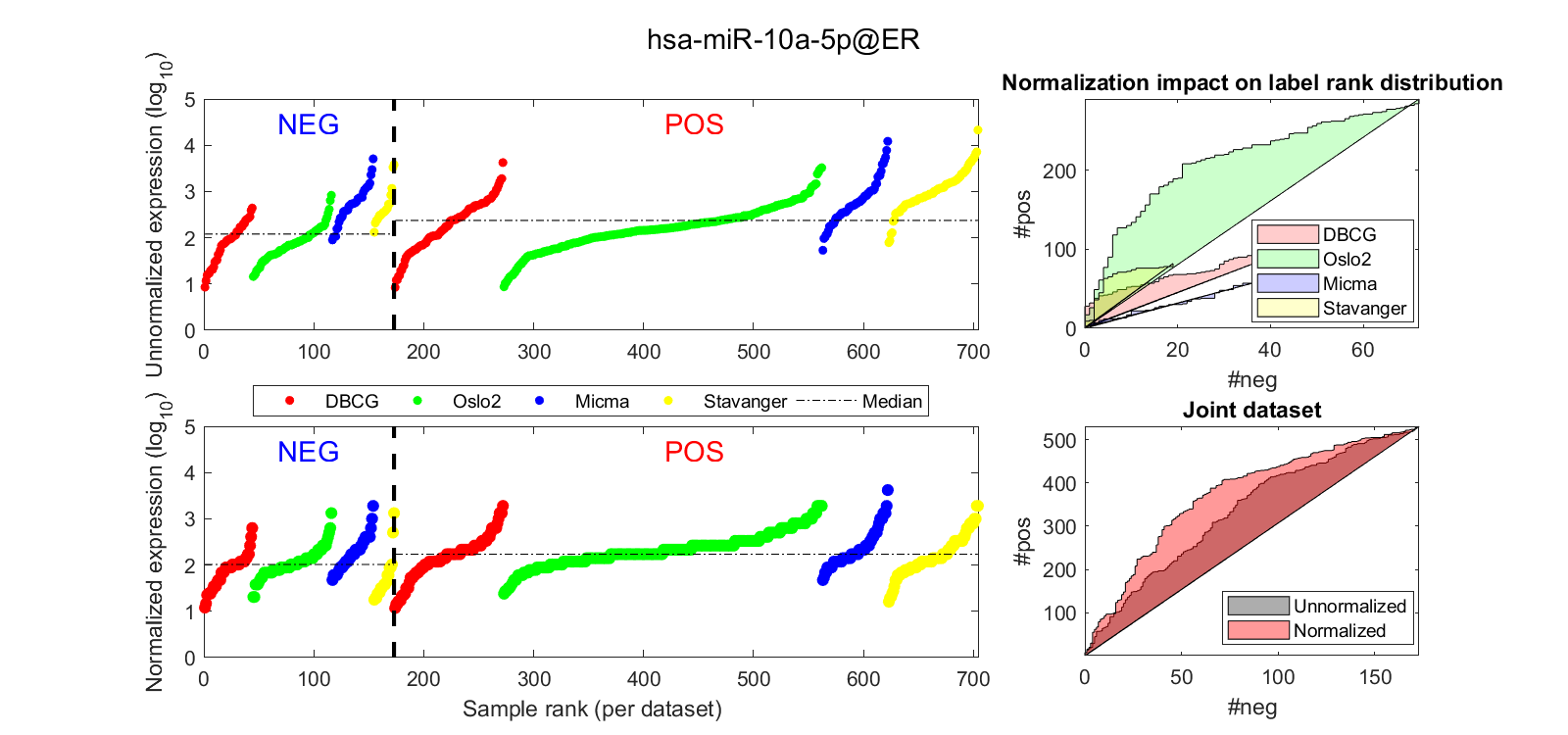

Supplement: S1 Data — Contains additional figures per miRNA pertaining to the analysis presented in Fig 2. (ZIP) [file pcbi.1008608.s001.zip › hsa-miR-10a-5p.png]

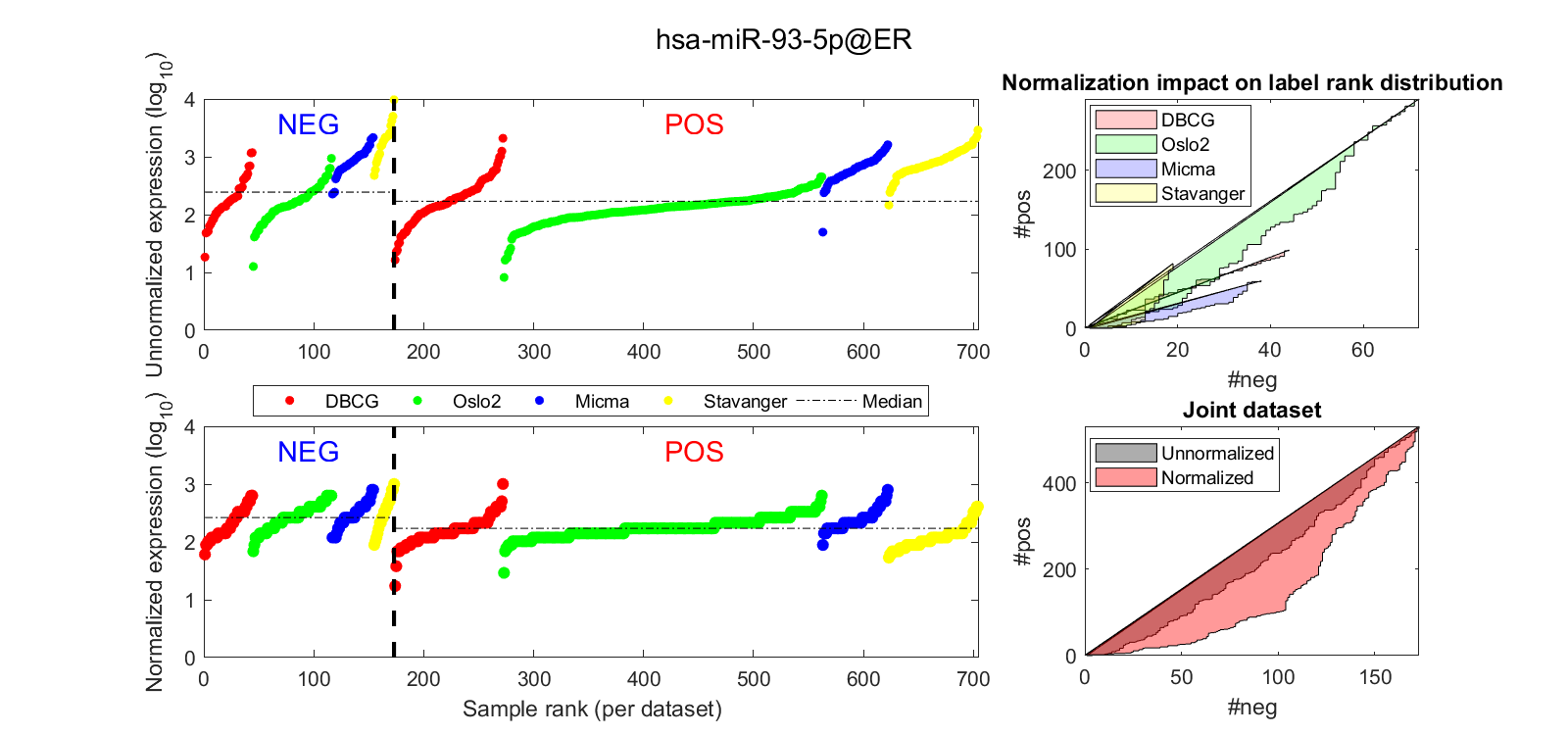

Supplement: S1 Data — Contains additional figures per miRNA pertaining to the analysis presented in Fig 2. (ZIP) [file pcbi.1008608.s001.zip › hsa-miR-93-5p.png]

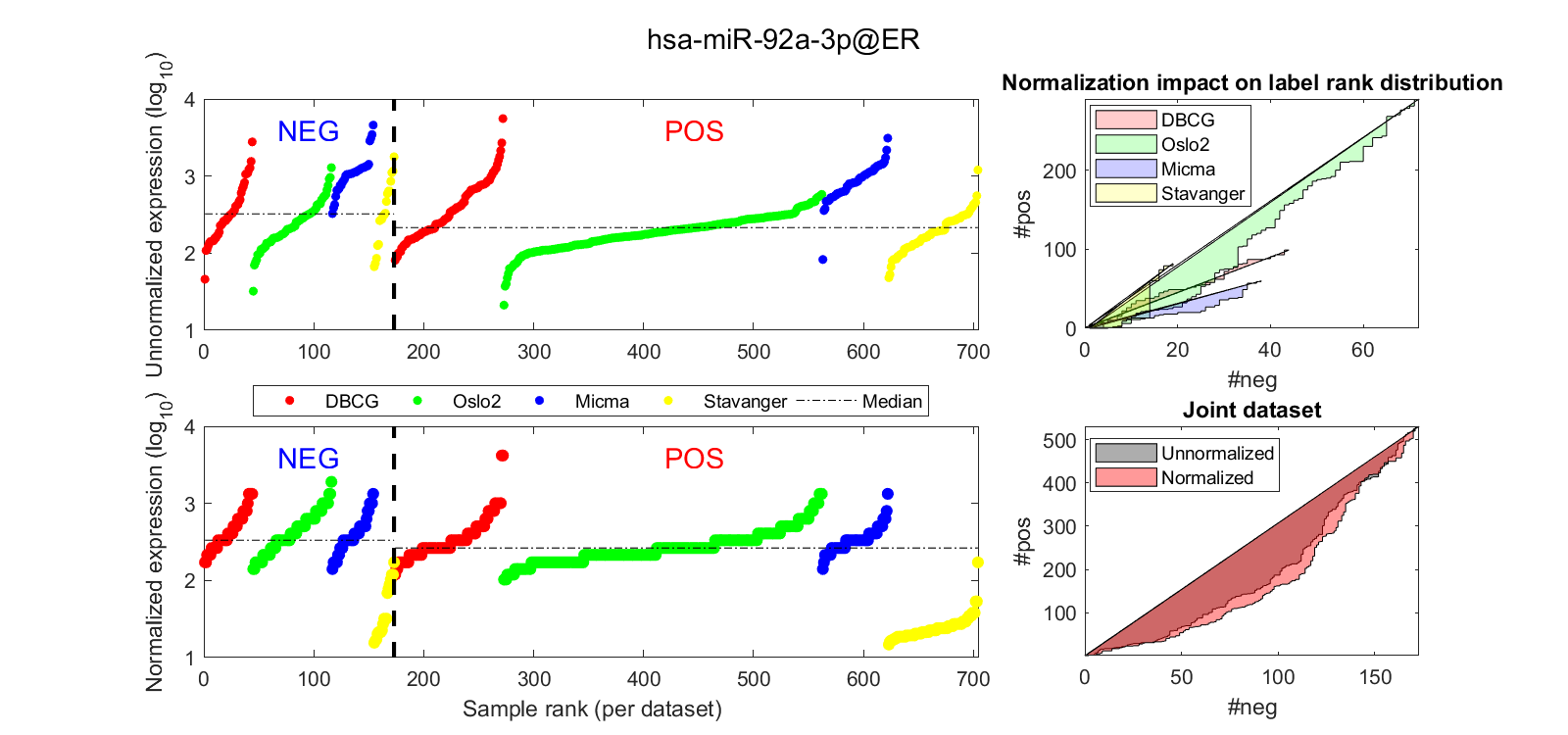

Supplement: S1 Data — Contains additional figures per miRNA pertaining to the analysis presented in Fig 2. (ZIP) [file pcbi.1008608.s001.zip › hsa-miR-92a-3p.png]

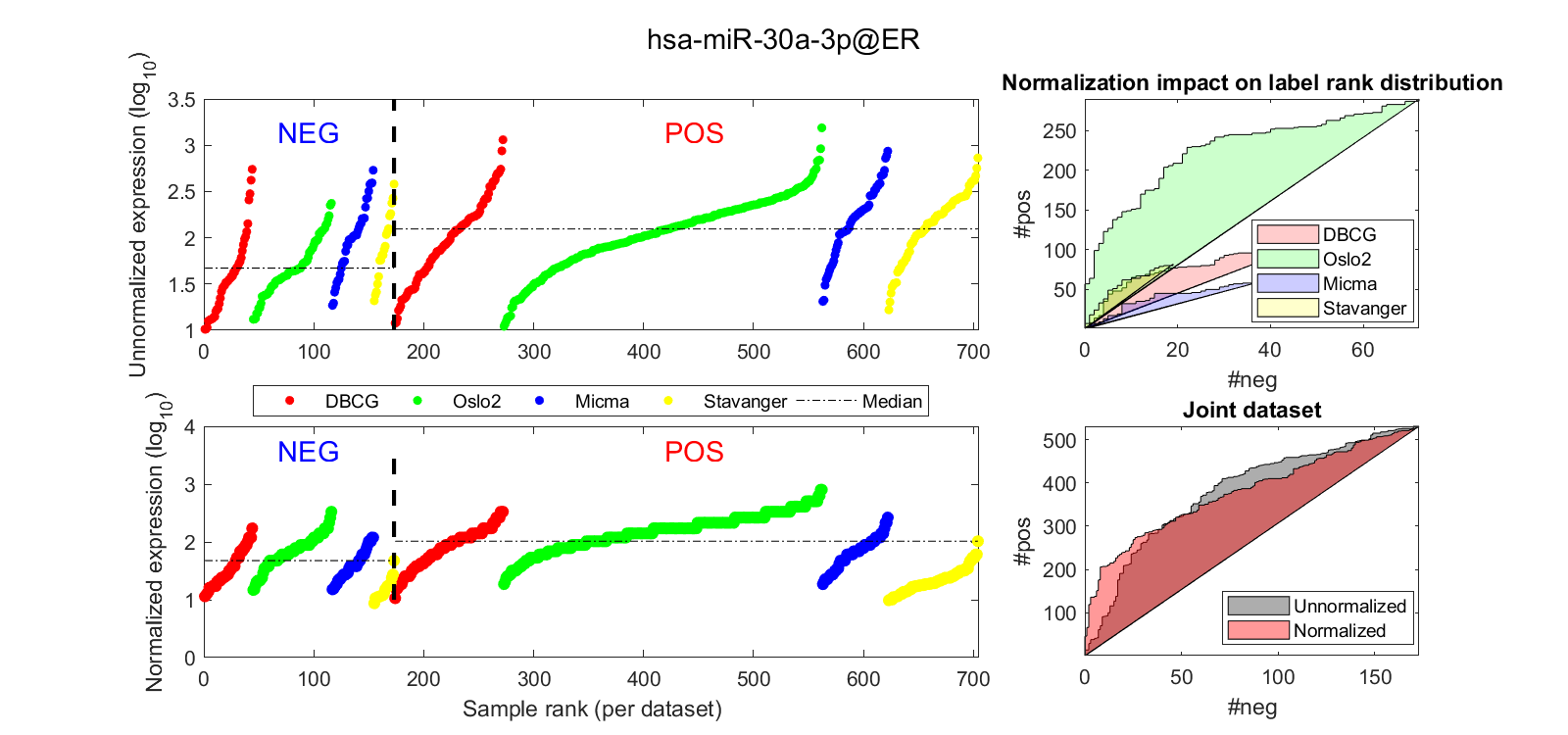

Supplement: S1 Data — Contains additional figures per miRNA pertaining to the analysis presented in Fig 2. (ZIP) [file pcbi.1008608.s001.zip › hsa-miR-30a-3p.png]

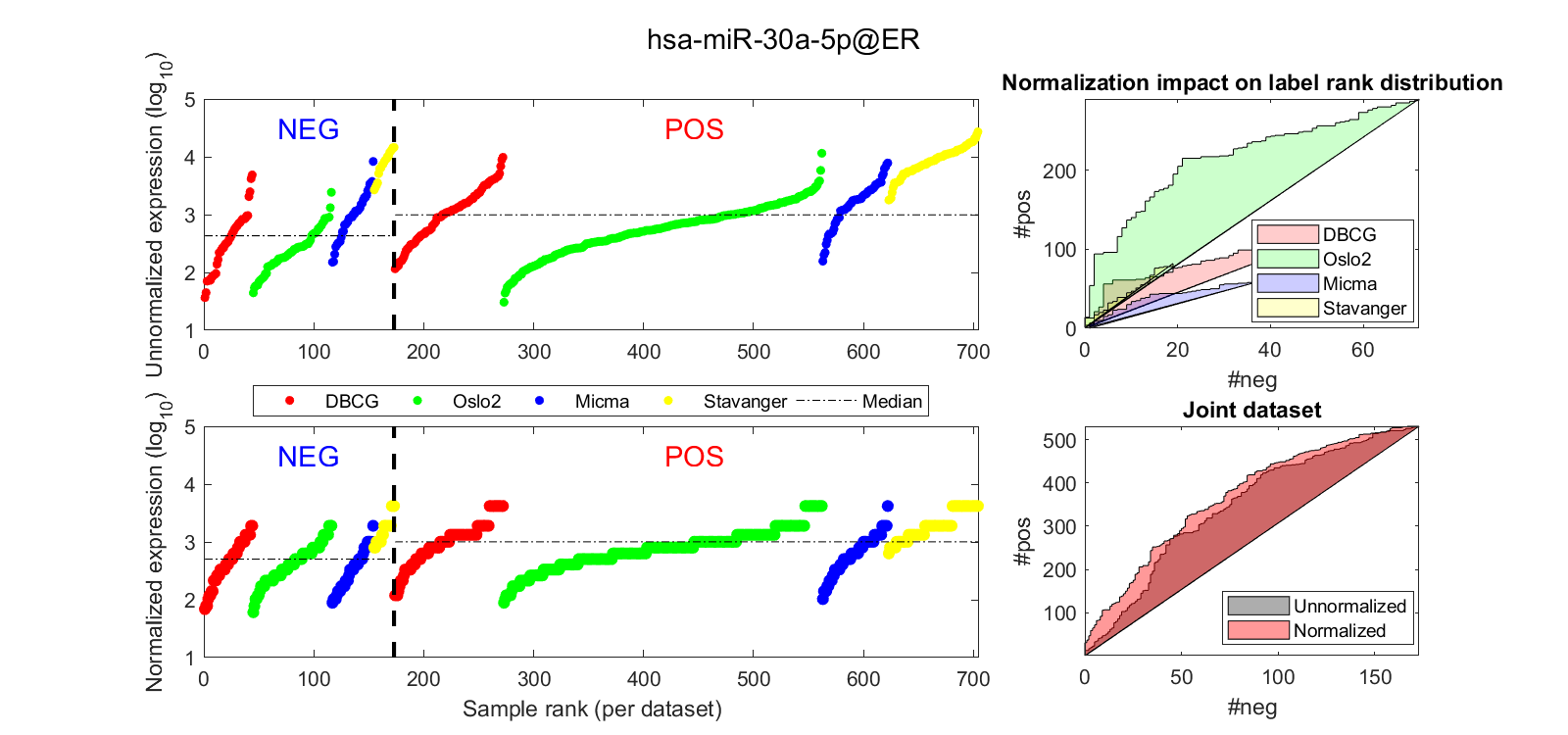

Supplement: S1 Data — Contains additional figures per miRNA pertaining to the analysis presented in Fig 2. (ZIP) [file pcbi.1008608.s001.zip › hsa-miR-30a-5p.png]

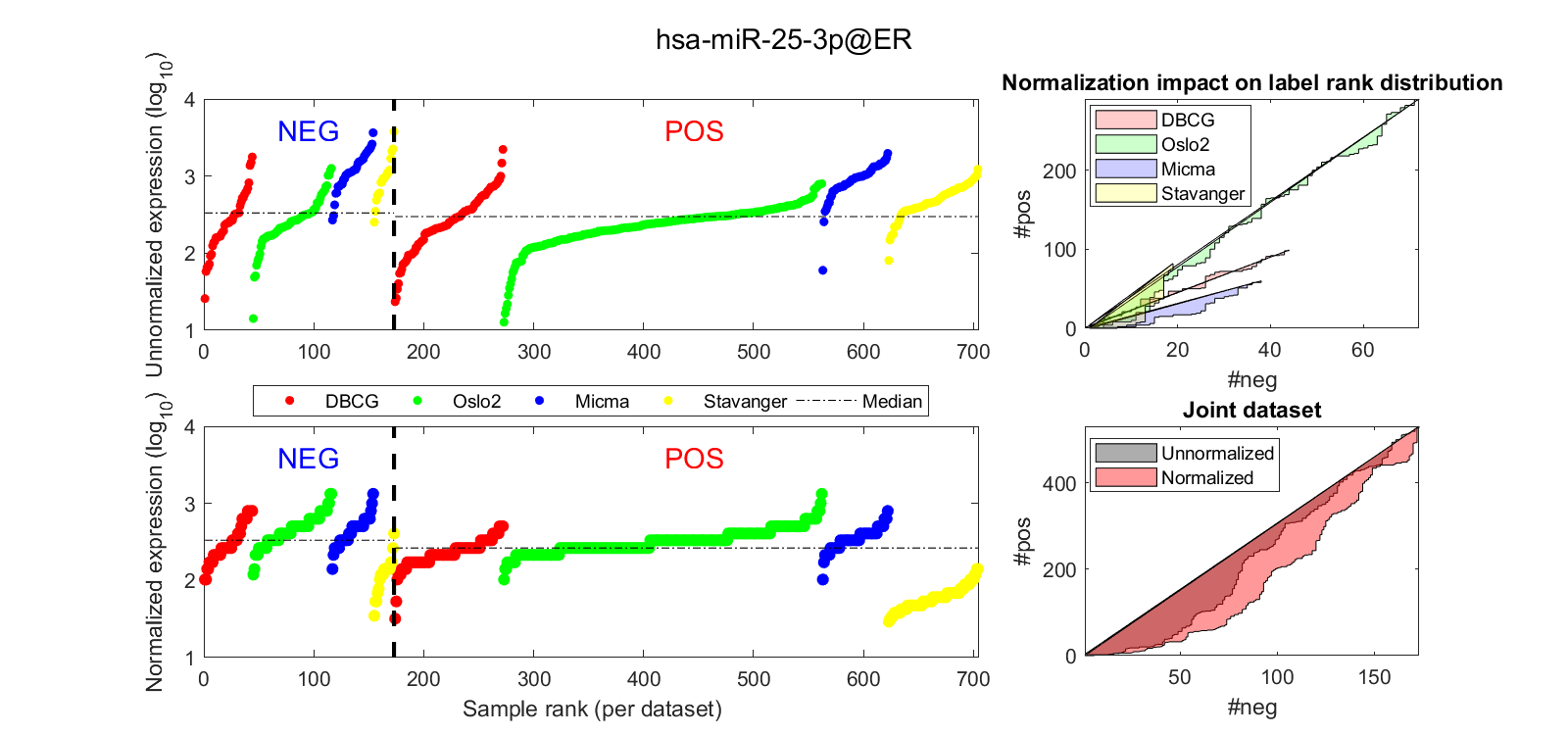

Supplement: S1 Data — Contains additional figures per miRNA pertaining to the analysis presented in Fig 2. (ZIP) [file pcbi.1008608.s001.zip › hsa-miR-25-3p.png]

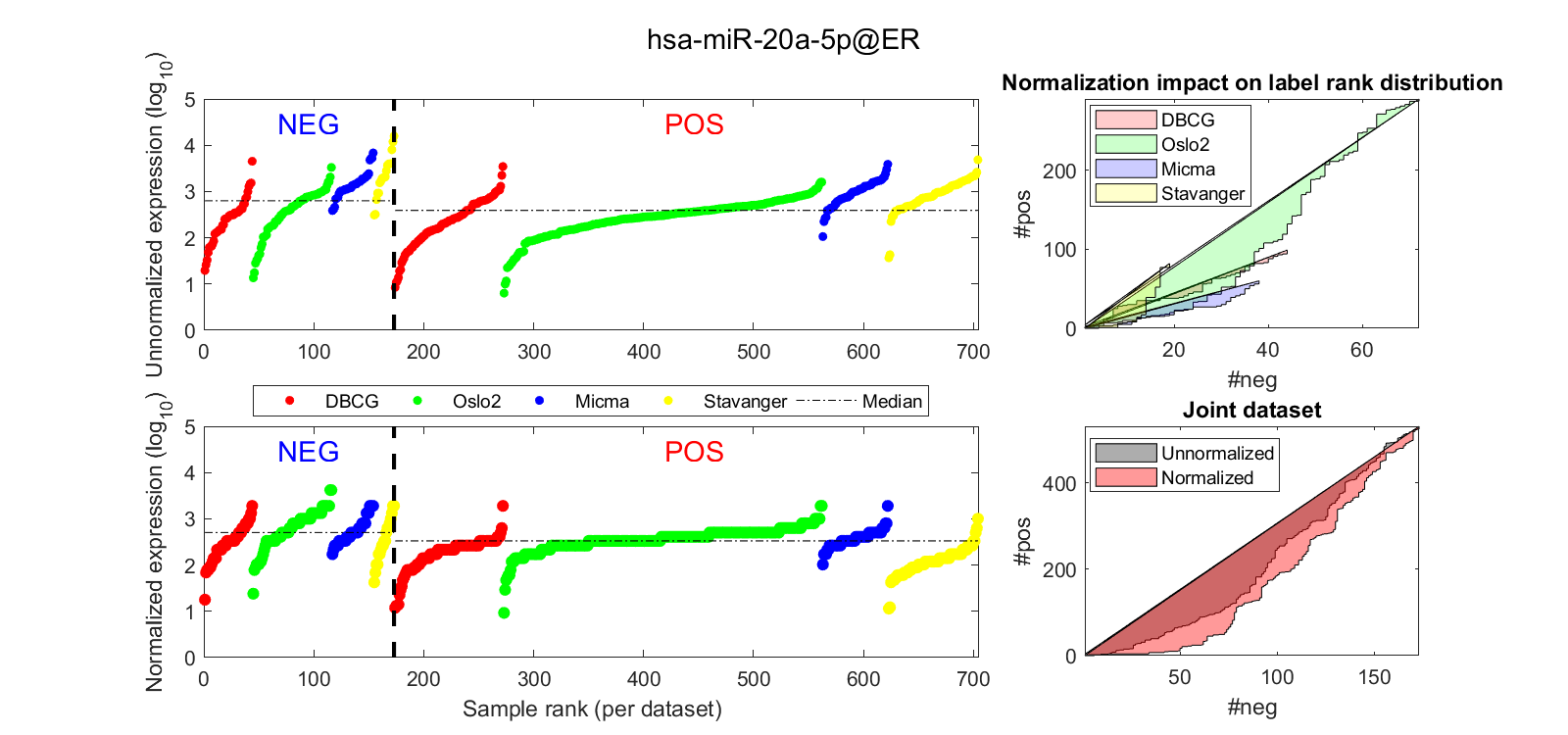

Supplement: S1 Data — Contains additional figures per miRNA pertaining to the analysis presented in Fig 2. (ZIP) [file pcbi.1008608.s001.zip › hsa-miR-20a-5p.png]

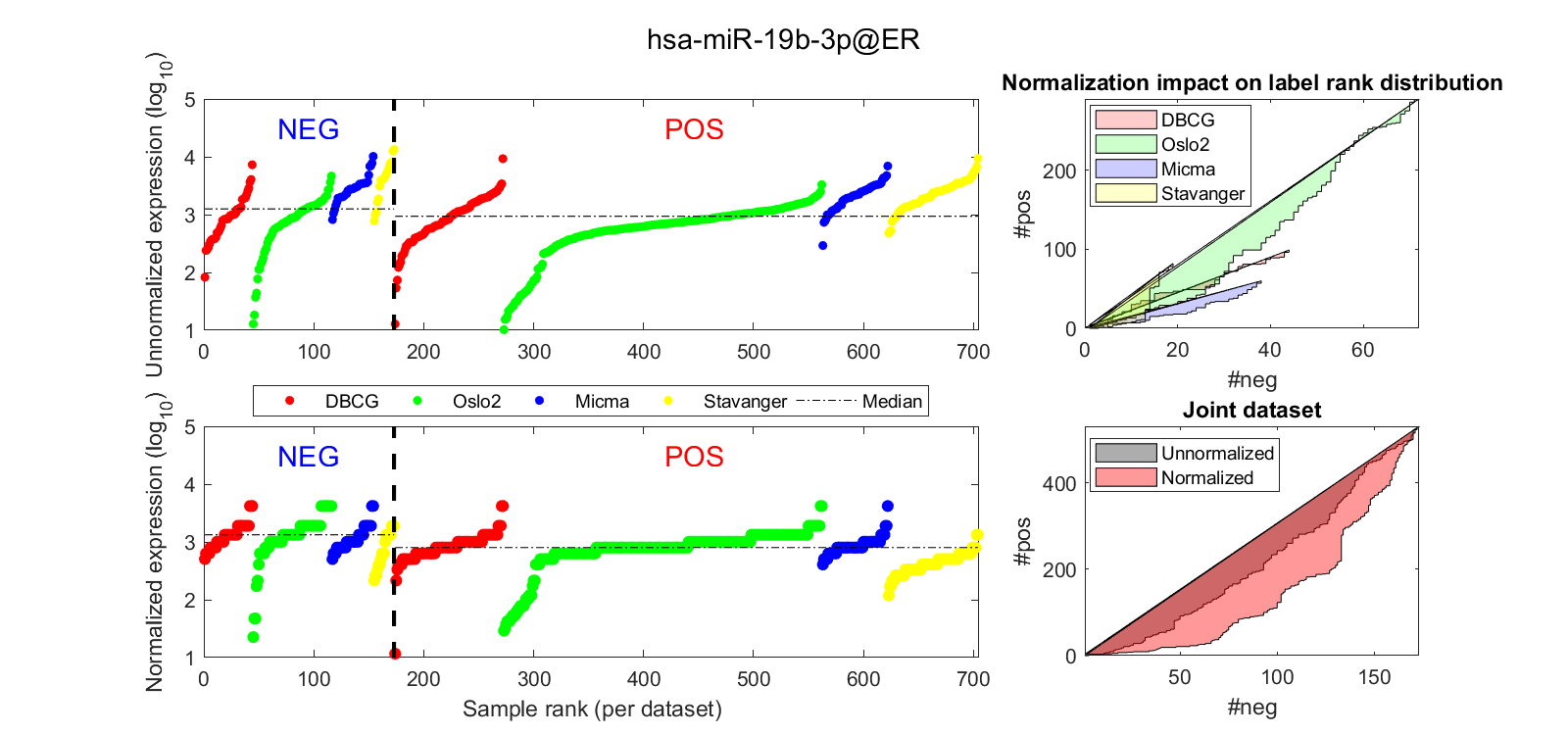

Supplement: S1 Data — Contains additional figures per miRNA pertaining to the analysis presented in Fig 2. (ZIP) [file pcbi.1008608.s001.zip › hsa-miR-19b-3p.png]

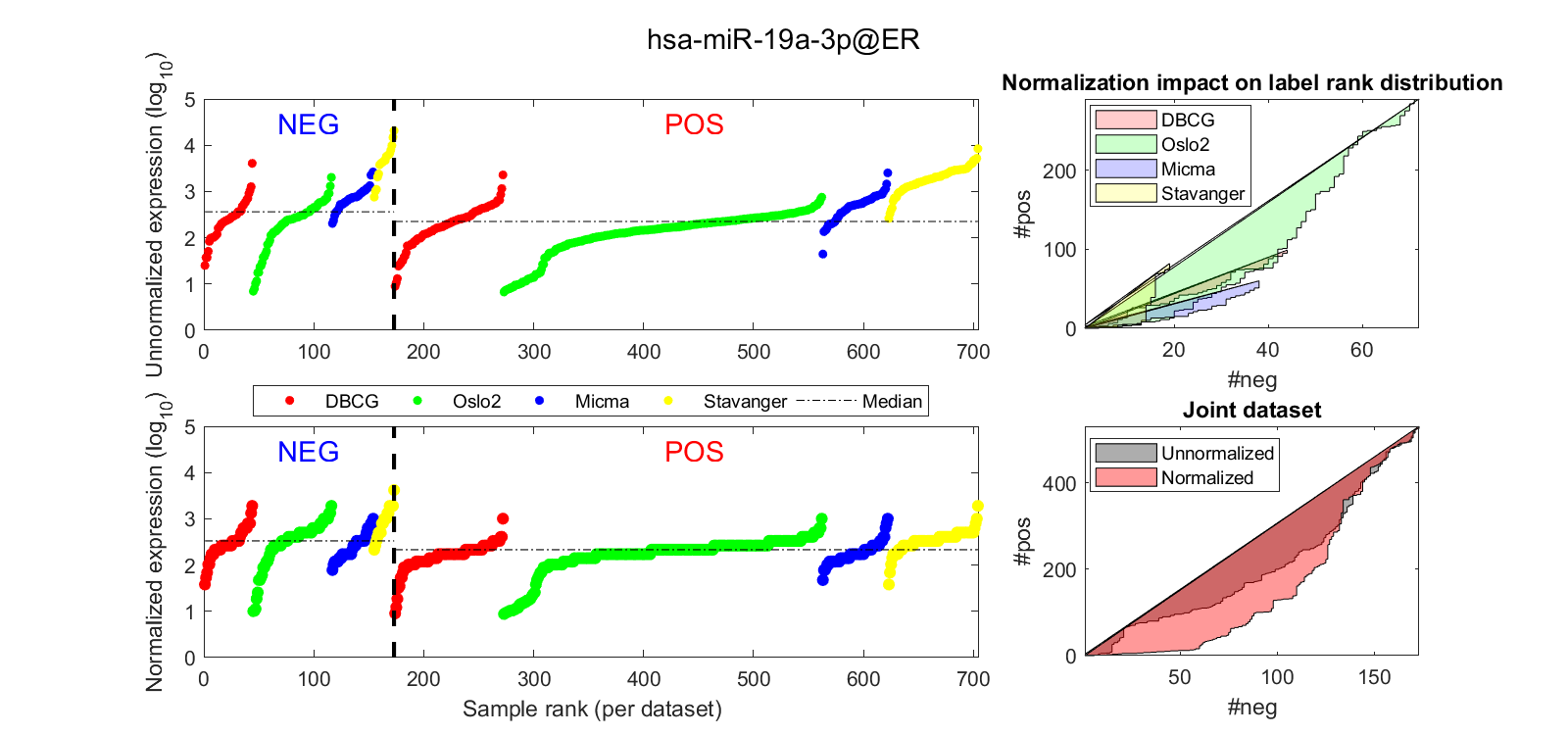

Supplement: S1 Data — Contains additional figures per miRNA pertaining to the analysis presented in Fig 2. (ZIP) [file pcbi.1008608.s001.zip › hsa-miR-19a-3p.png]

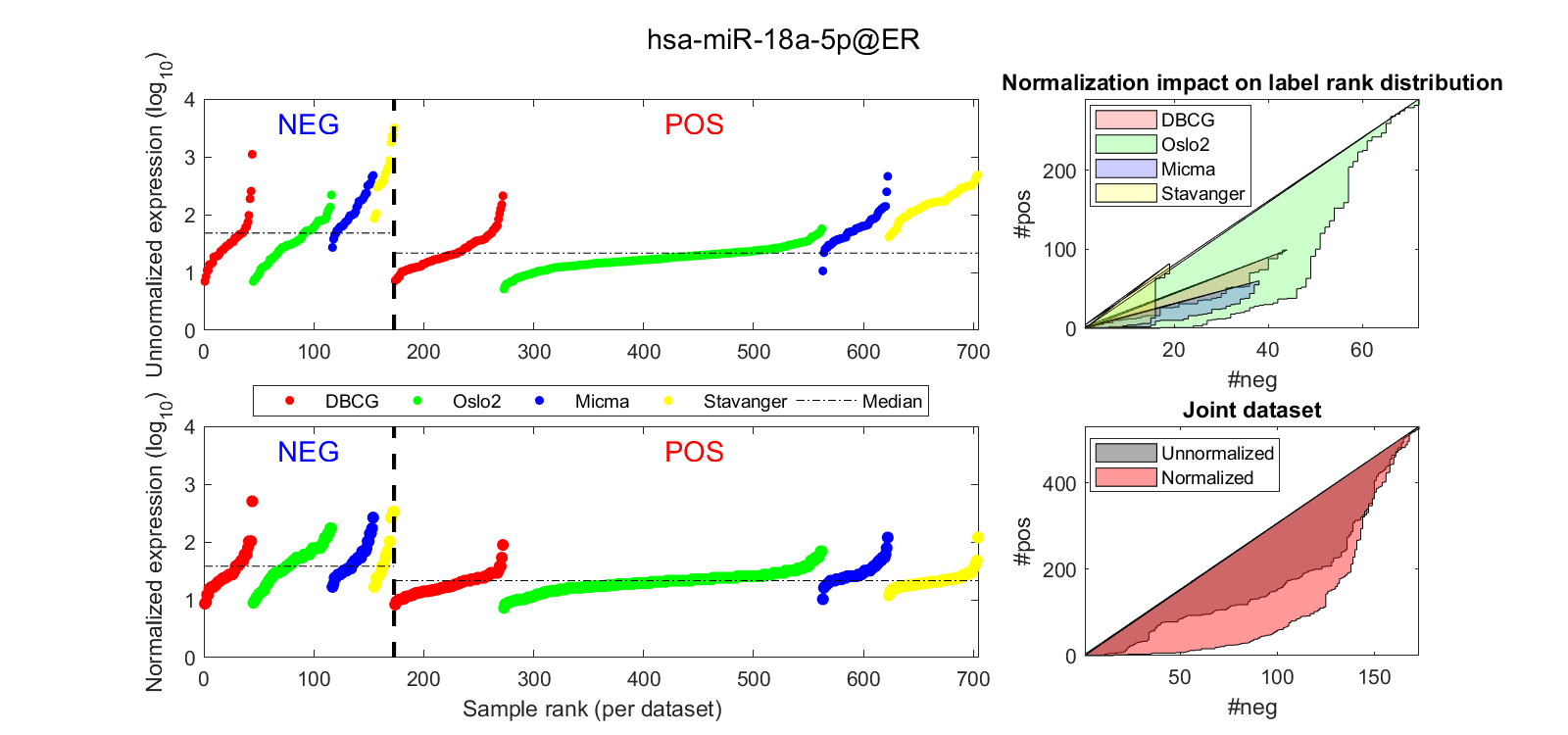

Supplement: S1 Data — Contains additional figures per miRNA pertaining to the analysis presented in Fig 2. (ZIP) [file pcbi.1008608.s001.zip › hsa-miR-18a-5p.png]

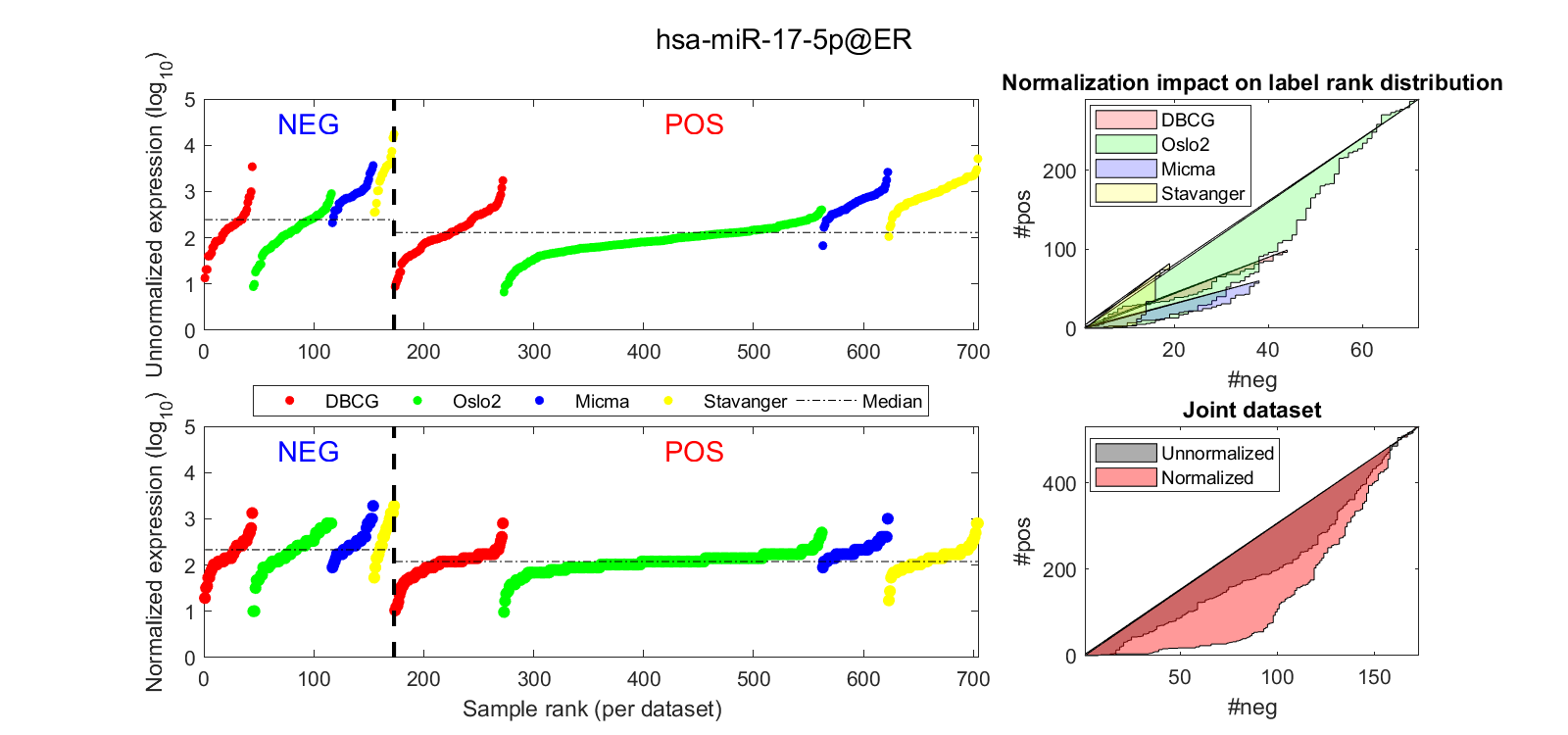

Supplement: S1 Data — Contains additional figures per miRNA pertaining to the analysis presented in Fig 2. (ZIP) [file pcbi.1008608.s001.zip › hsa-miR-17-5p.png]

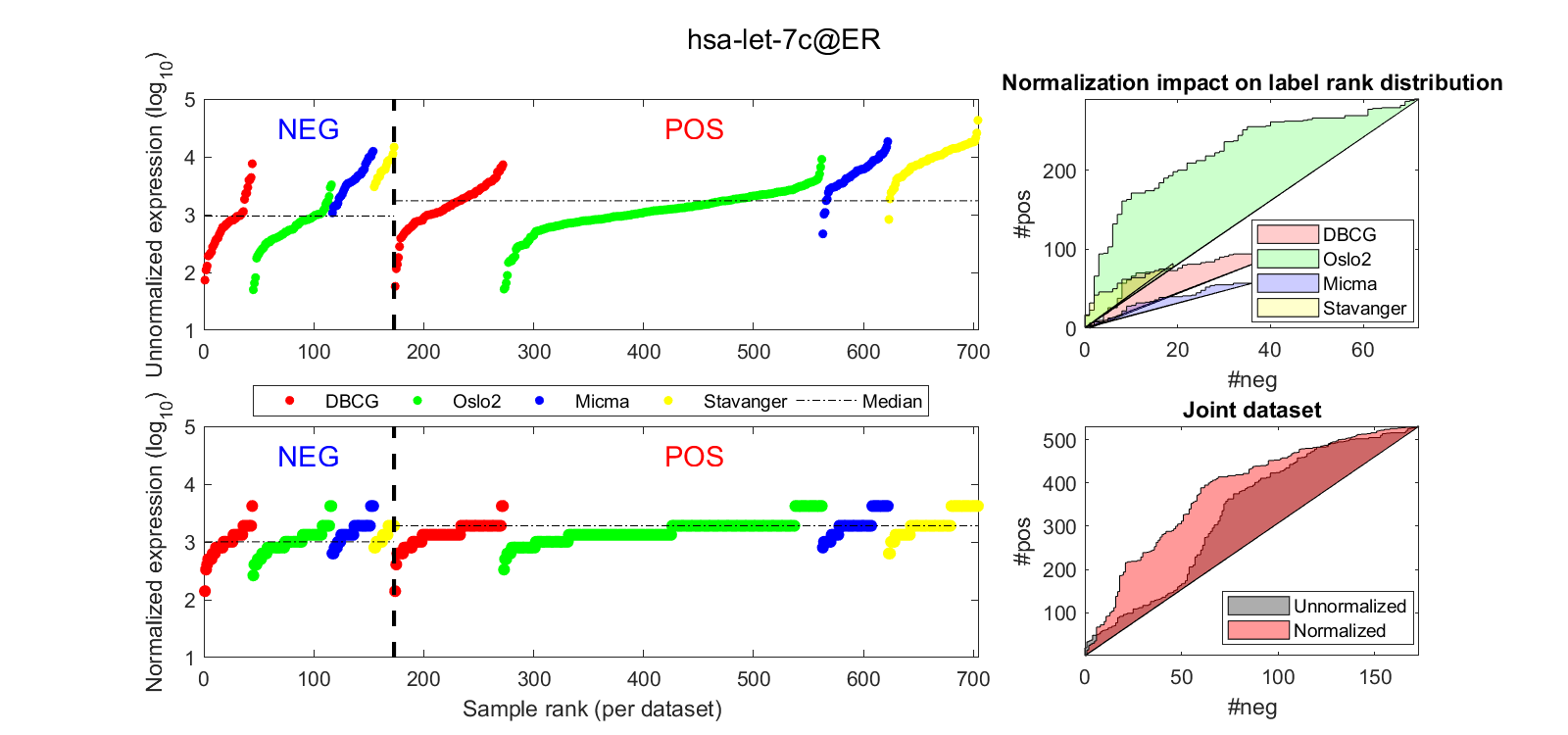

Supplement: S1 Data — Contains additional figures per miRNA pertaining to the analysis presented in Fig 2. (ZIP) [file pcbi.1008608.s001.zip › hsa-let-7c.png]

Array

50

100

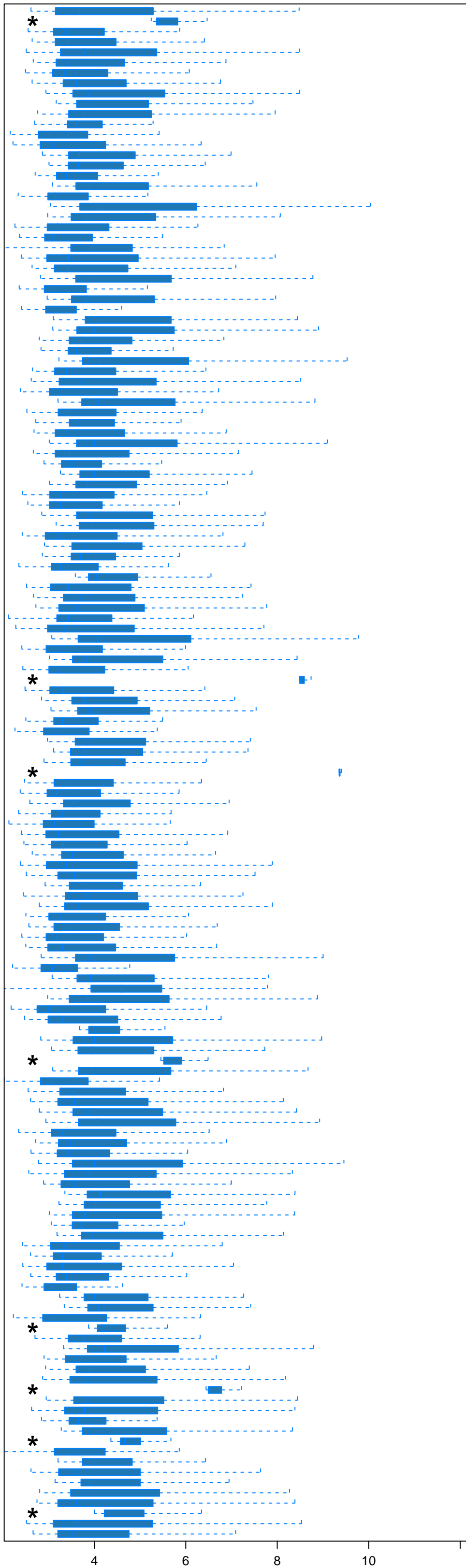

Supplement: S2 Data — Generated by the arrayQualityMetrics package, as described in “Dataset pre-processing and coverage”. Open index.html in either folder to view the detailed report data. (ZIP) [file pcbi.1008608.s002.zip › miRNA/Dbcg_QC_Report/box.pdf]

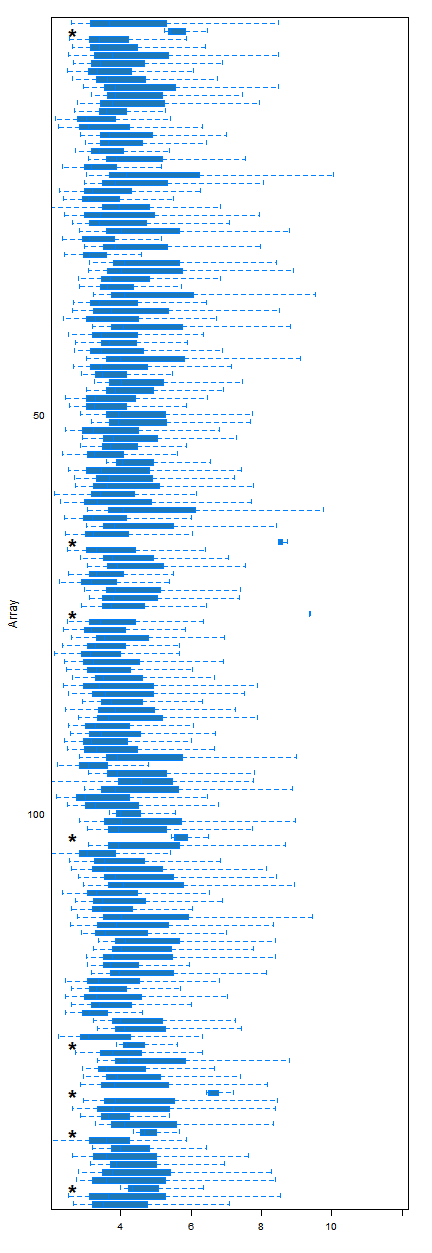

Supplement: S2 Data — Generated by the arrayQualityMetrics package, as described in “Dataset pre-processing and coverage”. Open index.html in either folder to view the detailed report data. (ZIP) [file pcbi.1008608.s002.zip › miRNA/Dbcg_QC_Report/box.png]

Density

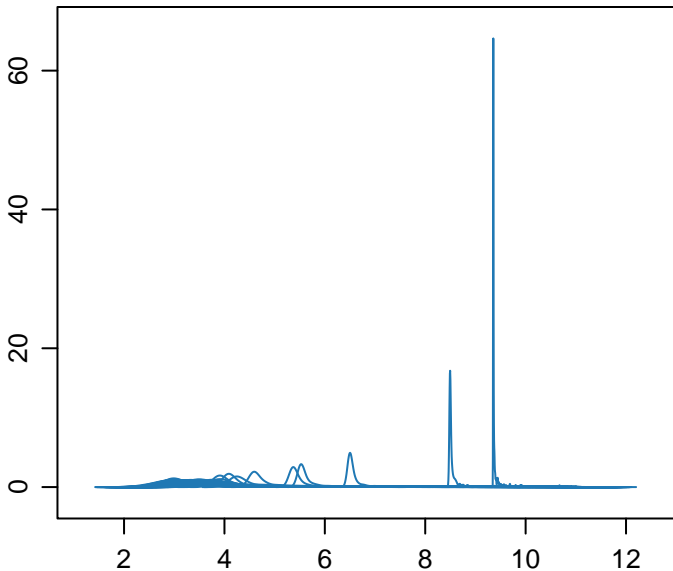

Supplement: S2 Data — Generated by the arrayQualityMetrics package, as described in “Dataset pre-processing and coverage”. Open index.html in either folder to view the detailed report data. (ZIP) [file pcbi.1008608.s002.zip › miRNA/Dbcg_QC_Report/dens.pdf]

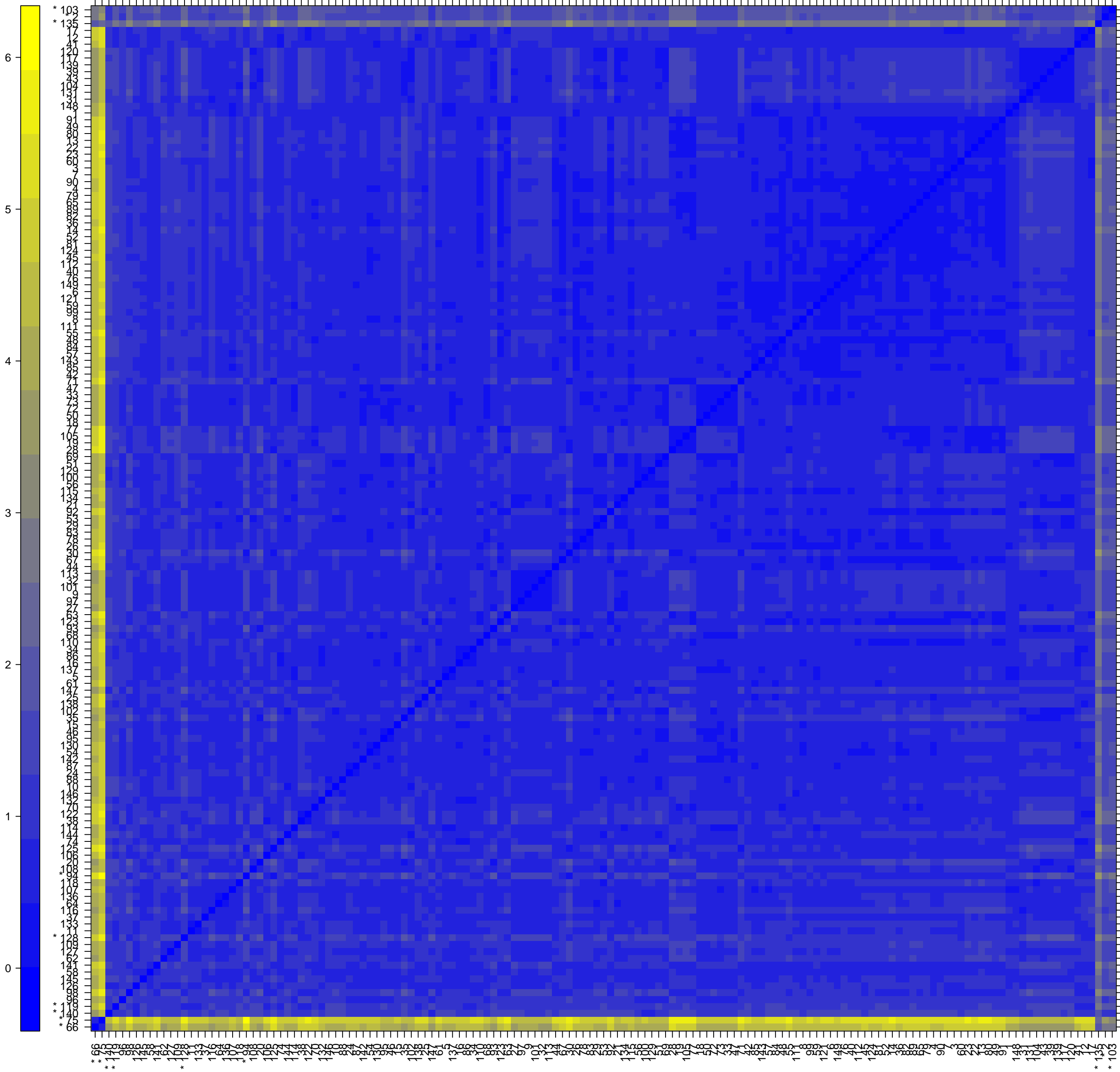

Supplement: S2 Data — Generated by the arrayQualityMetrics package, as described in “Dataset pre-processing and coverage”. Open index.html in either folder to view the detailed report data. (ZIP) [file pcbi.1008608.s002.zip › miRNA/Dbcg_QC_Report/hm.pdf]

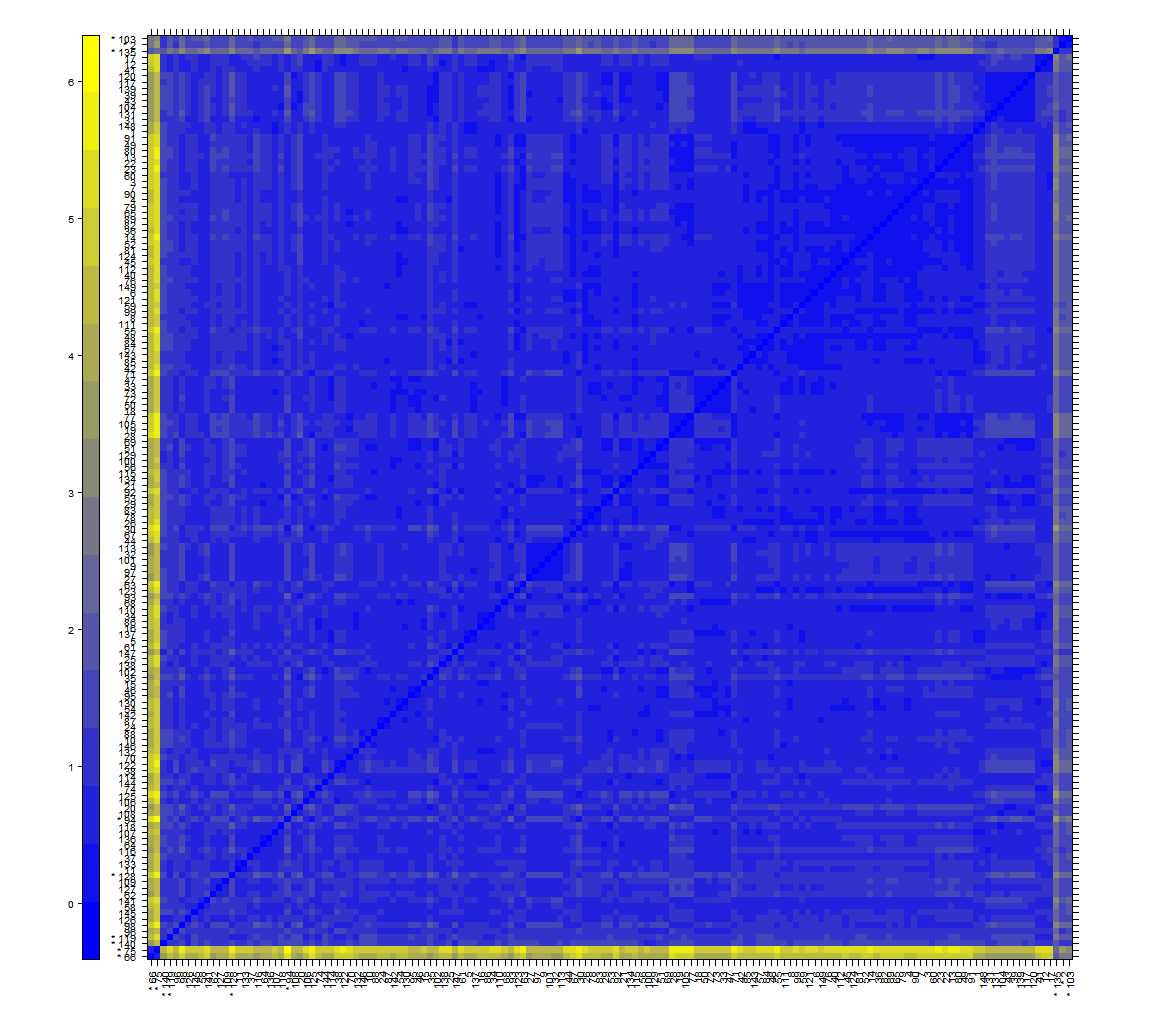

Supplement: S2 Data — Generated by the arrayQualityMetrics package, as described in “Dataset pre-processing and coverage”. Open index.html in either folder to view the detailed report data. (ZIP) [file pcbi.1008608.s002.zip › miRNA/Dbcg_QC_Report/hm.png]

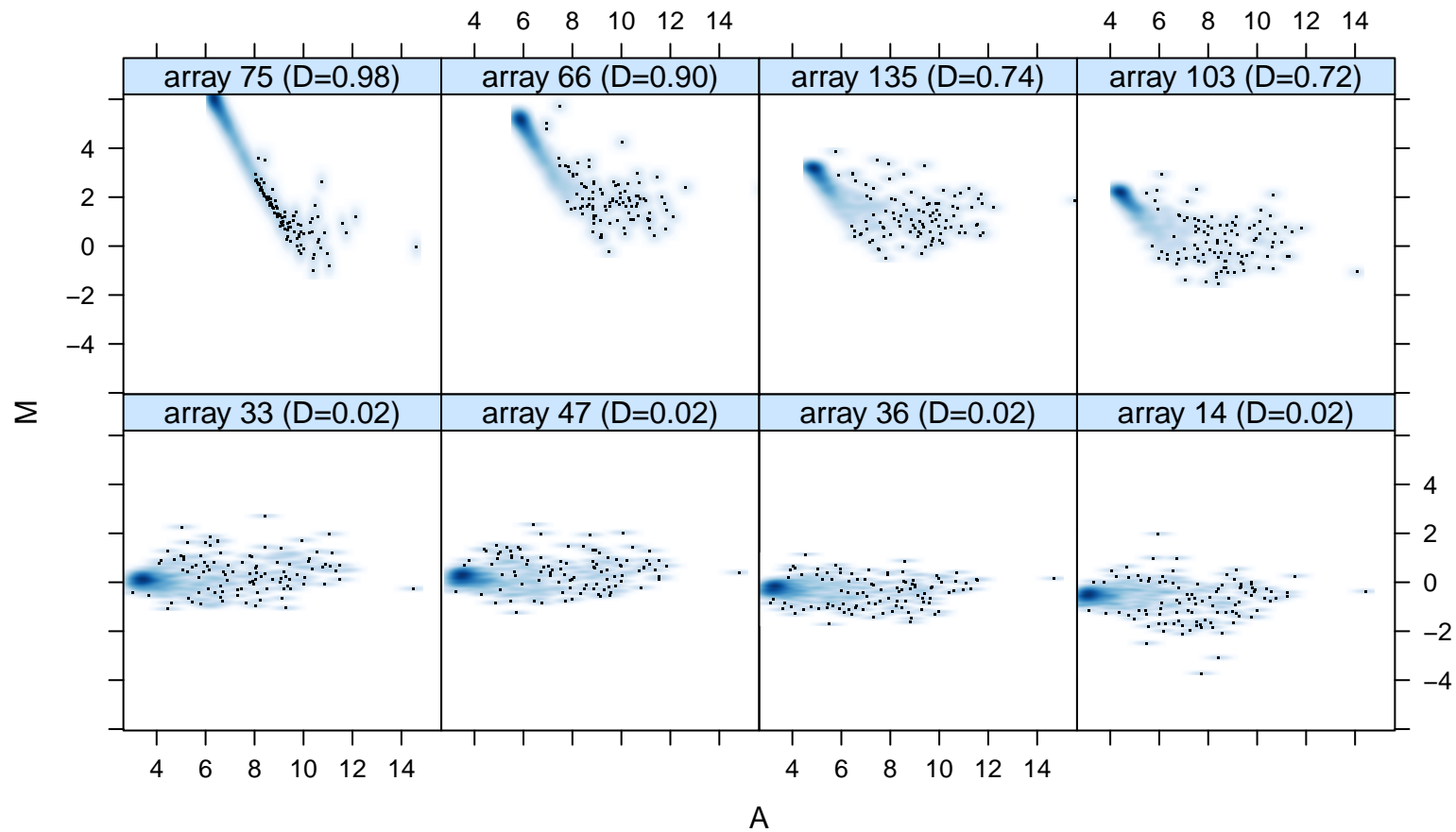

Supplement: S2 Data — Generated by the arrayQualityMetrics package, as described in “Dataset pre-processing and coverage”. Open index.html in either folder to view the detailed report data. (ZIP) [file pcbi.1008608.s002.zip › miRNA/Dbcg_QC_Report/ma.pdf]

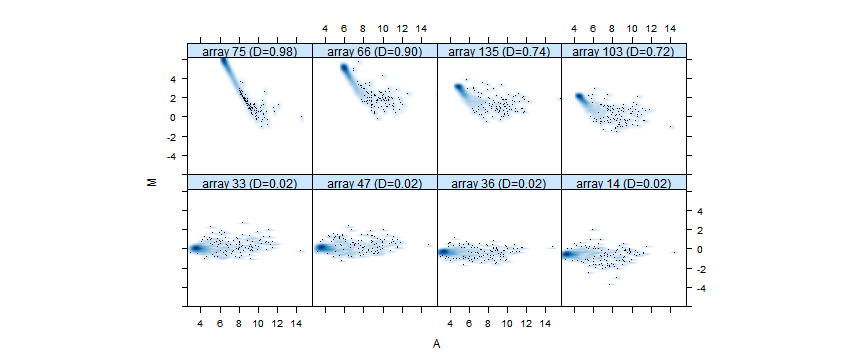

Supplement: S2 Data — Generated by the arrayQualityMetrics package, as described in “Dataset pre-processing and coverage”. Open index.html in either folder to view the detailed report data. (ZIP) [file pcbi.1008608.s002.zip › miRNA/Dbcg_QC_Report/ma.png]

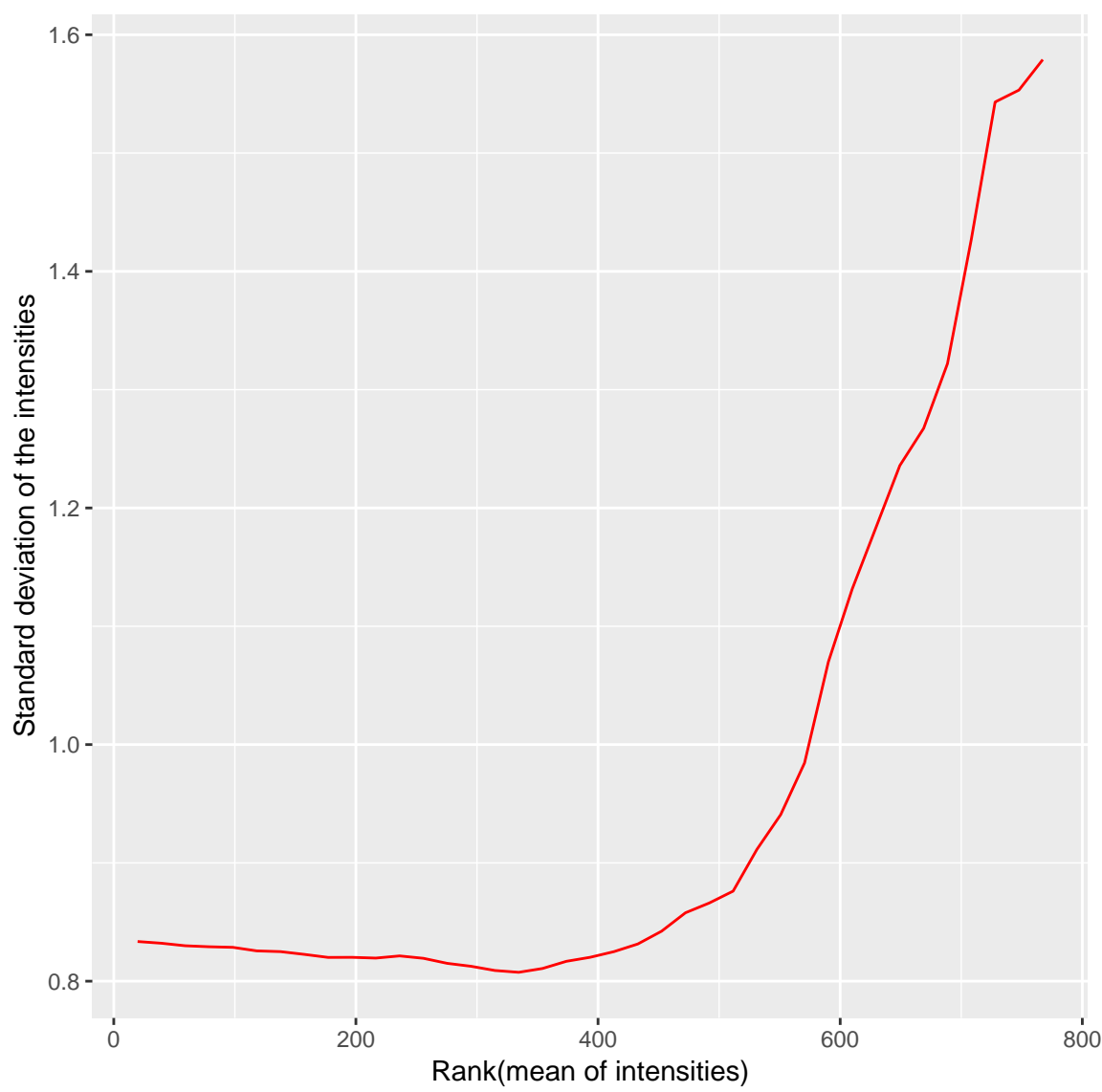

Supplement: S2 Data — Generated by the arrayQualityMetrics package, as described in “Dataset pre-processing and coverage”. Open index.html in either folder to view the detailed report data. (ZIP) [file pcbi.1008608.s002.zip › miRNA/Dbcg_QC_Report/msd.pdf]

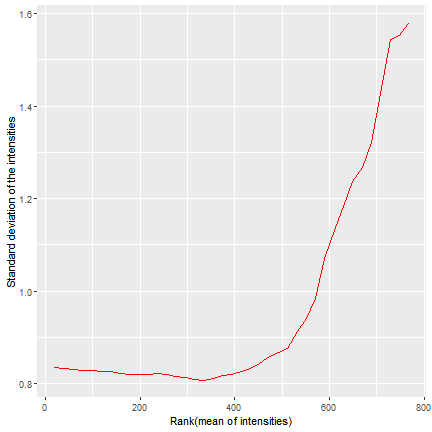

Supplement: S2 Data — Generated by the arrayQualityMetrics package, as described in “Dataset pre-processing and coverage”. Open index.html in either folder to view the detailed report data. (ZIP) [file pcbi.1008608.s002.zip › miRNA/Dbcg_QC_Report/msd.png]

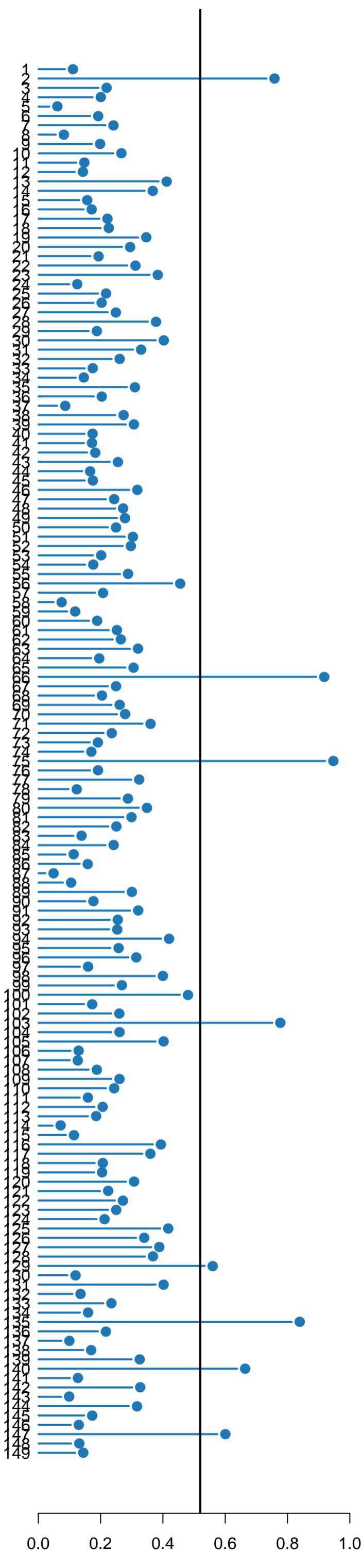

Supplement: S2 Data — Generated by the arrayQualityMetrics package, as described in “Dataset pre-processing and coverage”. Open index.html in either folder to view the detailed report data. (ZIP) [file pcbi.1008608.s002.zip › miRNA/Dbcg_QC_Report/out box.pdf]

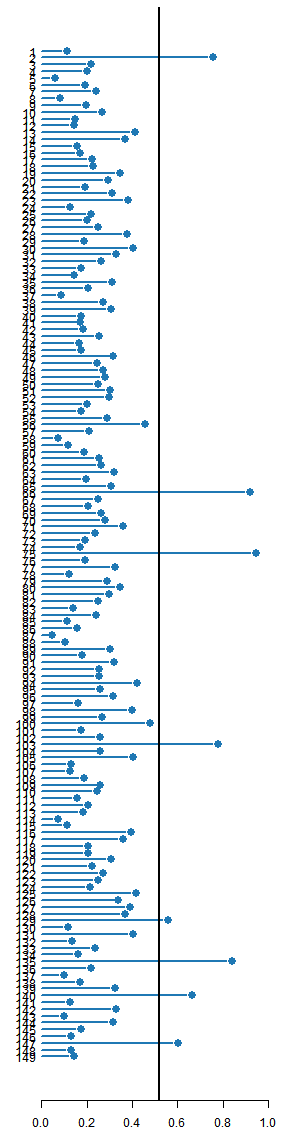

Supplement: S2 Data — Generated by the arrayQualityMetrics package, as described in “Dataset pre-processing and coverage”. Open index.html in either folder to view the detailed report data. (ZIP) [file pcbi.1008608.s002.zip › miRNA/Dbcg_QC_Report/out box.png]

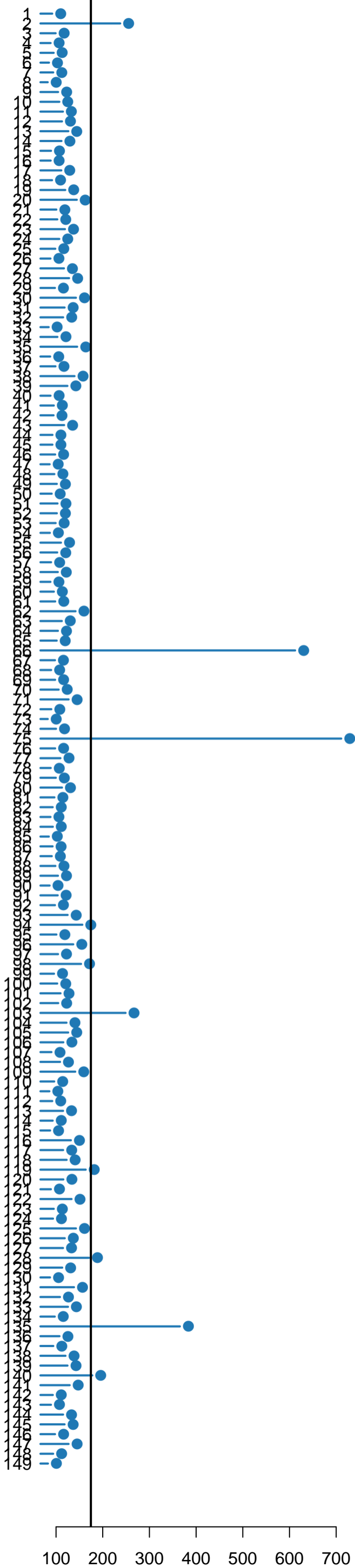

Supplement: S2 Data — Generated by the arrayQualityMetrics package, as described in “Dataset pre-processing and coverage”. Open index.html in either folder to view the detailed report data. (ZIP) [file pcbi.1008608.s002.zip › miRNA/Dbcg_QC_Report/out hm.pdf]

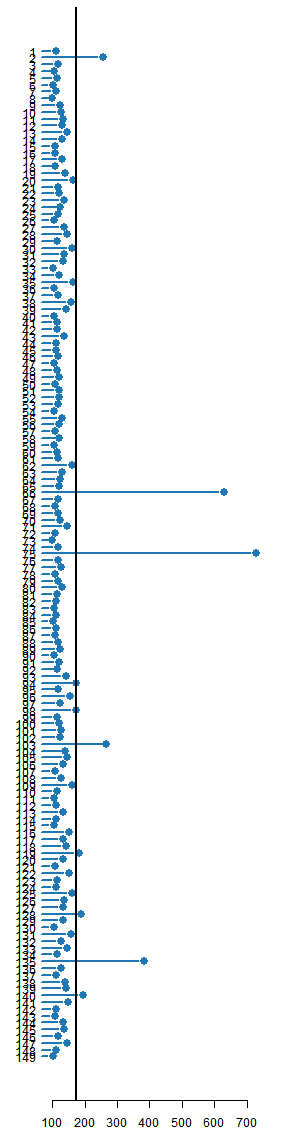

Supplement: S2 Data — Generated by the arrayQualityMetrics package, as described in “Dataset pre-processing and coverage”. Open index.html in either folder to view the detailed report data. (ZIP) [file pcbi.1008608.s002.zip › miRNA/Dbcg_QC_Report/out hm.png]

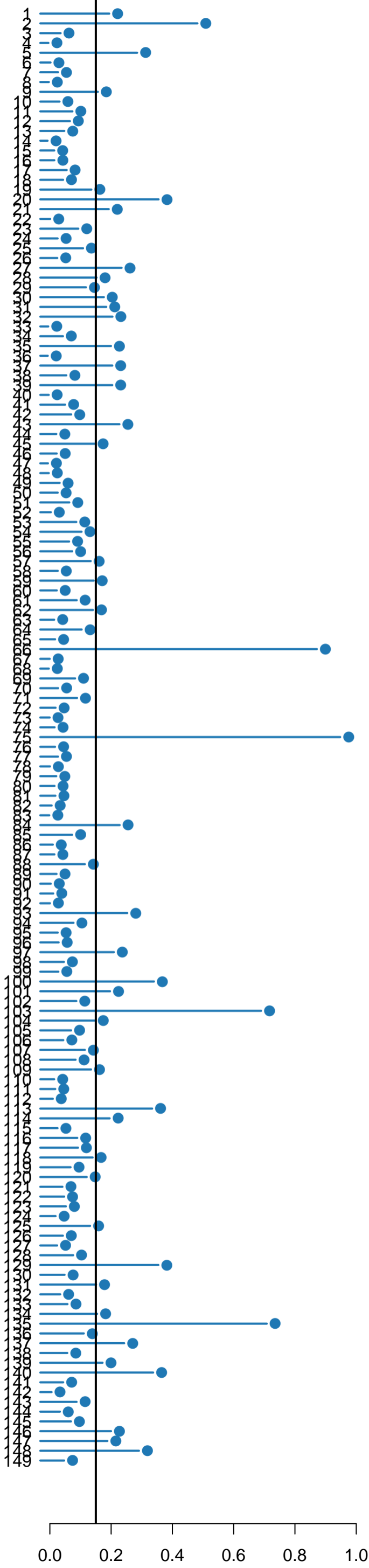

Supplement: S2 Data — Generated by the arrayQualityMetrics package, as described in “Dataset pre-processing and coverage”. Open index.html in either folder to view the detailed report data. (ZIP) [file pcbi.1008608.s002.zip › miRNA/Dbcg_QC_Report/out ma.pdf]

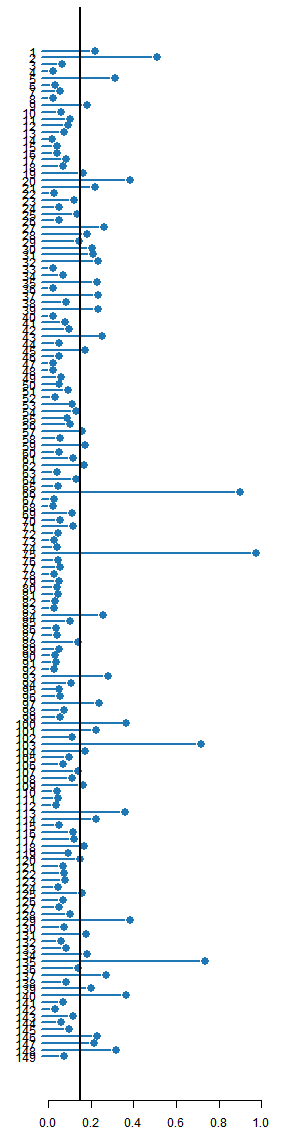

Supplement: S2 Data — Generated by the arrayQualityMetrics package, as described in “Dataset pre-processing and coverage”. Open index.html in either folder to view the detailed report data. (ZIP) [file pcbi.1008608.s002.zip › miRNA/Dbcg_QC_Report/out ma.png]

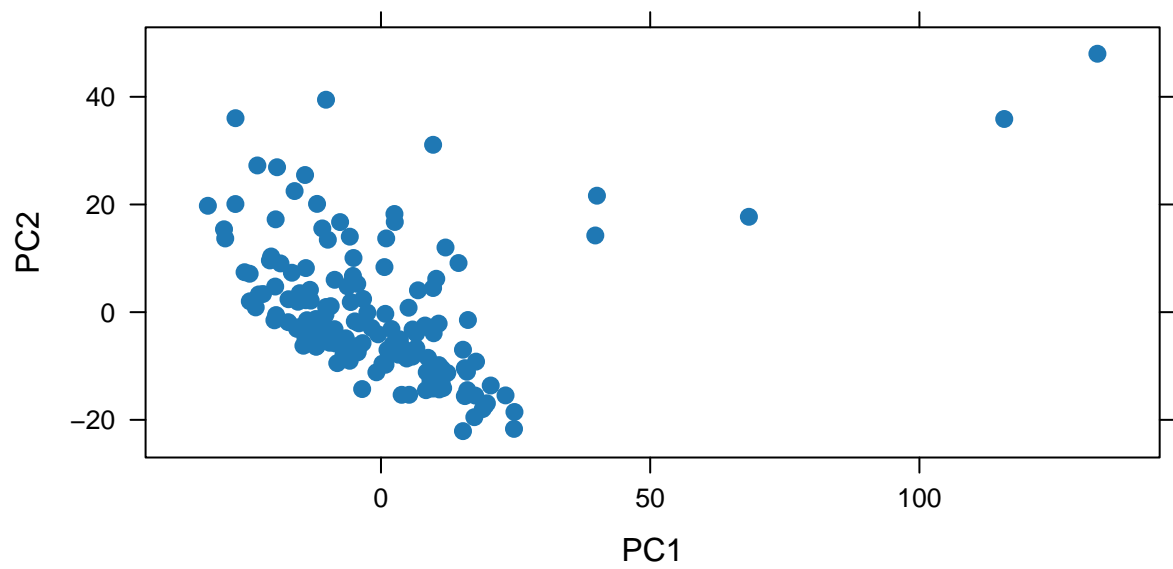

Supplement: S2 Data — Generated by the arrayQualityMetrics package, as described in “Dataset pre-processing and coverage”. Open index.html in either folder to view the detailed report data. (ZIP) [file pcbi.1008608.s002.zip › miRNA/Dbcg_QC_Report/pca.pdf]

Array

50

100

150

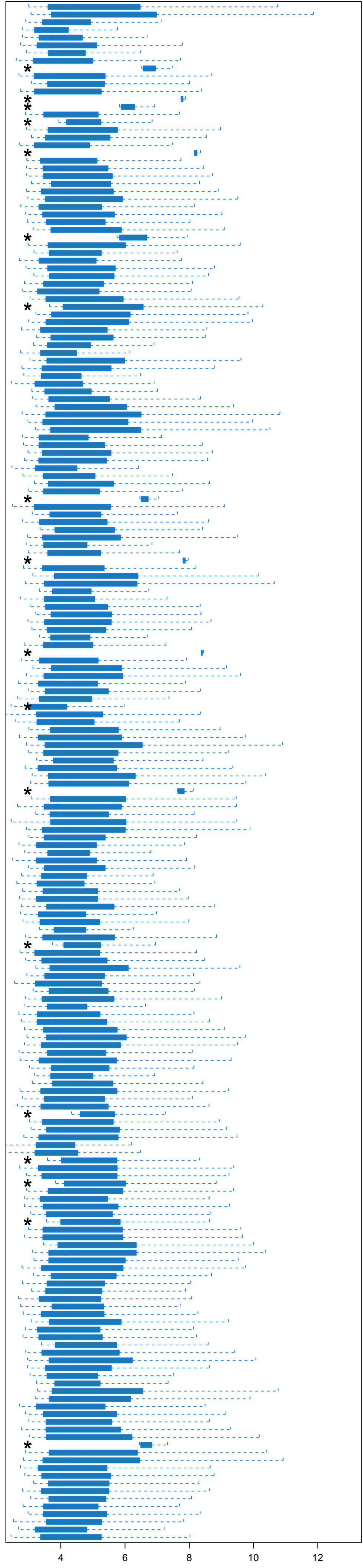

Supplement: S2 Data — Generated by the arrayQualityMetrics package, as described in “Dataset pre-processing and coverage”. Open index.html in either folder to view the detailed report data. (ZIP) [file pcbi.1008608.s002.zip › miRNA/Micma_QC_Report/box.pdf]

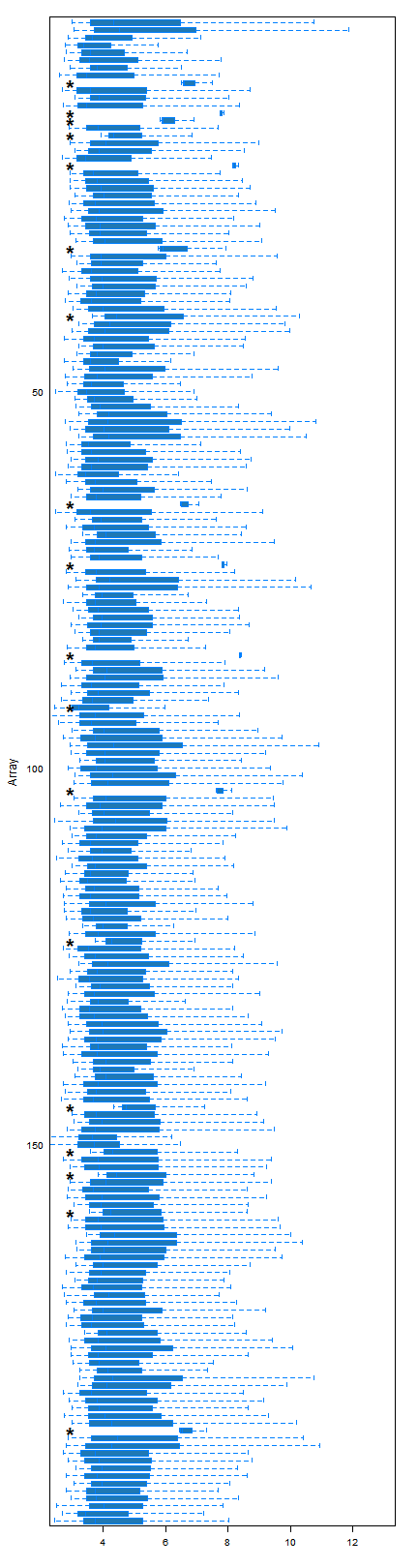

Supplement: S2 Data — Generated by the arrayQualityMetrics package, as described in “Dataset pre-processing and coverage”. Open index.html in either folder to view the detailed report data. (ZIP) [file pcbi.1008608.s002.zip › miRNA/Micma_QC_Report/box.png]

Density

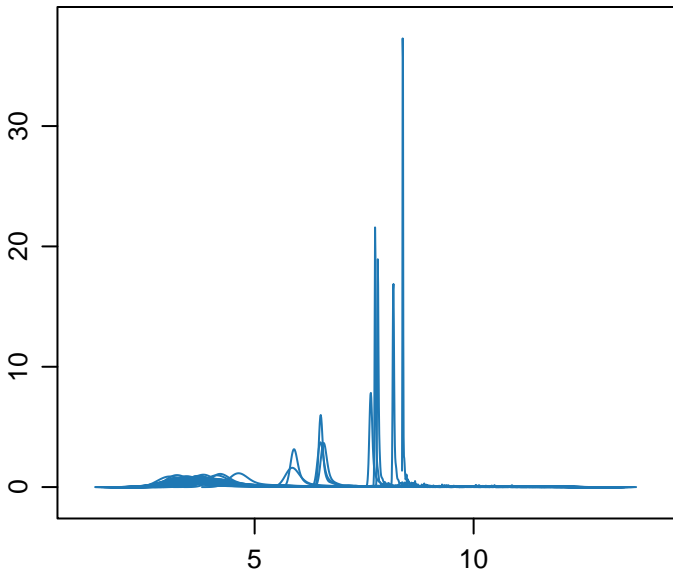

Supplement: S2 Data — Generated by the arrayQualityMetrics package, as described in “Dataset pre-processing and coverage”. Open index.html in either folder to view the detailed report data. (ZIP) [file pcbi.1008608.s002.zip › miRNA/Micma_QC_Report/dens.pdf]

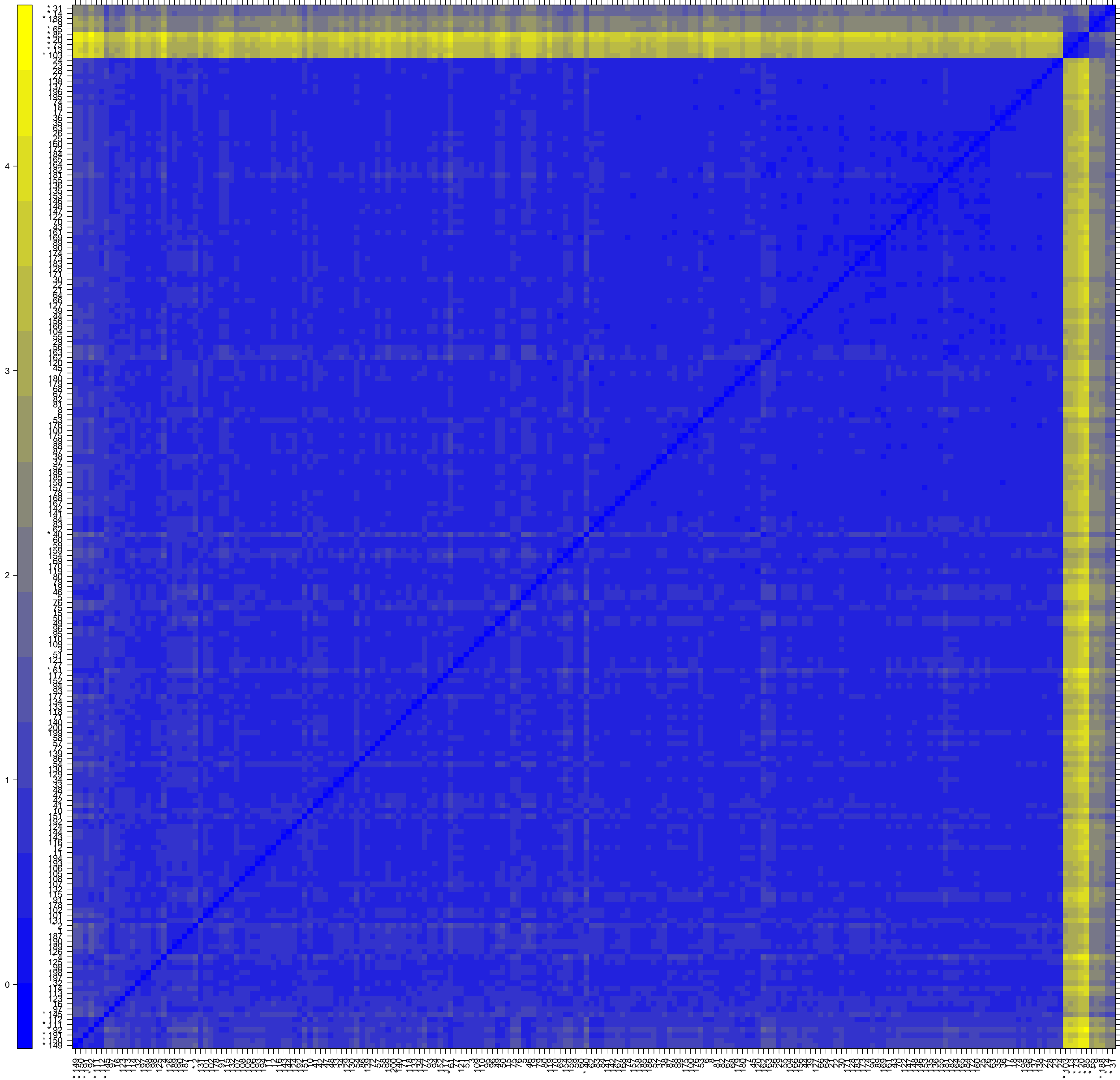

Supplement: S2 Data — Generated by the arrayQualityMetrics package, as described in “Dataset pre-processing and coverage”. Open index.html in either folder to view the detailed report data. (ZIP) [file pcbi.1008608.s002.zip › miRNA/Micma_QC_Report/hm.pdf]

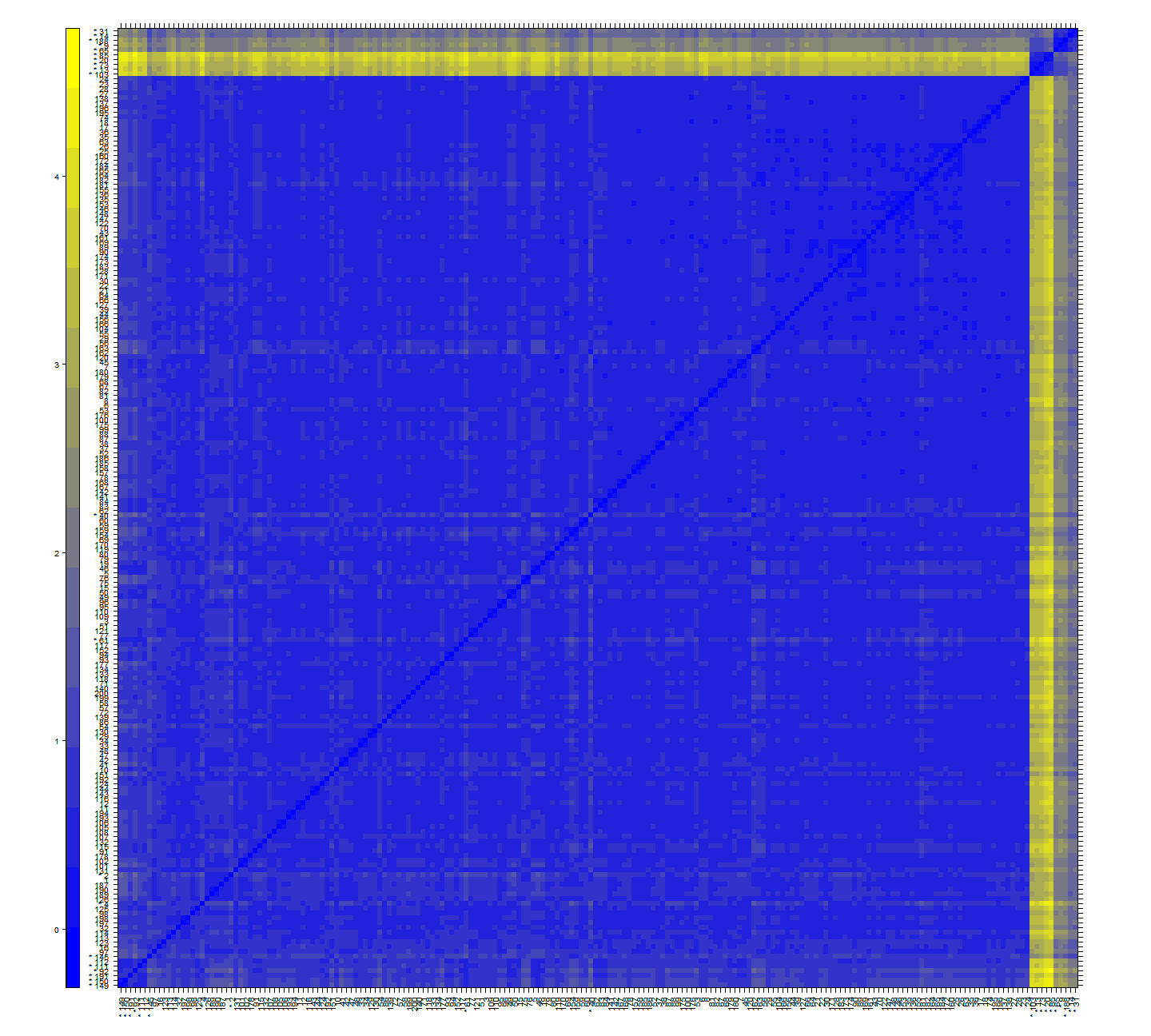

Supplement: S2 Data — Generated by the arrayQualityMetrics package, as described in “Dataset pre-processing and coverage”. Open index.html in either folder to view the detailed report data. (ZIP) [file pcbi.1008608.s002.zip › miRNA/Micma_QC_Report/hm.png]

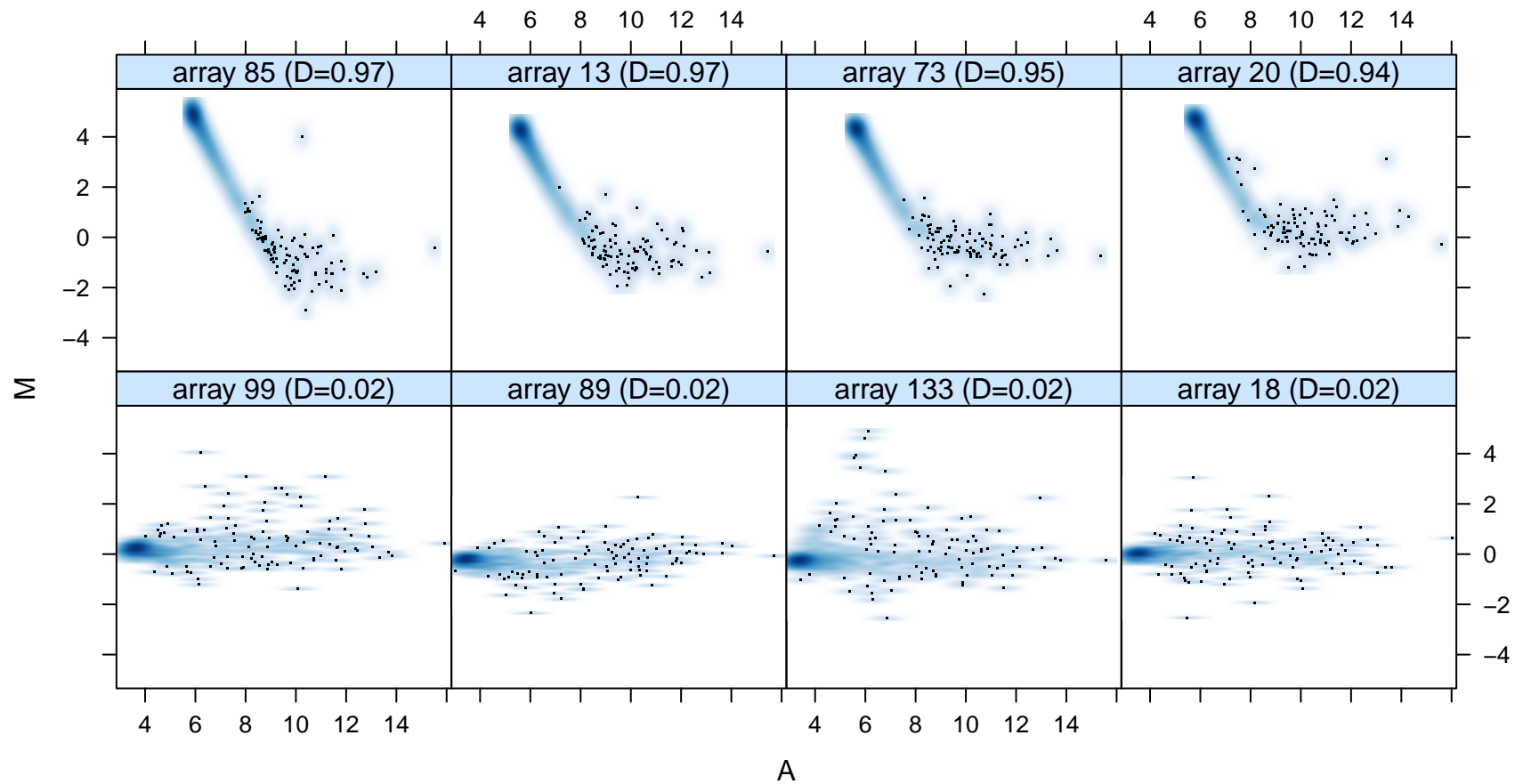

Supplement: S2 Data — Generated by the arrayQualityMetrics package, as described in “Dataset pre-processing and coverage”. Open index.html in either folder to view the detailed report data. (ZIP) [file pcbi.1008608.s002.zip › miRNA/Micma_QC_Report/ma.pdf]

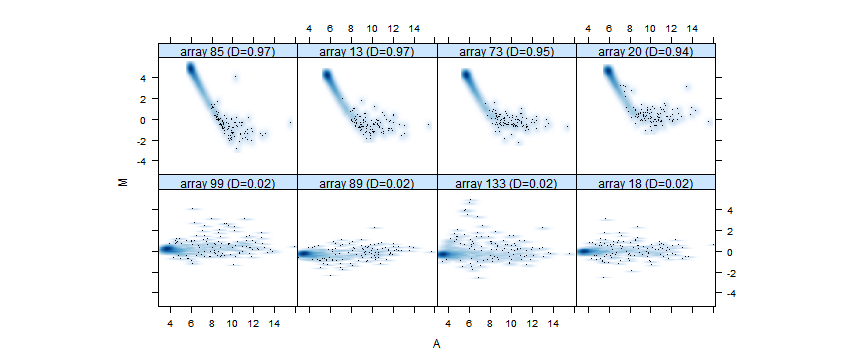

Supplement: S2 Data — Generated by the arrayQualityMetrics package, as described in “Dataset pre-processing and coverage”. Open index.html in either folder to view the detailed report data. (ZIP) [file pcbi.1008608.s002.zip › miRNA/Micma_QC_Report/ma.png]

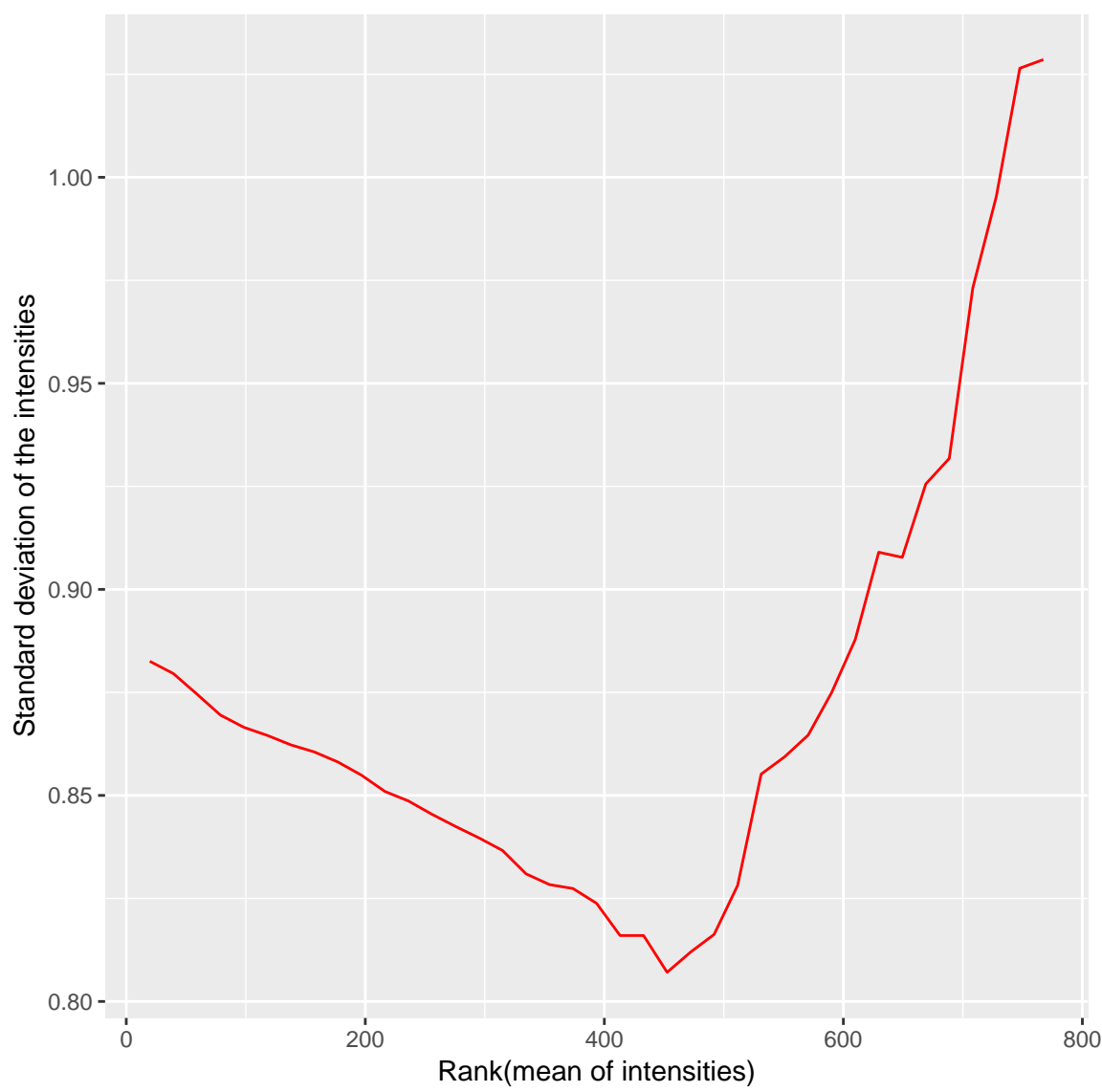

Supplement: S2 Data — Generated by the arrayQualityMetrics package, as described in “Dataset pre-processing and coverage”. Open index.html in either folder to view the detailed report data. (ZIP) [file pcbi.1008608.s002.zip › miRNA/Micma_QC_Report/msd.pdf]

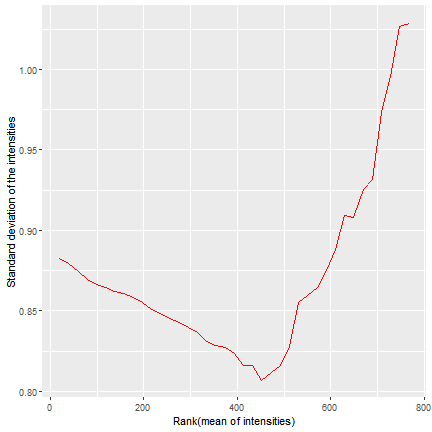

Supplement: S2 Data — Generated by the arrayQualityMetrics package, as described in “Dataset pre-processing and coverage”. Open index.html in either folder to view the detailed report data. (ZIP) [file pcbi.1008608.s002.zip › miRNA/Micma_QC_Report/msd.png]

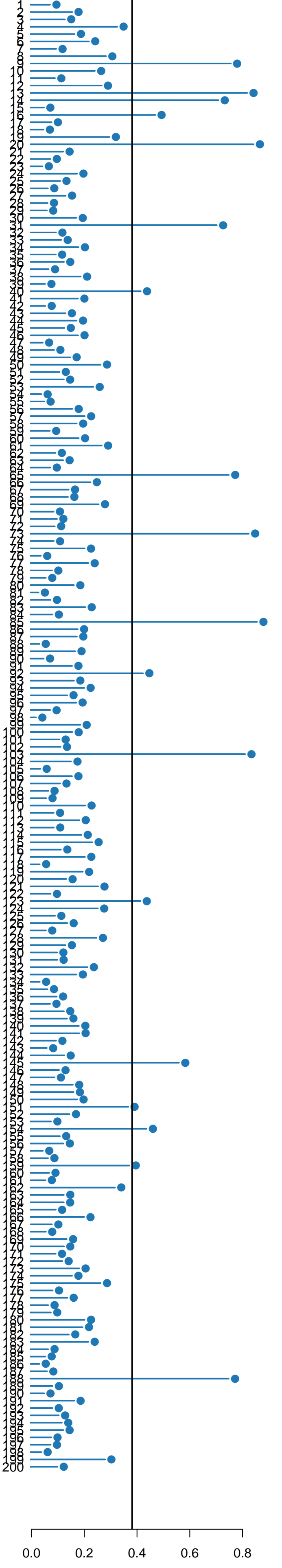

Supplement: S2 Data — Generated by the arrayQualityMetrics package, as described in “Dataset pre-processing and coverage”. Open index.html in either folder to view the detailed report data. (ZIP) [file pcbi.1008608.s002.zip › miRNA/Micma_QC_Report/out box.pdf]

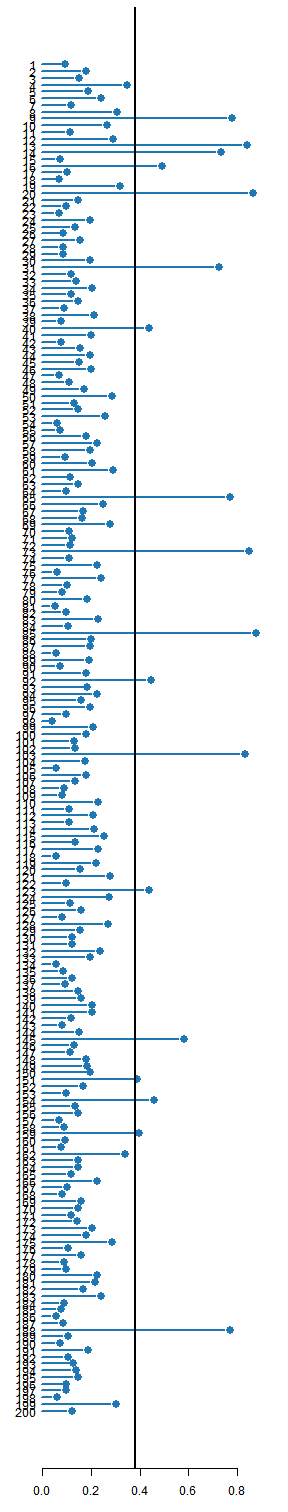

Supplement: S2 Data — Generated by the arrayQualityMetrics package, as described in “Dataset pre-processing and coverage”. Open index.html in either folder to view the detailed report data. (ZIP) [file pcbi.1008608.s002.zip › miRNA/Micma_QC_Report/out box.png]

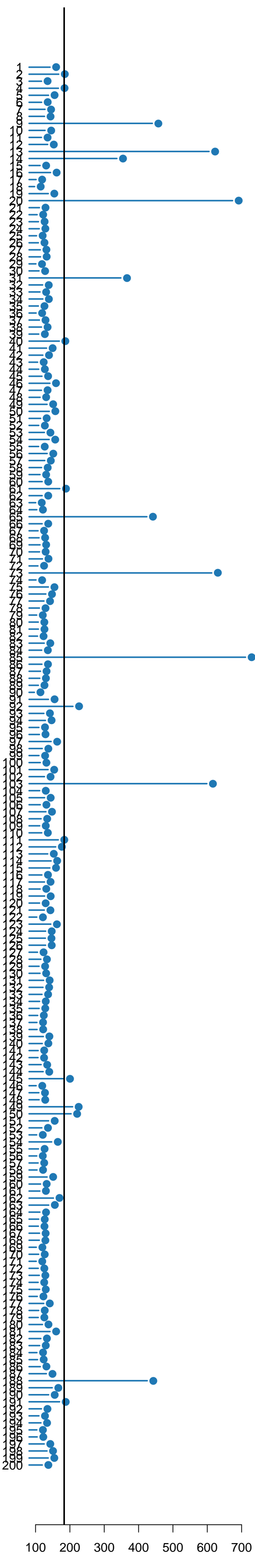

Supplement: S2 Data — Generated by the arrayQualityMetrics package, as described in “Dataset pre-processing and coverage”. Open index.html in either folder to view the detailed report data. (ZIP) [file pcbi.1008608.s002.zip › miRNA/Micma_QC_Report/out hm.pdf]

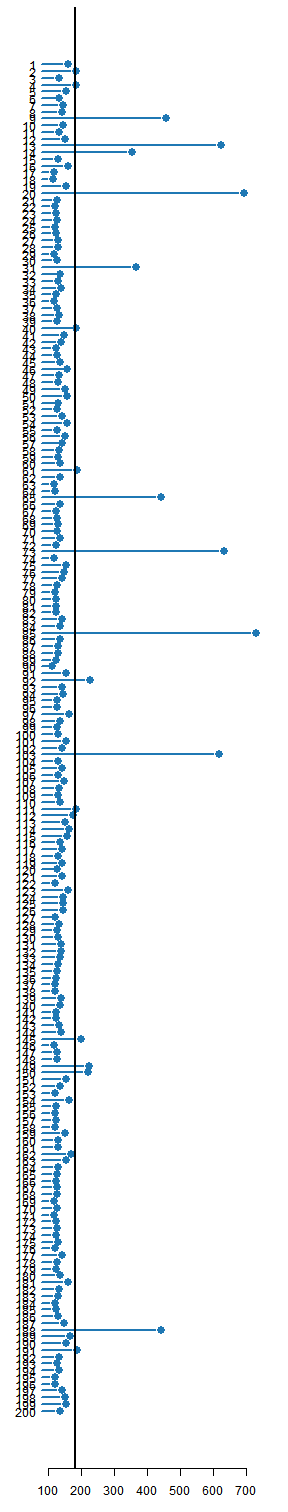

Supplement: S2 Data — Generated by the arrayQualityMetrics package, as described in “Dataset pre-processing and coverage”. Open index.html in either folder to view the detailed report data. (ZIP) [file pcbi.1008608.s002.zip › miRNA/Micma_QC_Report/out hm.png]

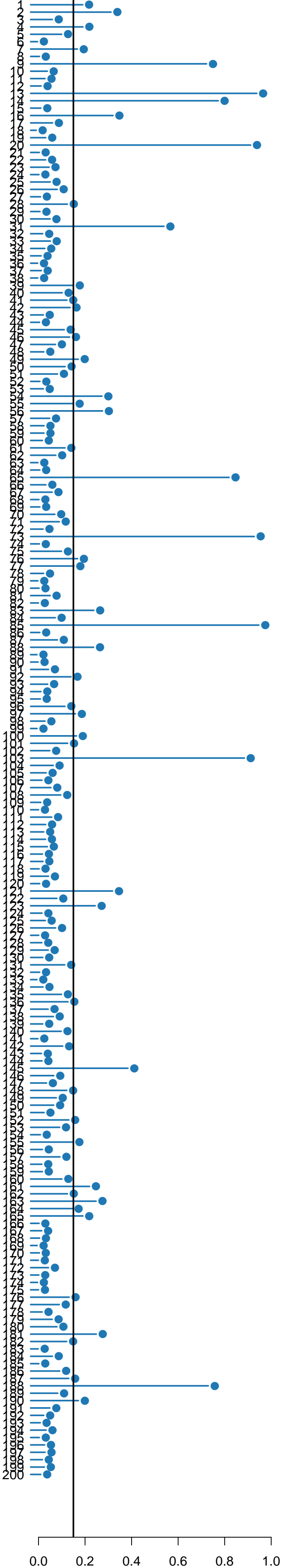

Supplement: S2 Data — Generated by the arrayQualityMetrics package, as described in “Dataset pre-processing and coverage”. Open index.html in either folder to view the detailed report data. (ZIP) [file pcbi.1008608.s002.zip › miRNA/Micma_QC_Report/out ma.pdf]

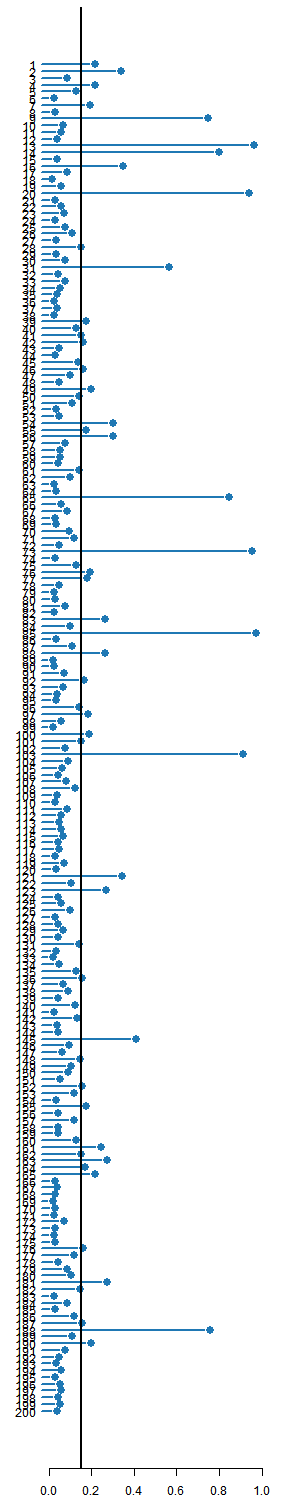

Supplement: S2 Data — Generated by the arrayQualityMetrics package, as described in “Dataset pre-processing and coverage”. Open index.html in either folder to view the detailed report data. (ZIP) [file pcbi.1008608.s002.zip › miRNA/Micma_QC_Report/out ma.png]

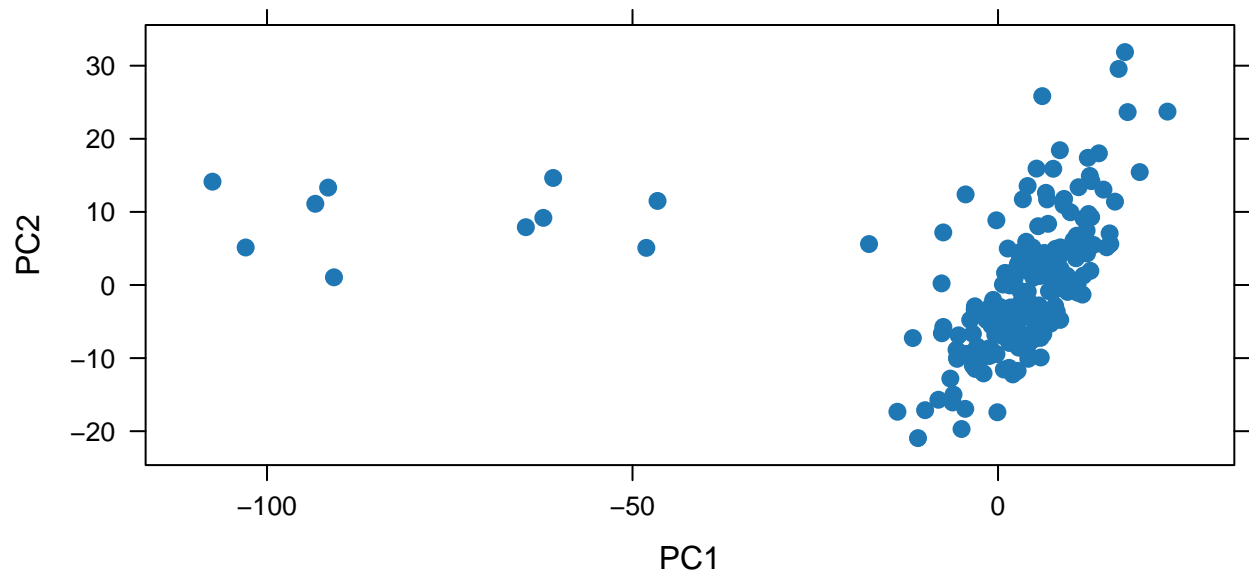

Supplement: S2 Data — Generated by the arrayQualityMetrics package, as described in “Dataset pre-processing and coverage”. Open index.html in either folder to view the detailed report data. (ZIP) [file pcbi.1008608.s002.zip › miRNA/Micma_QC_Report/pca.pdf]

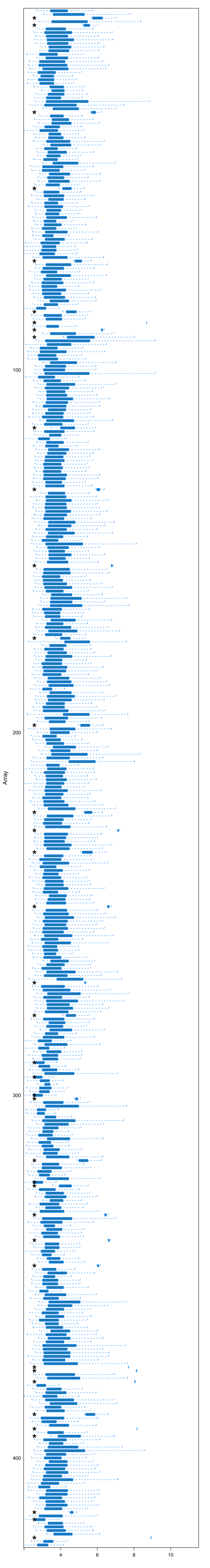

Supplement: S2 Data — Generated by the arrayQualityMetrics package, as described in “Dataset pre-processing and coverage”. Open index.html in either folder to view the detailed report data. (ZIP) [file pcbi.1008608.s002.zip › miRNA/Oslo2_QC_Report/box.pdf]

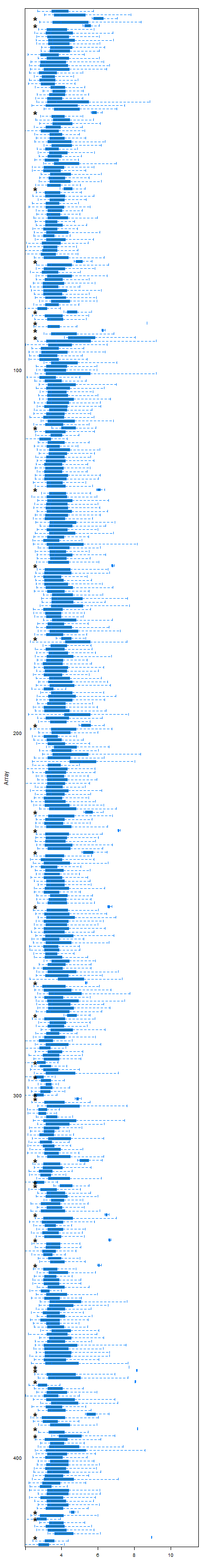

Supplement: S2 Data — Generated by the arrayQualityMetrics package, as described in “Dataset pre-processing and coverage”. Open index.html in either folder to view the detailed report data. (ZIP) [file pcbi.1008608.s002.zip › miRNA/Oslo2_QC_Report/box.png]

Density

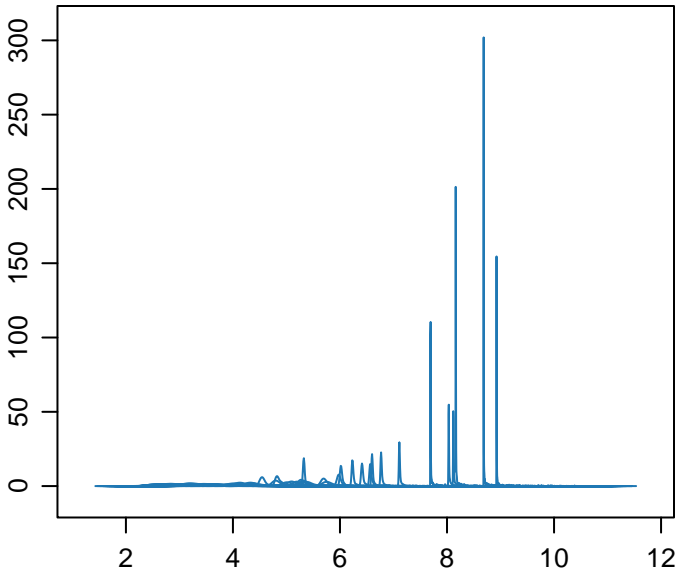

Supplement: S2 Data — Generated by the arrayQualityMetrics package, as described in “Dataset pre-processing and coverage”. Open index.html in either folder to view the detailed report data. (ZIP) [file pcbi.1008608.s002.zip › miRNA/Oslo2_QC_Report/dens.pdf]

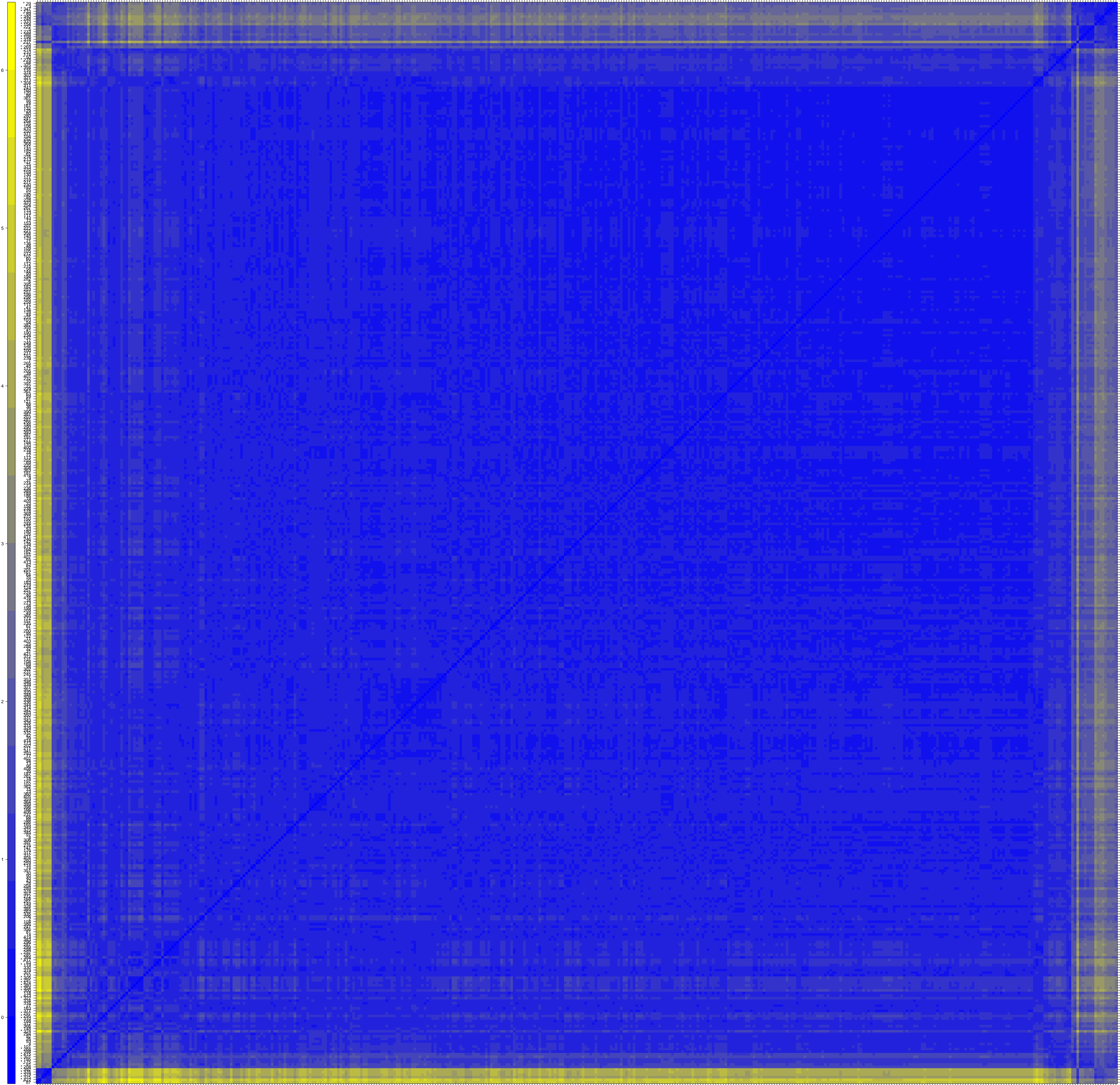

Supplement: S2 Data — Generated by the arrayQualityMetrics package, as described in “Dataset pre-processing and coverage”. Open index.html in either folder to view the detailed report data. (ZIP) [file pcbi.1008608.s002.zip › miRNA/Oslo2_QC_Report/hm.pdf]

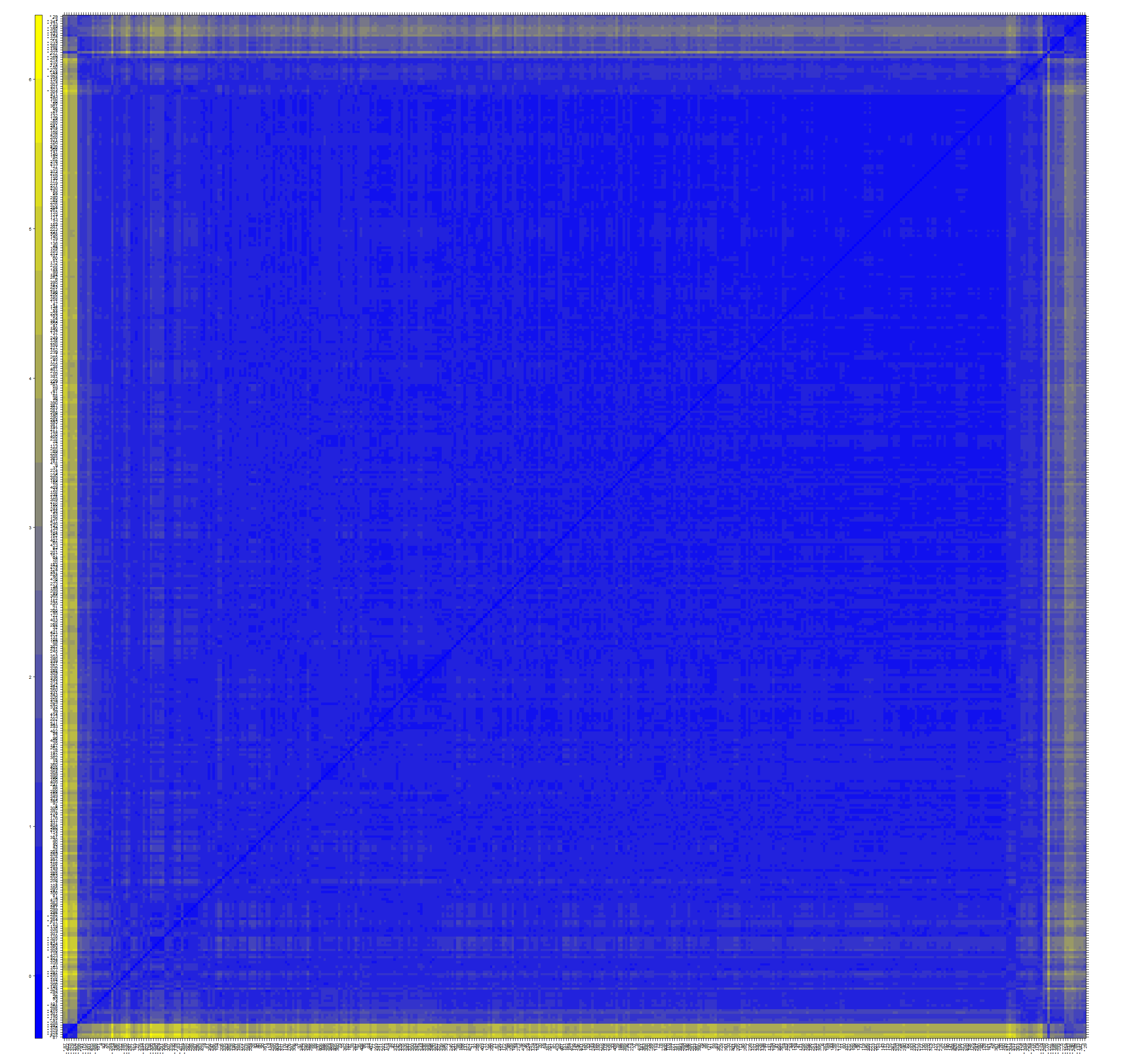

Supplement: S2 Data — Generated by the arrayQualityMetrics package, as described in “Dataset pre-processing and coverage”. Open index.html in either folder to view the detailed report data. (ZIP) [file pcbi.1008608.s002.zip › miRNA/Oslo2_QC_Report/hm.png]

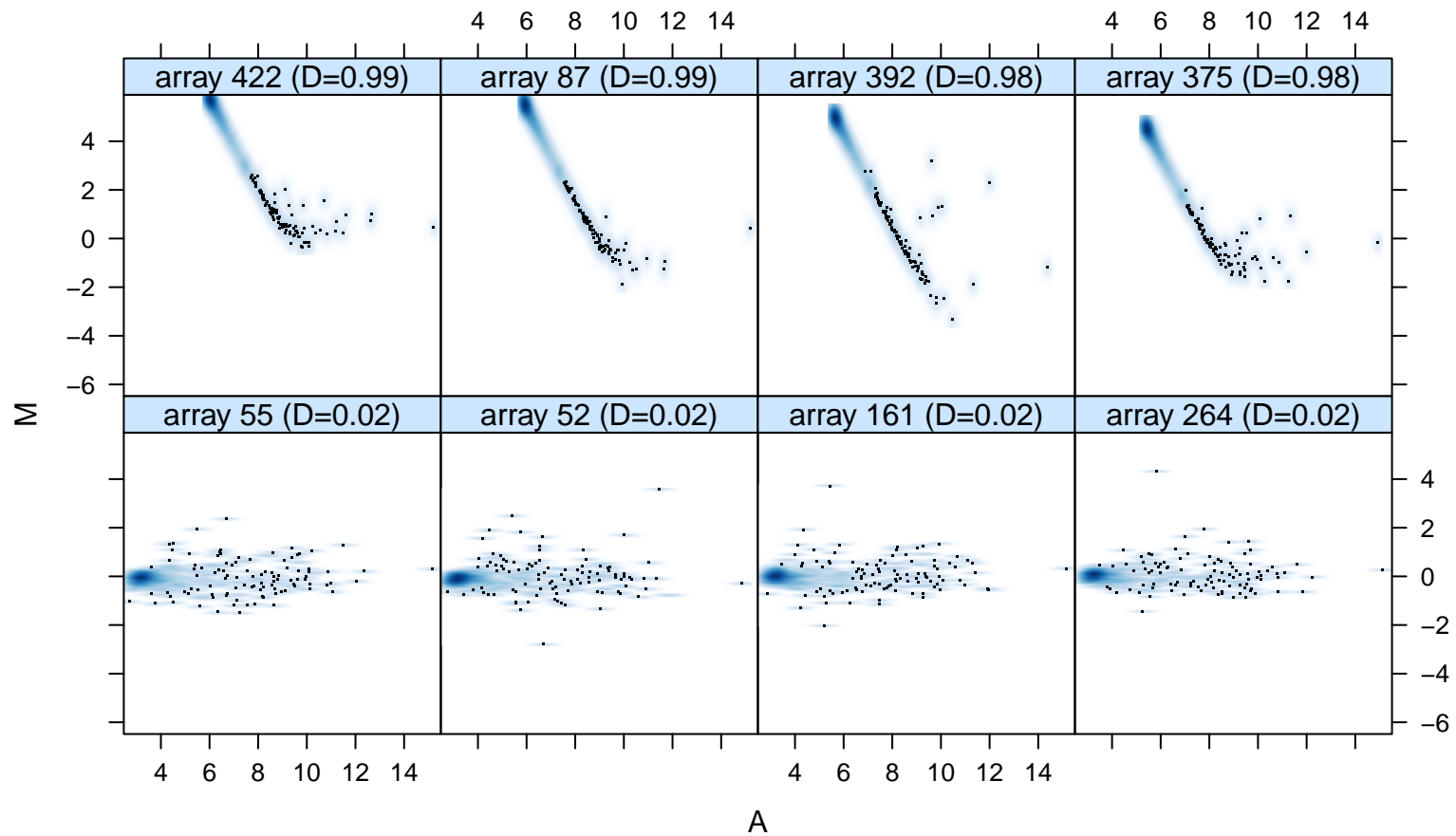

Supplement: S2 Data — Generated by the arrayQualityMetrics package, as described in “Dataset pre-processing and coverage”. Open index.html in either folder to view the detailed report data. (ZIP) [file pcbi.1008608.s002.zip › miRNA/Oslo2_QC_Report/ma.pdf]

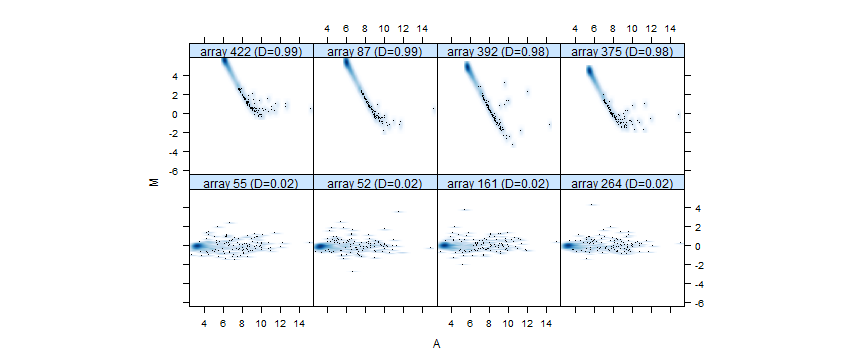

Supplement: S2 Data — Generated by the arrayQualityMetrics package, as described in “Dataset pre-processing and coverage”. Open index.html in either folder to view the detailed report data. (ZIP) [file pcbi.1008608.s002.zip › miRNA/Oslo2_QC_Report/ma.png]

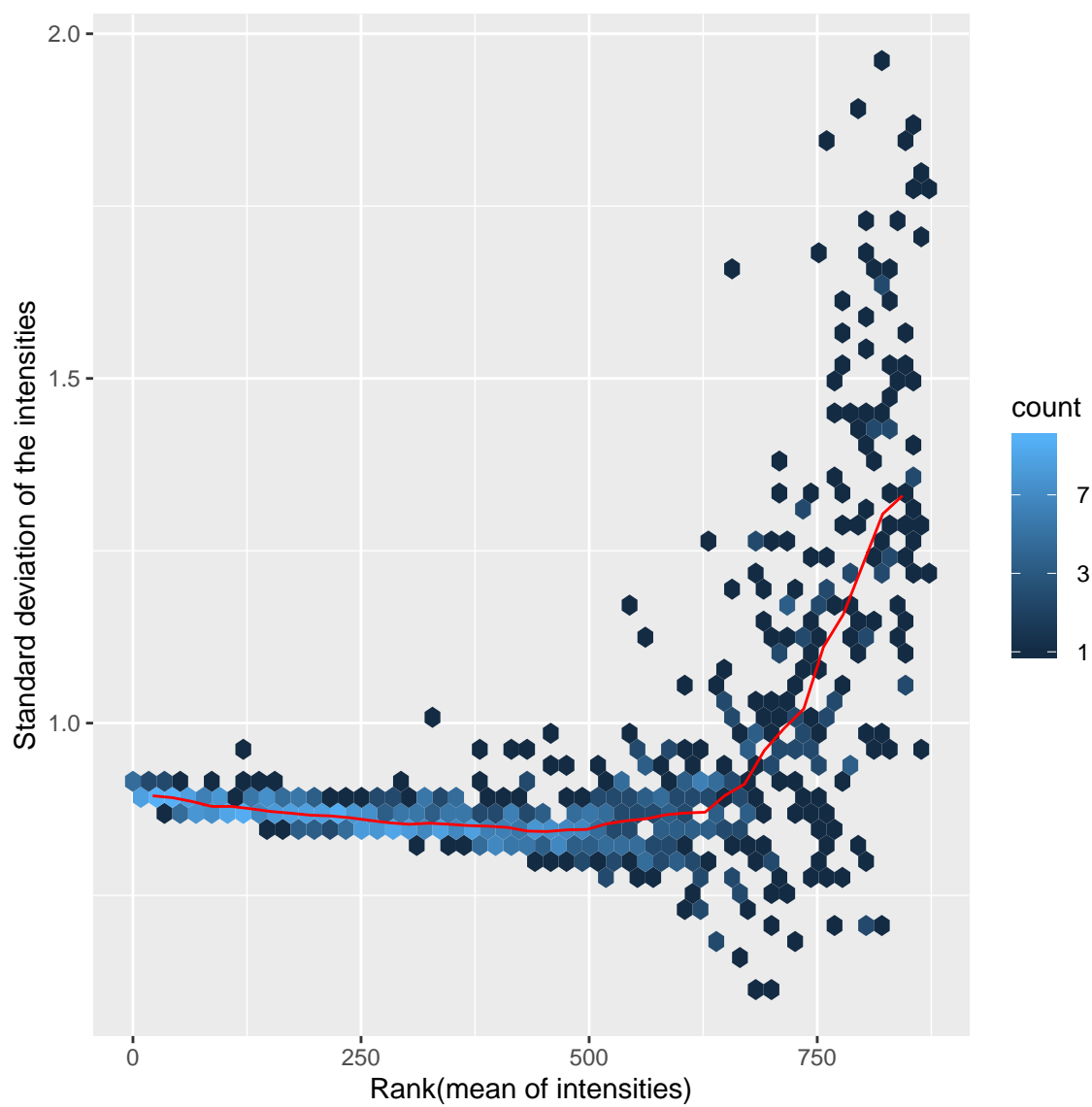

Supplement: S2 Data — Generated by the arrayQualityMetrics package, as described in “Dataset pre-processing and coverage”. Open index.html in either folder to view the detailed report data. (ZIP) [file pcbi.1008608.s002.zip › miRNA/Oslo2_QC_Report/msd.pdf]

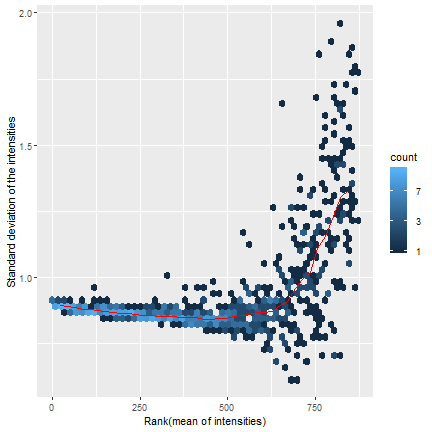

Supplement: S2 Data — Generated by the arrayQualityMetrics package, as described in “Dataset pre-processing and coverage”. Open index.html in either folder to view the detailed report data. (ZIP) [file pcbi.1008608.s002.zip › miRNA/Oslo2_QC_Report/msd.png]

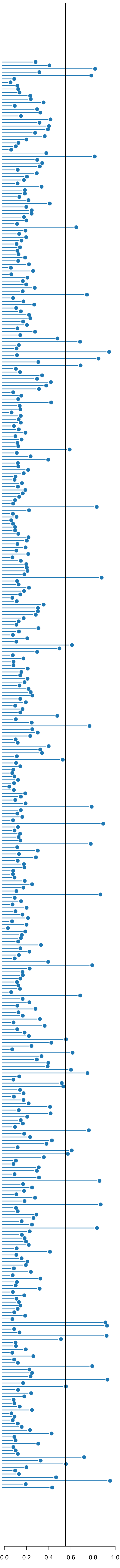

Supplement: S2 Data — Generated by the arrayQualityMetrics package, as described in “Dataset pre-processing and coverage”. Open index.html in either folder to view the detailed report data. (ZIP) [file pcbi.1008608.s002.zip › miRNA/Oslo2_QC_Report/out box.pdf]

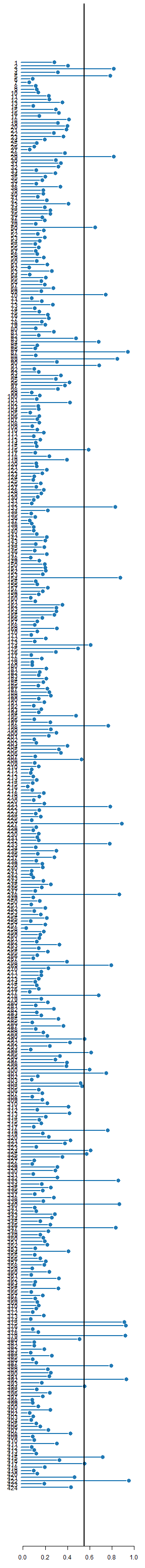

Supplement: S2 Data — Generated by the arrayQualityMetrics package, as described in “Dataset pre-processing and coverage”. Open index.html in either folder to view the detailed report data. (ZIP) [file pcbi.1008608.s002.zip › miRNA/Oslo2_QC_Report/out box.png]

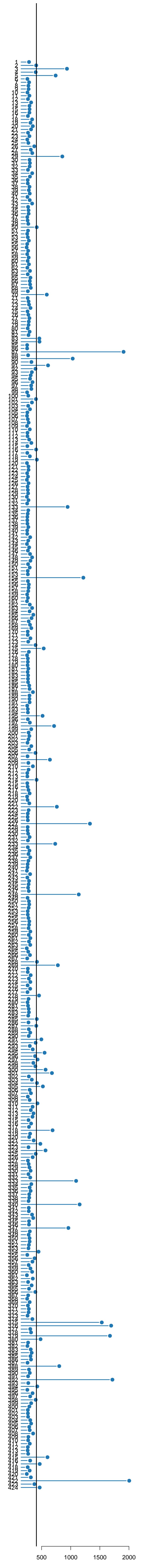

Supplement: S2 Data — Generated by the arrayQualityMetrics package, as described in “Dataset pre-processing and coverage”. Open index.html in either folder to view the detailed report data. (ZIP) [file pcbi.1008608.s002.zip › miRNA/Oslo2_QC_Report/out hm.pdf]

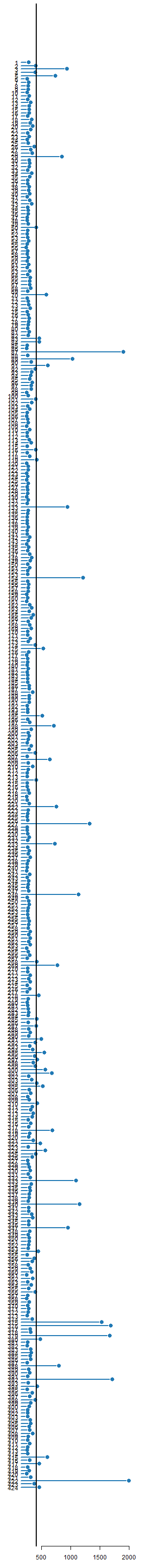

Supplement: S2 Data — Generated by the arrayQualityMetrics package, as described in “Dataset pre-processing and coverage”. Open index.html in either folder to view the detailed report data. (ZIP) [file pcbi.1008608.s002.zip › miRNA/Oslo2_QC_Report/out hm.png]

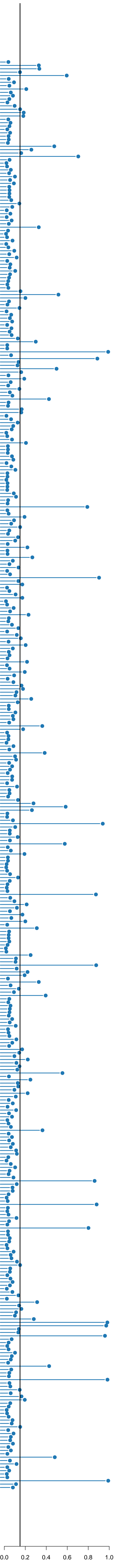

Supplement: S2 Data — Generated by the arrayQualityMetrics package, as described in “Dataset pre-processing and coverage”. Open index.html in either folder to view the detailed report data. (ZIP) [file pcbi.1008608.s002.zip › miRNA/Oslo2_QC_Report/out ma.pdf]

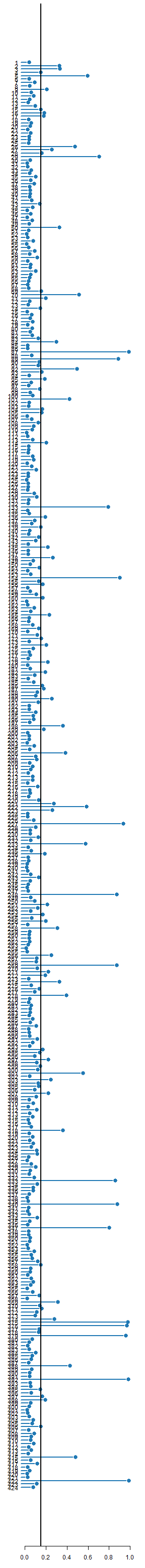

Supplement: S2 Data — Generated by the arrayQualityMetrics package, as described in “Dataset pre-processing and coverage”. Open index.html in either folder to view the detailed report data. (ZIP) [file pcbi.1008608.s002.zip › miRNA/Oslo2_QC_Report/out ma.png]

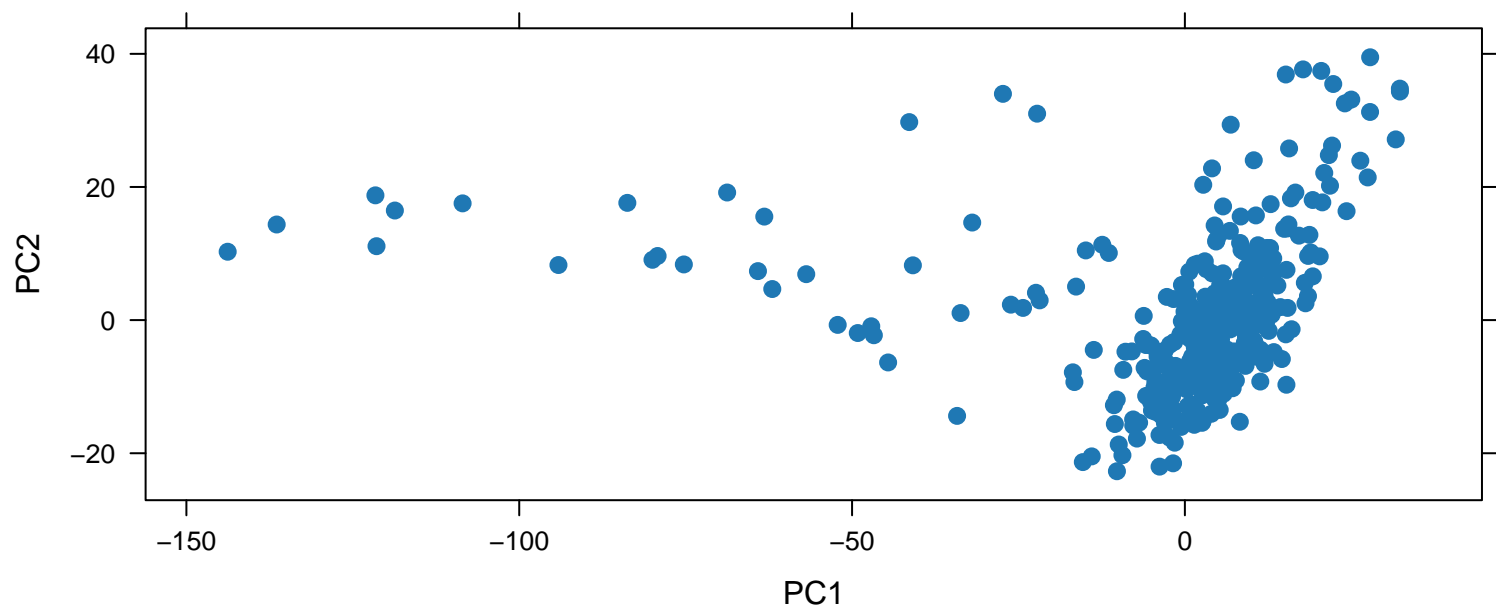

Supplement: S2 Data — Generated by the arrayQualityMetrics package, as described in “Dataset pre-processing and coverage”. Open index.html in either folder to view the detailed report data. (ZIP) [file pcbi.1008608.s002.zip › miRNA/Oslo2_QC_Report/pca.pdf]

Array

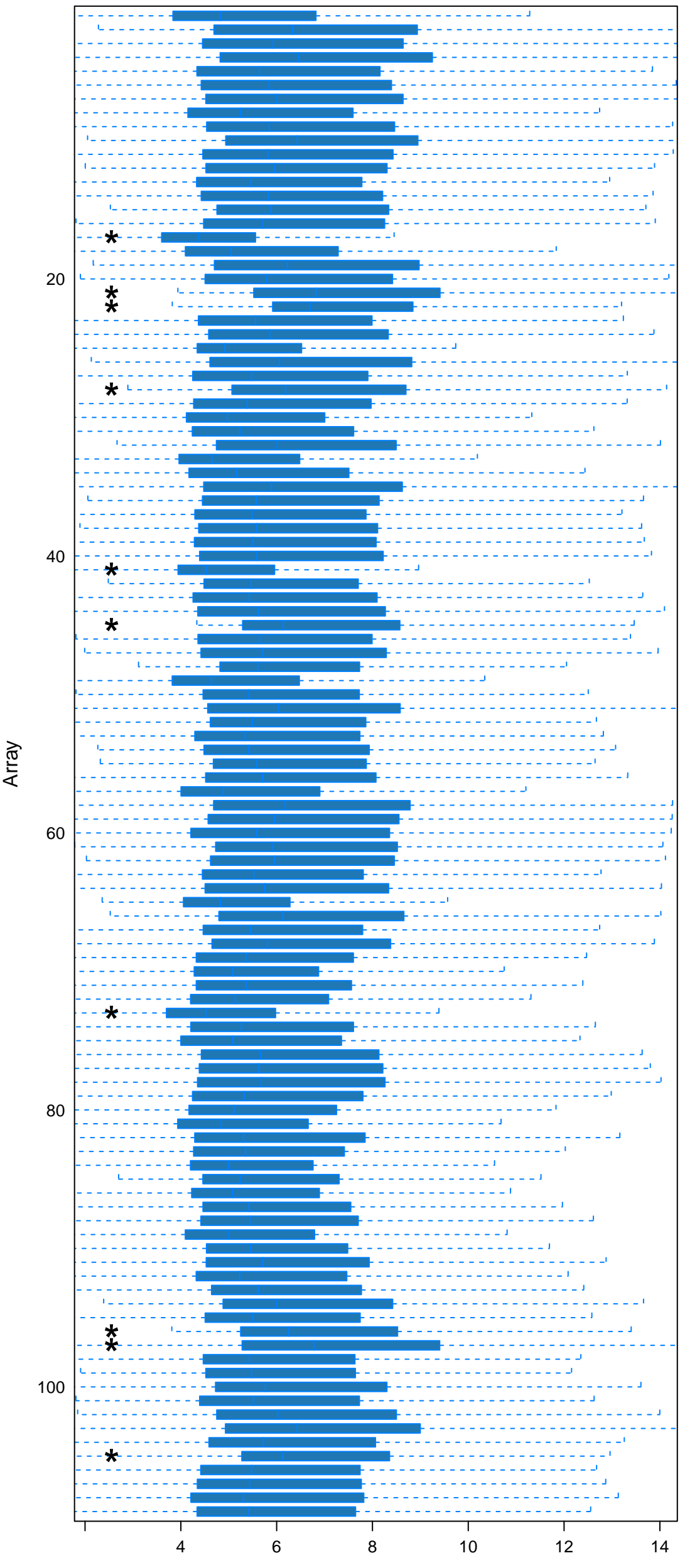

Supplement: S2 Data — Generated by the arrayQualityMetrics package, as described in “Dataset pre-processing and coverage”. Open index.html in either folder to view the detailed report data. (ZIP) [file pcbi.1008608.s002.zip › miRNA/Stavanger_QC_Report/box.pdf]

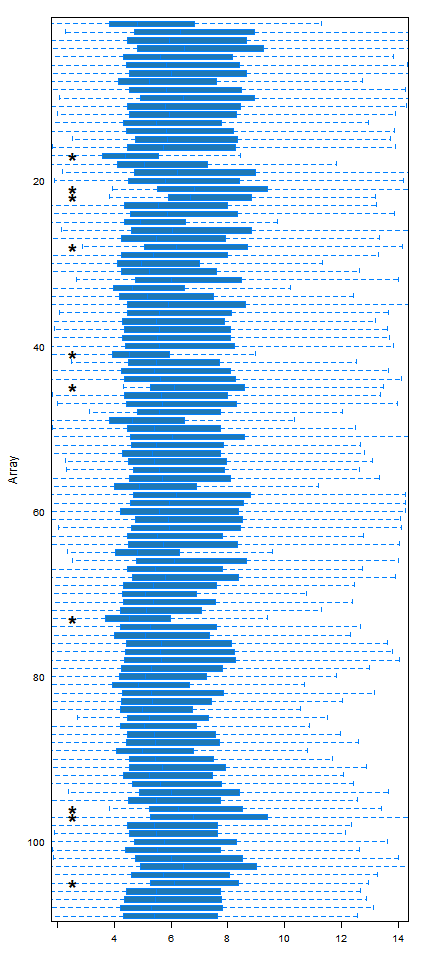

Supplement: S2 Data — Generated by the arrayQualityMetrics package, as described in “Dataset pre-processing and coverage”. Open index.html in either folder to view the detailed report data. (ZIP) [file pcbi.1008608.s002.zip › miRNA/Stavanger_QC_Report/box.png]

Density

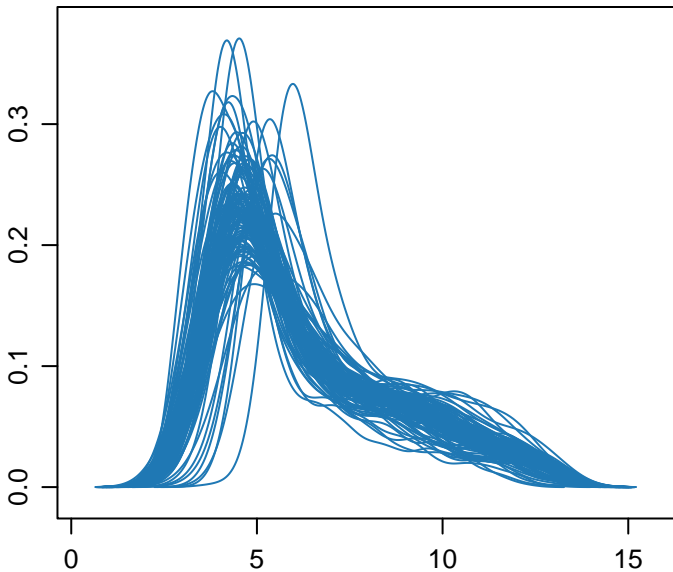

Supplement: S2 Data — Generated by the arrayQualityMetrics package, as described in “Dataset pre-processing and coverage”. Open index.html in either folder to view the detailed report data. (ZIP) [file pcbi.1008608.s002.zip › miRNA/Stavanger_QC_Report/dens.pdf]

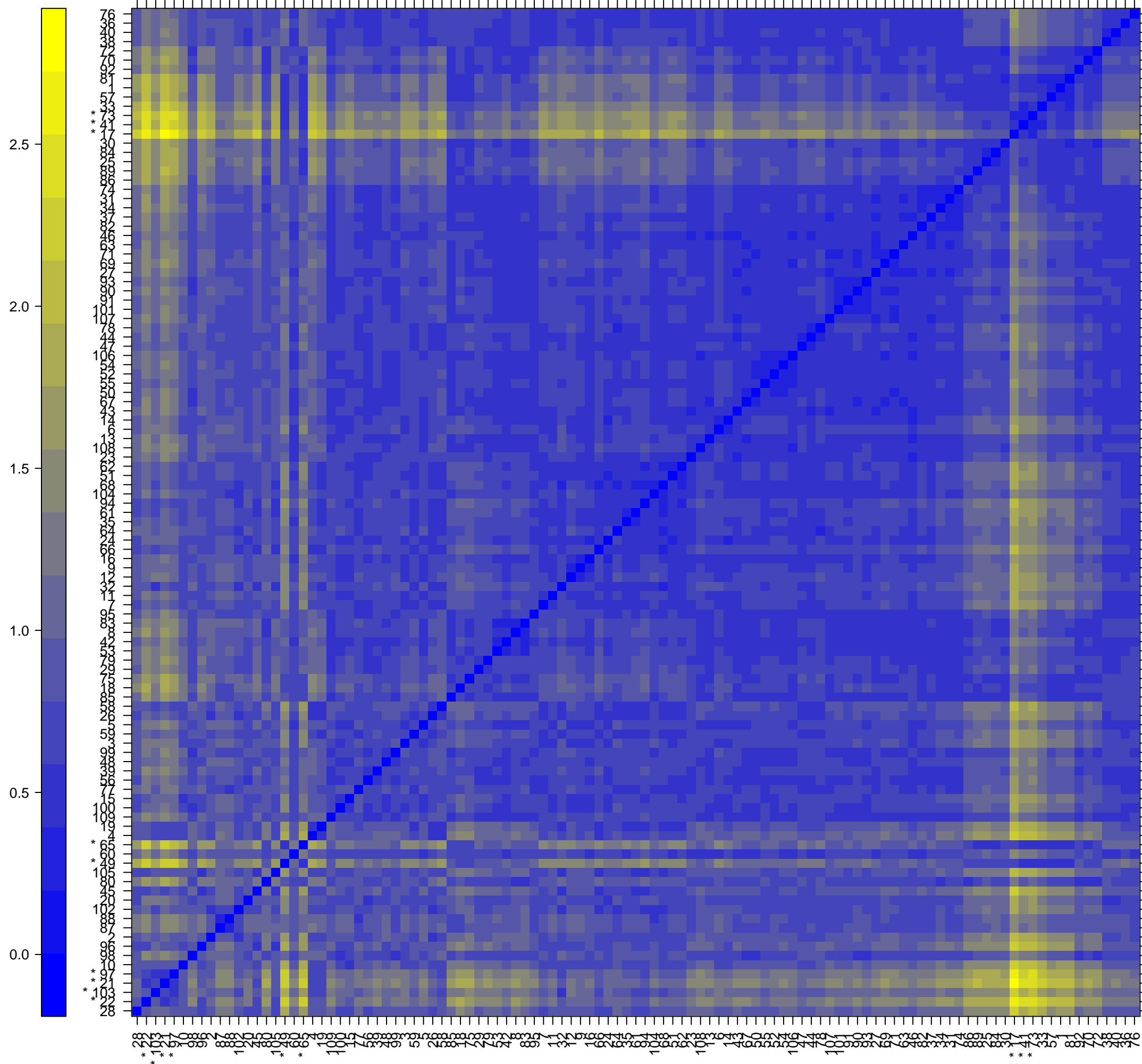

Supplement: S2 Data — Generated by the arrayQualityMetrics package, as described in “Dataset pre-processing and coverage”. Open index.html in either folder to view the detailed report data. (ZIP) [file pcbi.1008608.s002.zip › miRNA/Stavanger_QC_Report/hm.pdf]

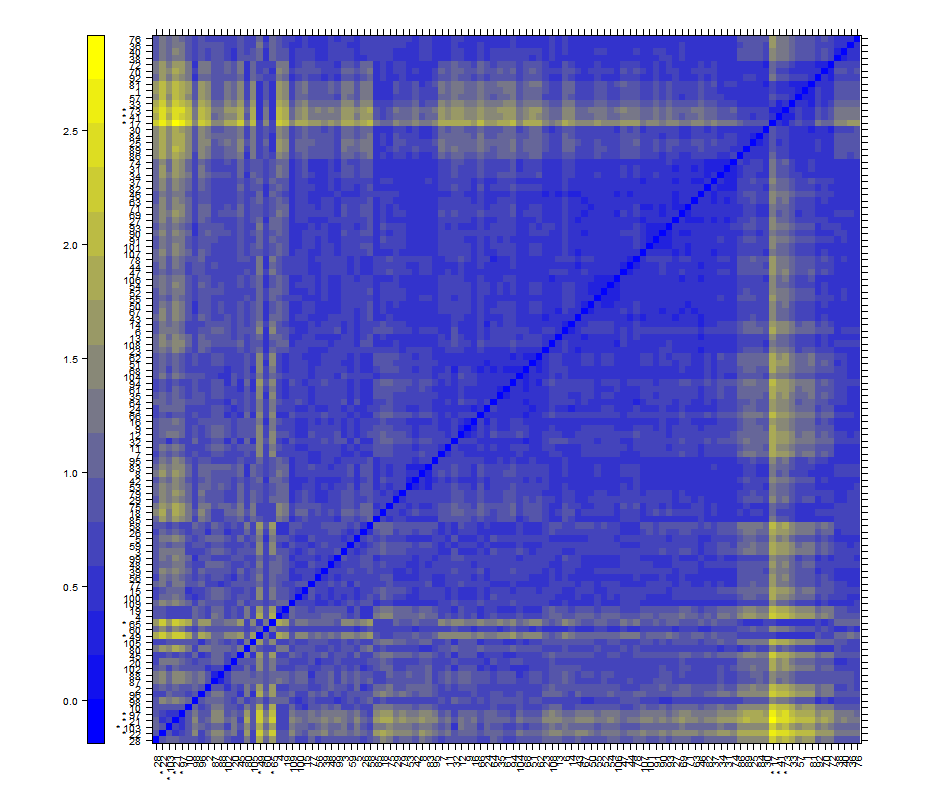

Supplement: S2 Data — Generated by the arrayQualityMetrics package, as described in “Dataset pre-processing and coverage”. Open index.html in either folder to view the detailed report data. (ZIP) [file pcbi.1008608.s002.zip › miRNA/Stavanger_QC_Report/hm.png]

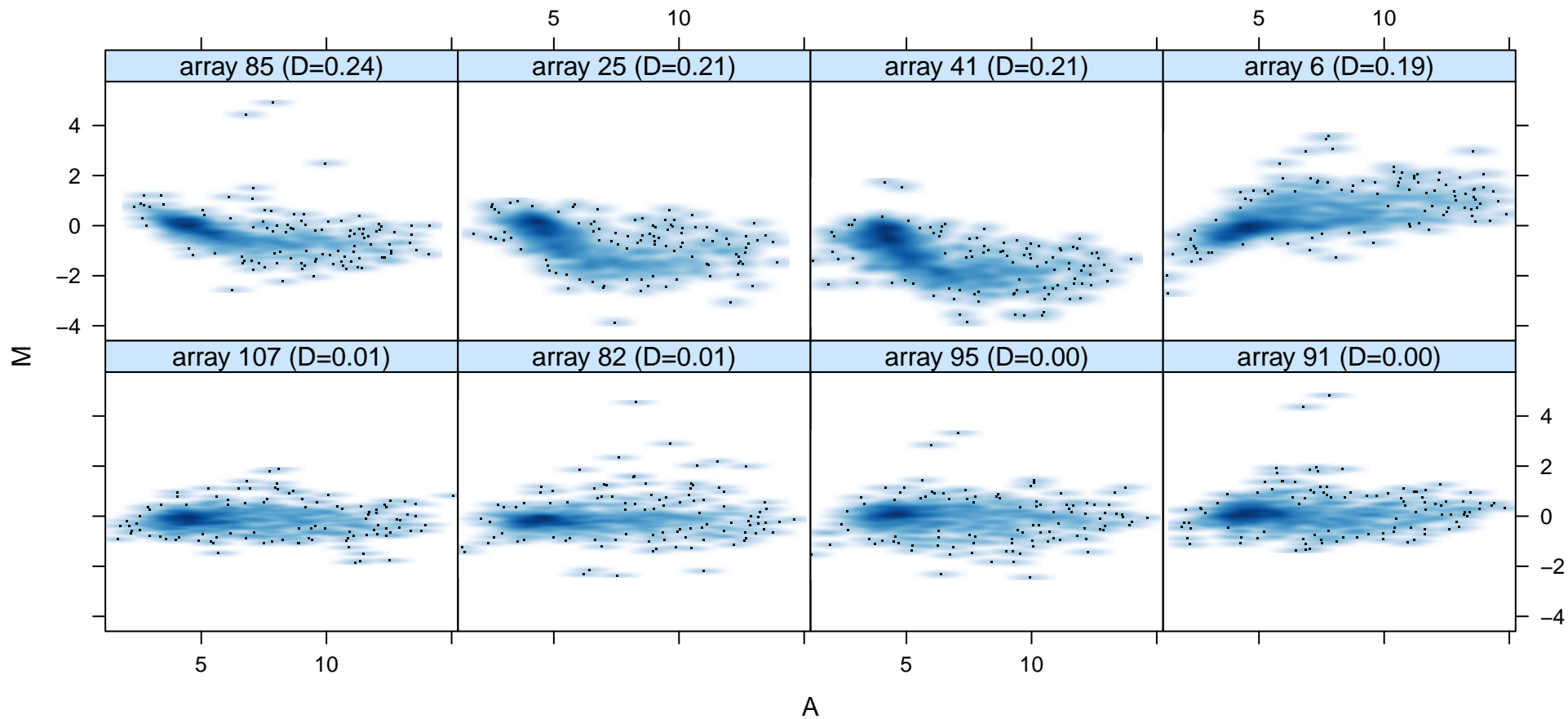

Supplement: S2 Data — Generated by the arrayQualityMetrics package, as described in “Dataset pre-processing and coverage”. Open index.html in either folder to view the detailed report data. (ZIP) [file pcbi.1008608.s002.zip › miRNA/Stavanger_QC_Report/ma.pdf]

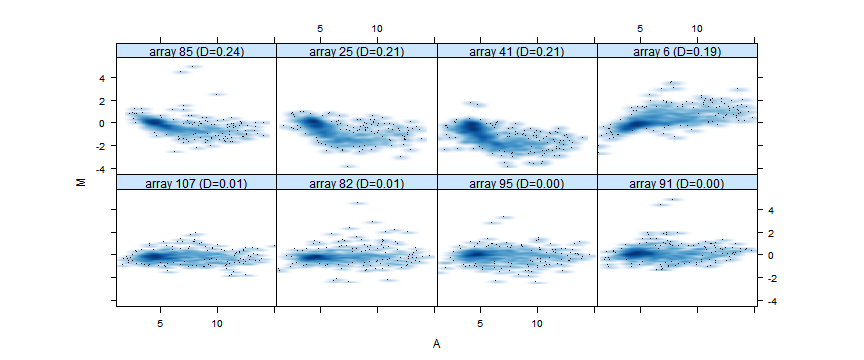

Supplement: S2 Data — Generated by the arrayQualityMetrics package, as described in “Dataset pre-processing and coverage”. Open index.html in either folder to view the detailed report data. (ZIP) [file pcbi.1008608.s002.zip › miRNA/Stavanger_QC_Report/ma.png]

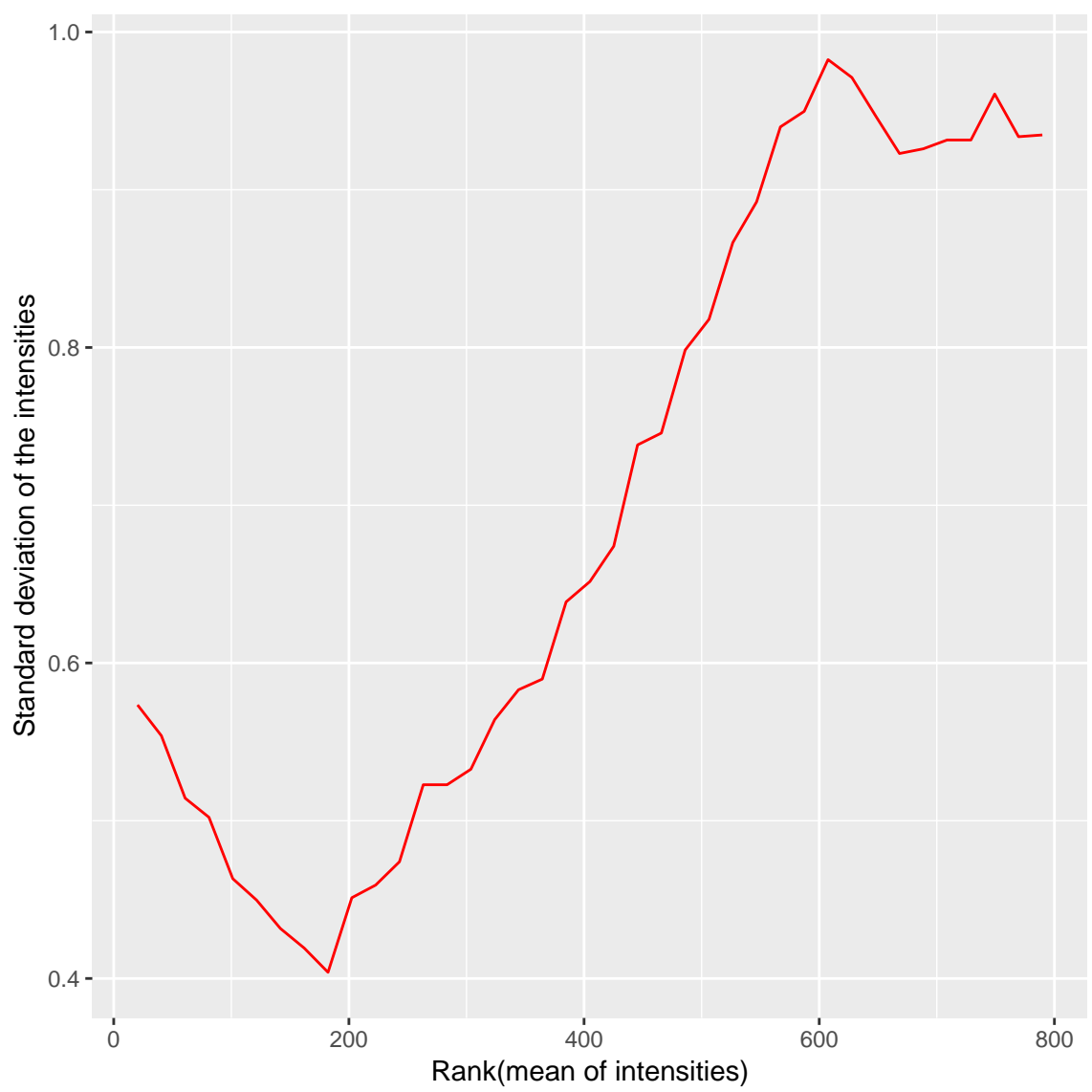

Supplement: S2 Data — Generated by the arrayQualityMetrics package, as described in “Dataset pre-processing and coverage”. Open index.html in either folder to view the detailed report data. (ZIP) [file pcbi.1008608.s002.zip › miRNA/Stavanger_QC_Report/msd.pdf]

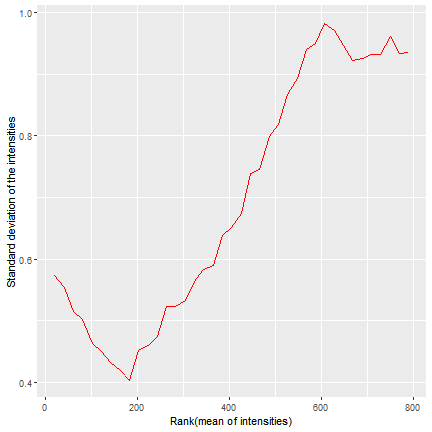

Supplement: S2 Data — Generated by the arrayQualityMetrics package, as described in “Dataset pre-processing and coverage”. Open index.html in either folder to view the detailed report data. (ZIP) [file pcbi.1008608.s002.zip › miRNA/Stavanger_QC_Report/msd.png]

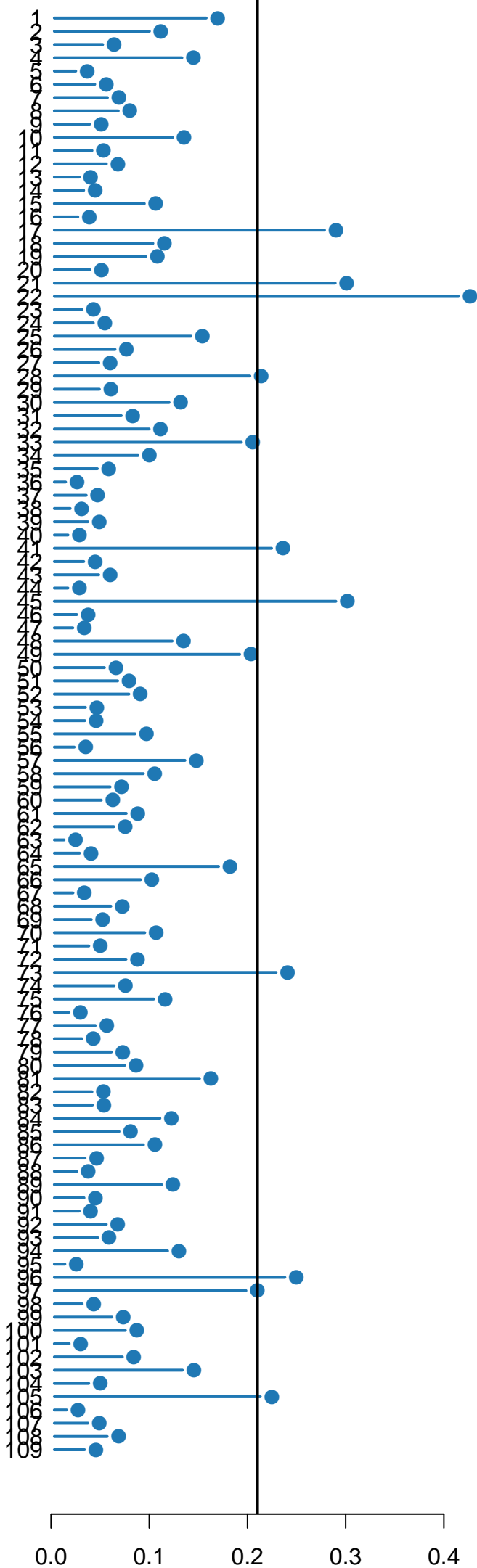

Supplement: S2 Data — Generated by the arrayQualityMetrics package, as described in “Dataset pre-processing and coverage”. Open index.html in either folder to view the detailed report data. (ZIP) [file pcbi.1008608.s002.zip › miRNA/Stavanger_QC_Report/out box.pdf]

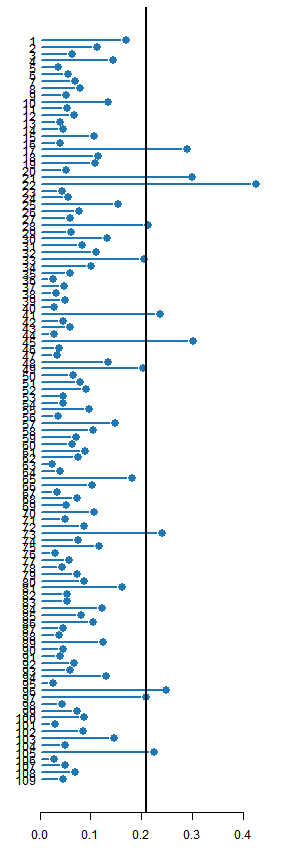

Supplement: S2 Data — Generated by the arrayQualityMetrics package, as described in “Dataset pre-processing and coverage”. Open index.html in either folder to view the detailed report data. (ZIP) [file pcbi.1008608.s002.zip › miRNA/Stavanger_QC_Report/out box.png]

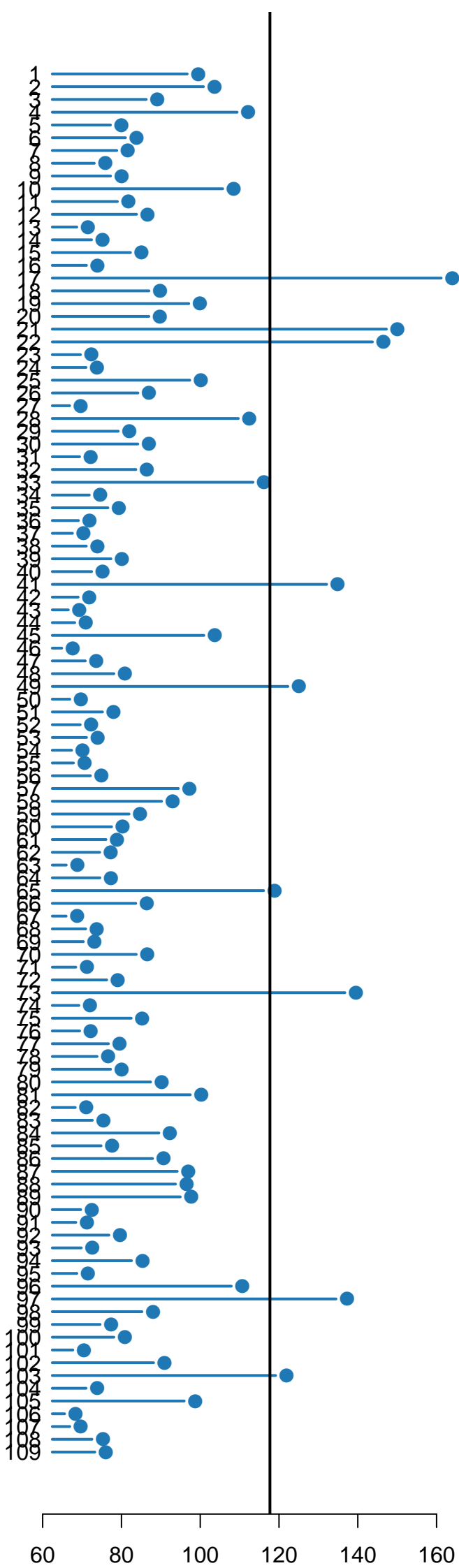

Supplement: S2 Data — Generated by the arrayQualityMetrics package, as described in “Dataset pre-processing and coverage”. Open index.html in either folder to view the detailed report data. (ZIP) [file pcbi.1008608.s002.zip › miRNA/Stavanger_QC_Report/out hm.pdf]
