# Supplementary figures and images for: miRNA normalization enables joint analysis of several datasets to increase sensitivity and to reveal novel miRNAs differentially expressed in breast cancer (part 2 of 2)
Source: PLoS Comput Biol. 2021 Feb 10;17(2):e1008608. doi: 10.1371/journal.pcbi.1008608 (PMC7901788; doi:10.1371/journal.pcbi.1008608)

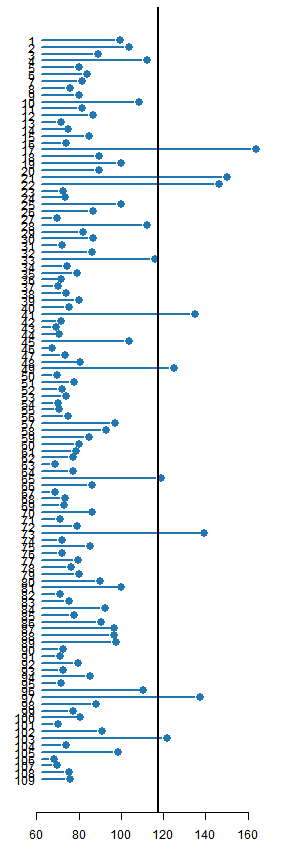

Supplement: S2 Data — Generated by the arrayQualityMetrics package, as described in “Dataset pre-processing and coverage”. Open index.html in either folder to view the detailed report data. (ZIP) [file pcbi.1008608.s002.zip › miRNA/Stavanger_QC_Report/out hm.png]

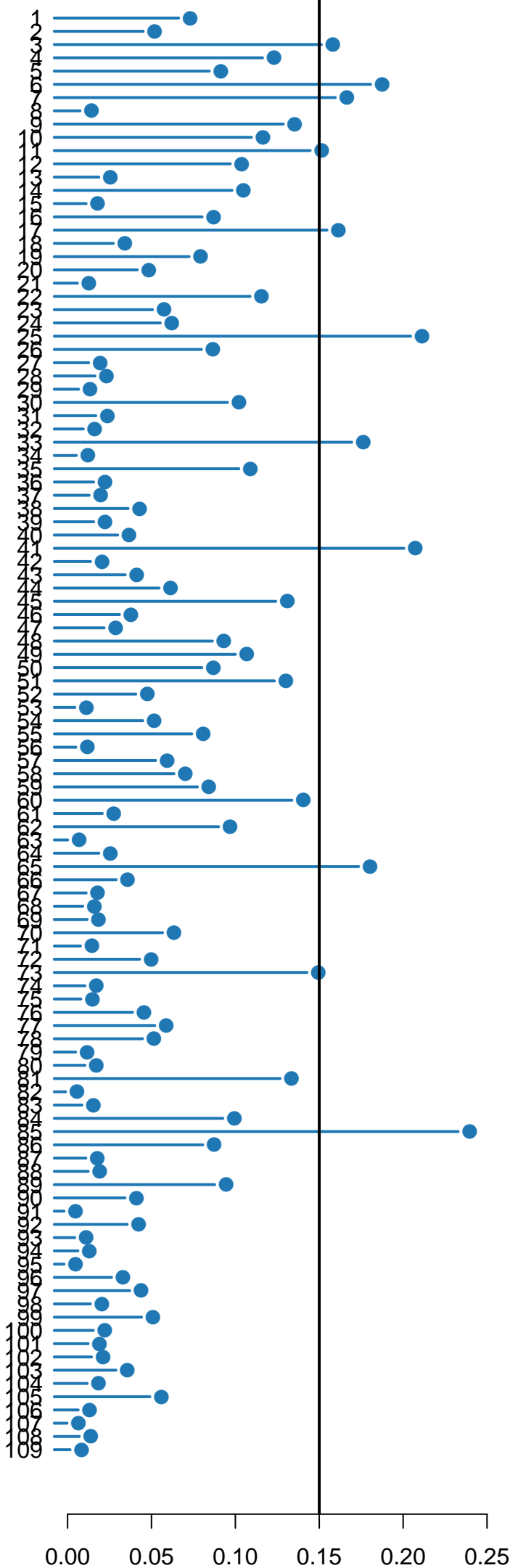

Supplement: S2 Data — Generated by the arrayQualityMetrics package, as described in “Dataset pre-processing and coverage”. Open index.html in either folder to view the detailed report data. (ZIP) [file pcbi.1008608.s002.zip › miRNA/Stavanger_QC_Report/out ma.pdf]

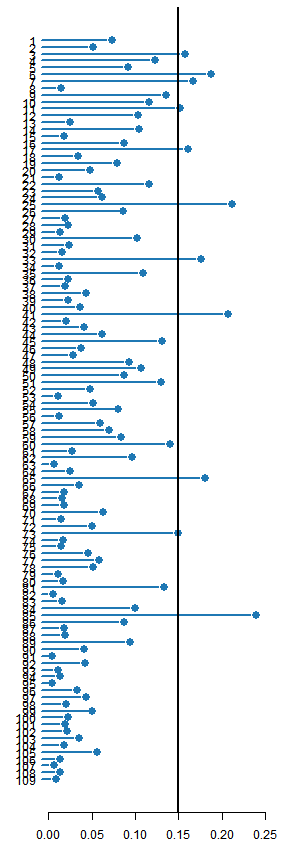

Supplement: S2 Data — Generated by the arrayQualityMetrics package, as described in “Dataset pre-processing and coverage”. Open index.html in either folder to view the detailed report data. (ZIP) [file pcbi.1008608.s002.zip › miRNA/Stavanger_QC_Report/out ma.png]

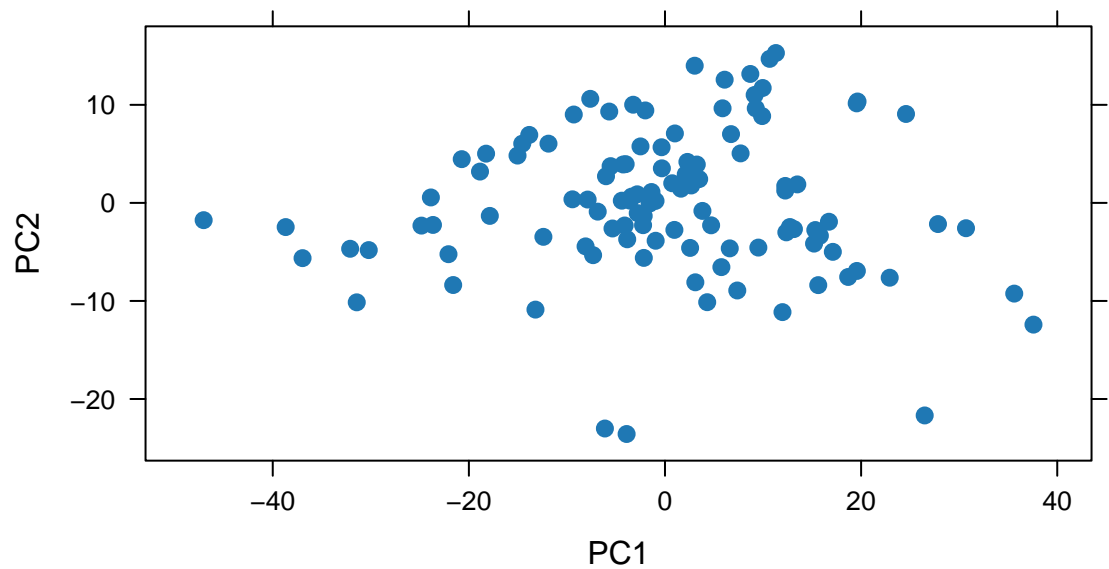

Supplement: S2 Data — Generated by the arrayQualityMetrics package, as described in “Dataset pre-processing and coverage”. Open index.html in either folder to view the detailed report data. (ZIP) [file pcbi.1008608.s002.zip › miRNA/Stavanger_QC_Report/pca.pdf]

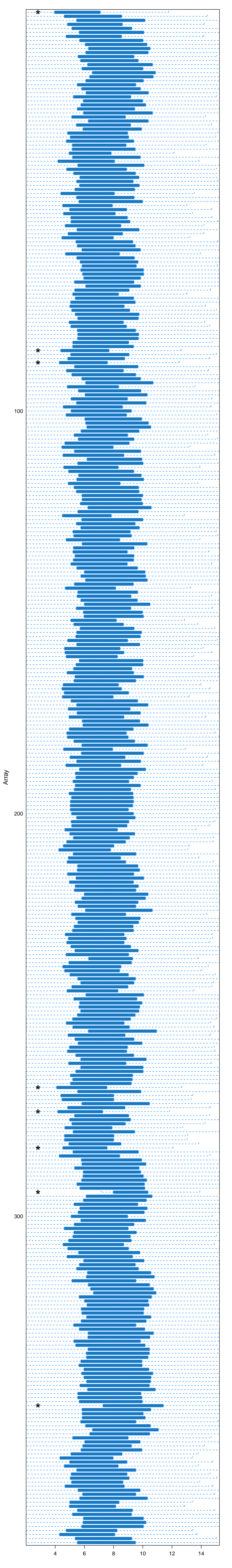

Supplement: S3 Data — Generated by the arrayQualityMetrics package, as described in “Dataset pre-processing and coverage”. Open index.html in either folder to view the detailed report data. (ZIP) [file pcbi.1008608.s003.zip › mRNA/Oslo2_QC_Report/box.pdf]

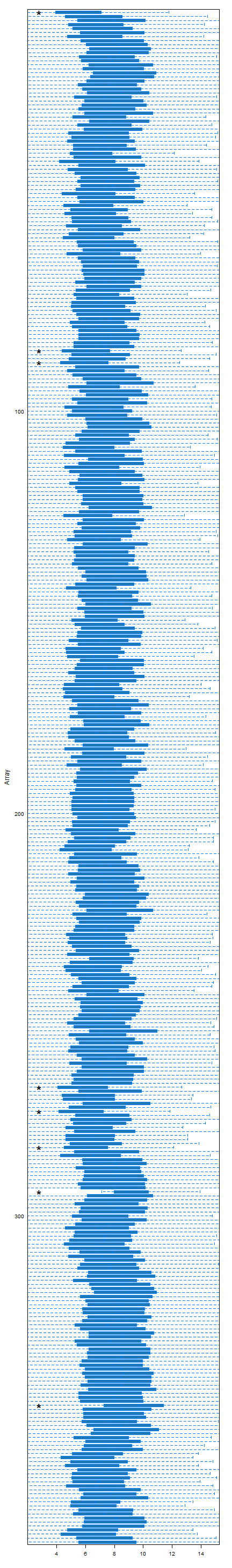

Supplement: S3 Data — Generated by the arrayQualityMetrics package, as described in “Dataset pre-processing and coverage”. Open index.html in either folder to view the detailed report data. (ZIP) [file pcbi.1008608.s003.zip › mRNA/Oslo2_QC_Report/box.png]

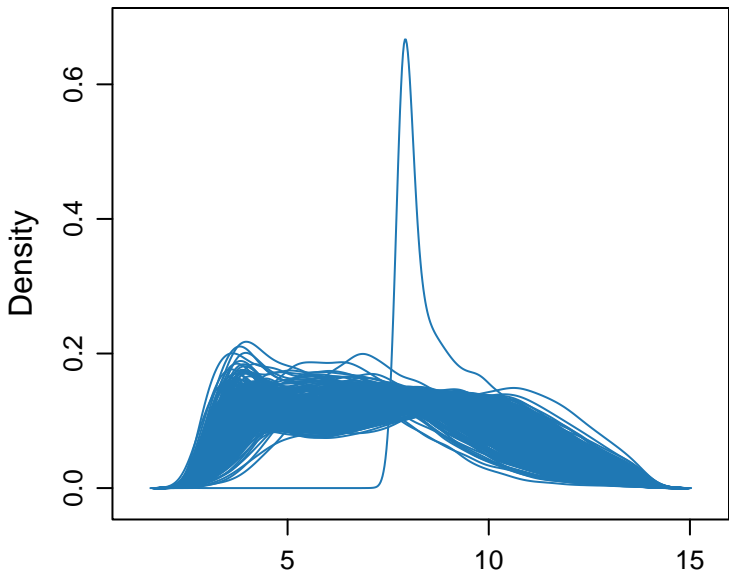

Supplement: S3 Data — Generated by the arrayQualityMetrics package, as described in “Dataset pre-processing and coverage”. Open index.html in either folder to view the detailed report data. (ZIP) [file pcbi.1008608.s003.zip › mRNA/Oslo2_QC_Report/dens.pdf]

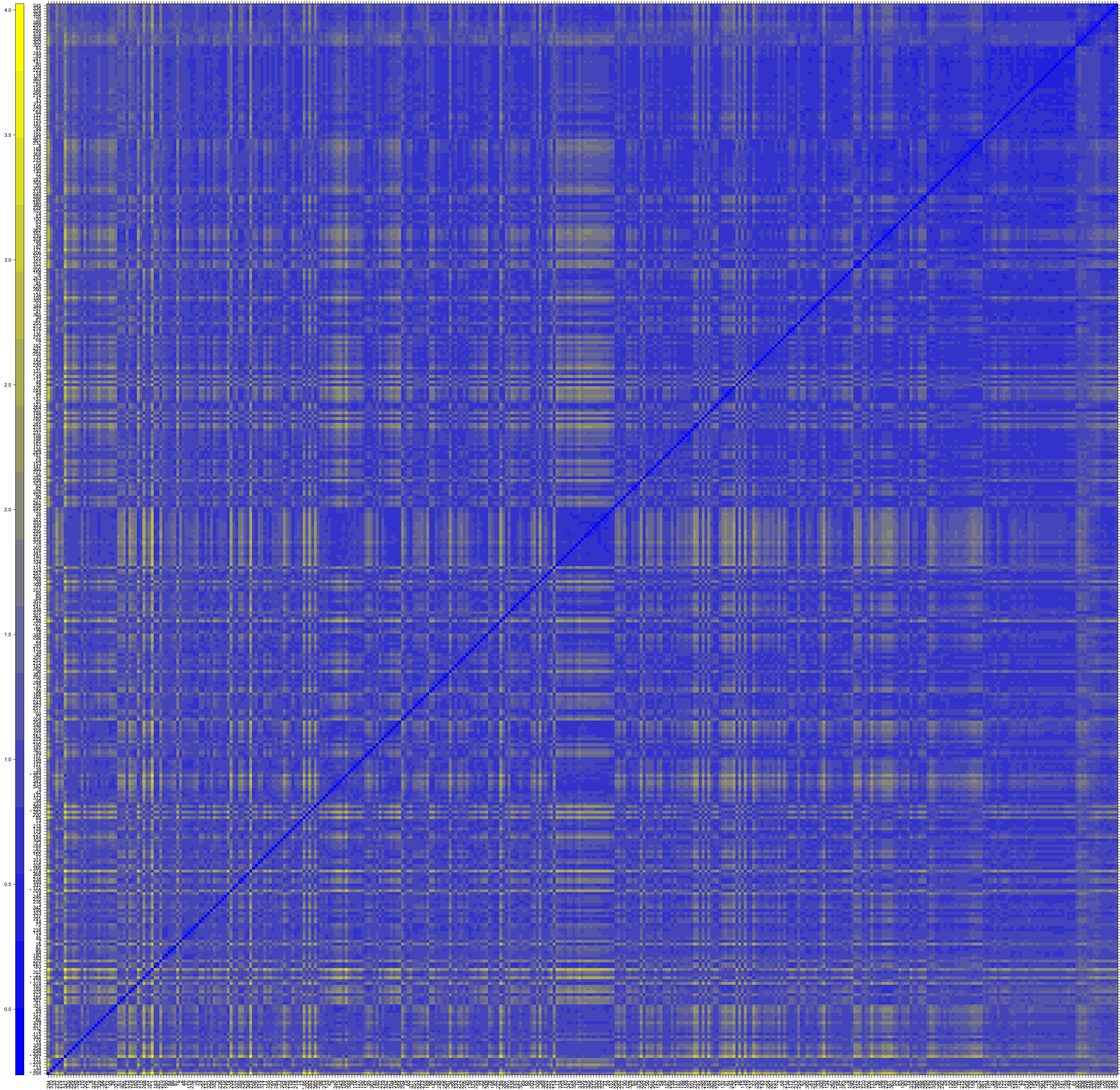

Supplement: S3 Data — Generated by the arrayQualityMetrics package, as described in “Dataset pre-processing and coverage”. Open index.html in either folder to view the detailed report data. (ZIP) [file pcbi.1008608.s003.zip › mRNA/Oslo2_QC_Report/hm.pdf]

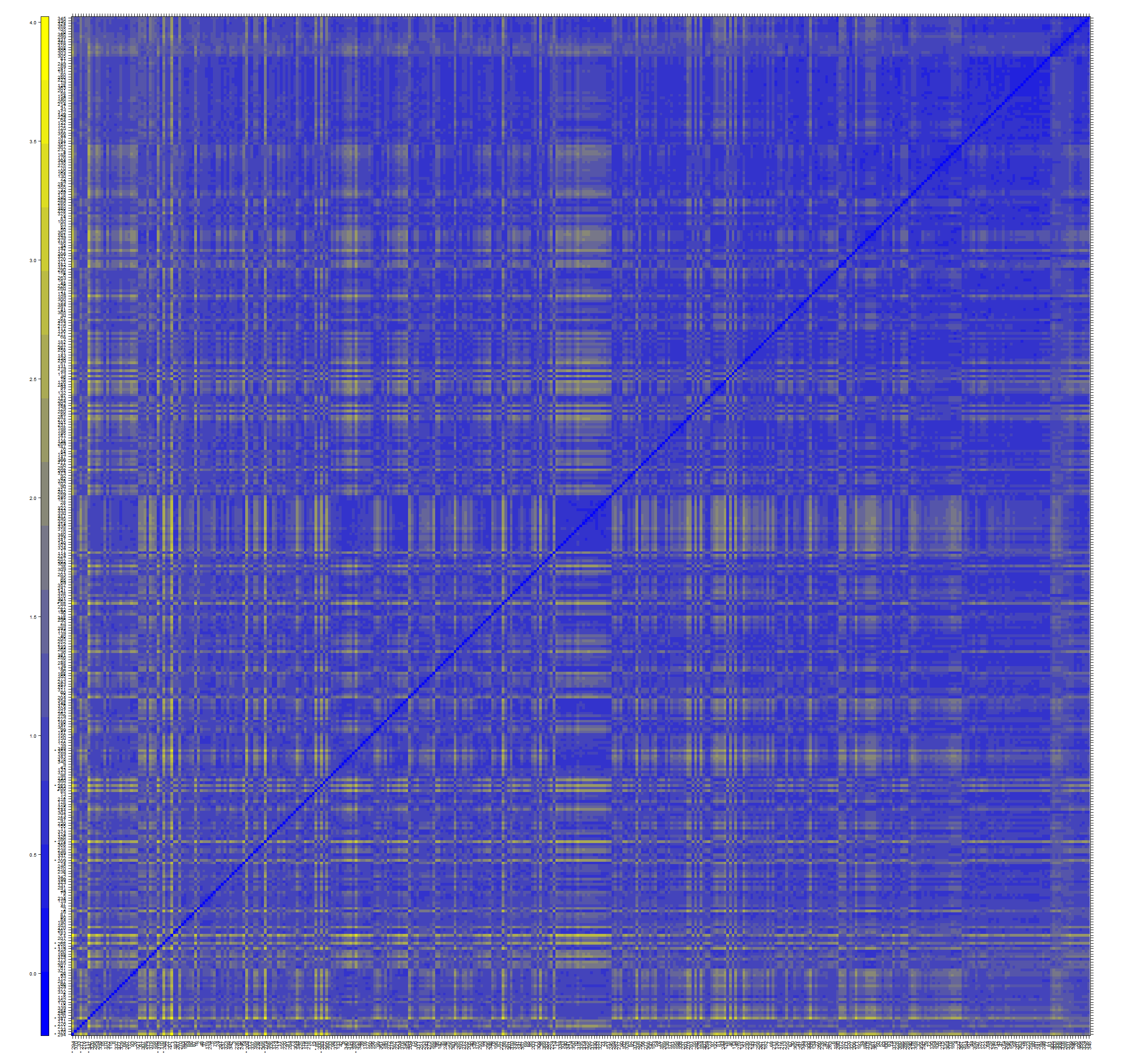

Supplement: S3 Data — Generated by the arrayQualityMetrics package, as described in “Dataset pre-processing and coverage”. Open index.html in either folder to view the detailed report data. (ZIP) [file pcbi.1008608.s003.zip › mRNA/Oslo2_QC_Report/hm.png]

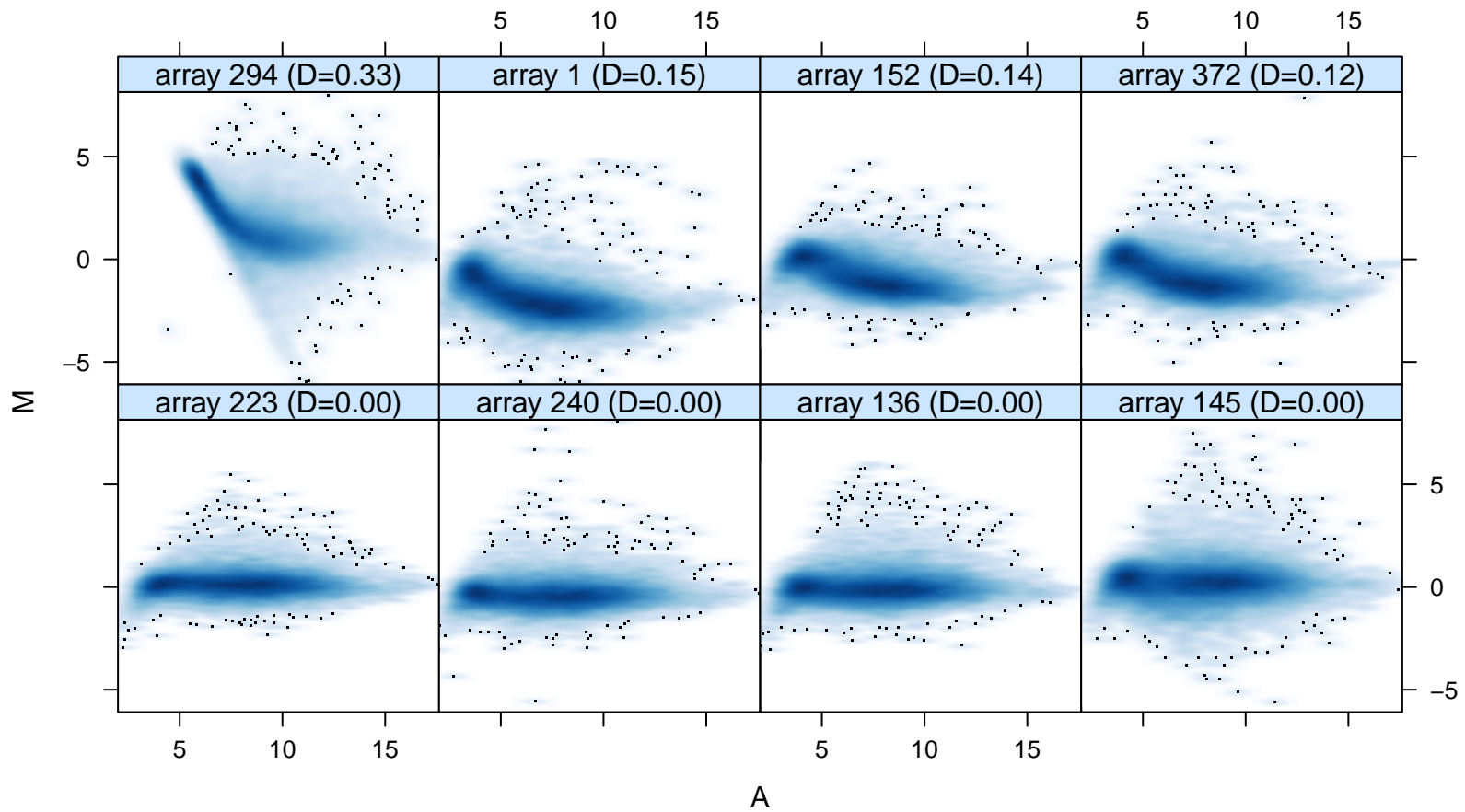

Supplement: S3 Data — Generated by the arrayQualityMetrics package, as described in “Dataset pre-processing and coverage”. Open index.html in either folder to view the detailed report data. (ZIP) [file pcbi.1008608.s003.zip › mRNA/Oslo2_QC_Report/ma.pdf]

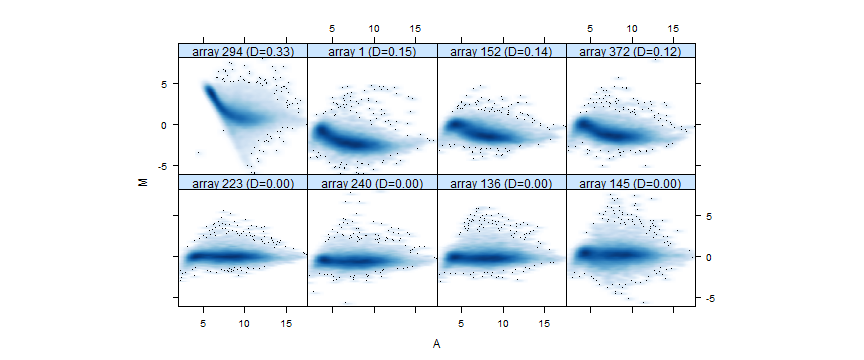

Supplement: S3 Data — Generated by the arrayQualityMetrics package, as described in “Dataset pre-processing and coverage”. Open index.html in either folder to view the detailed report data. (ZIP) [file pcbi.1008608.s003.zip › mRNA/Oslo2_QC_Report/ma.png]

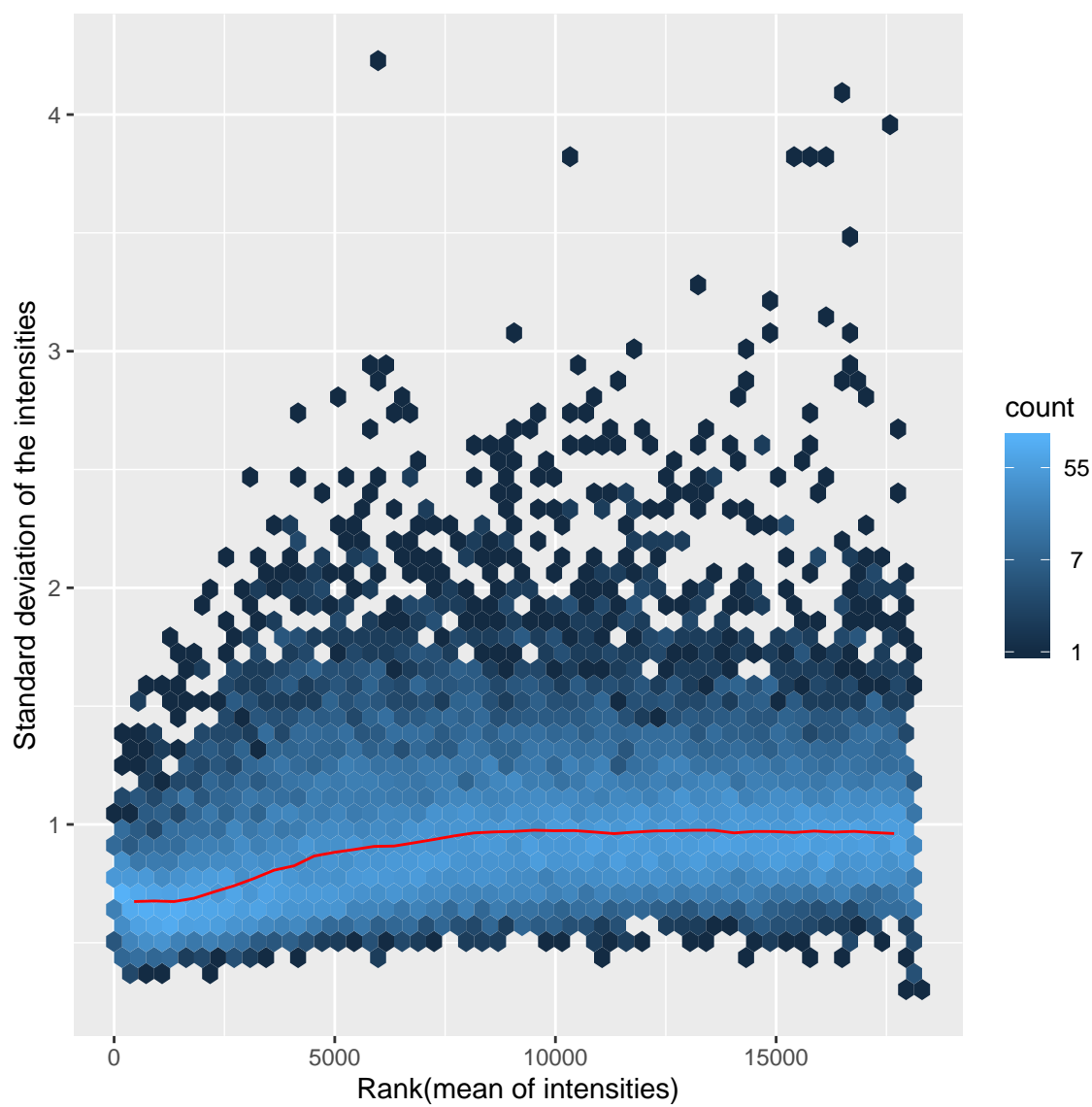

Supplement: S3 Data — Generated by the arrayQualityMetrics package, as described in “Dataset pre-processing and coverage”. Open index.html in either folder to view the detailed report data. (ZIP) [file pcbi.1008608.s003.zip › mRNA/Oslo2_QC_Report/msd.pdf]

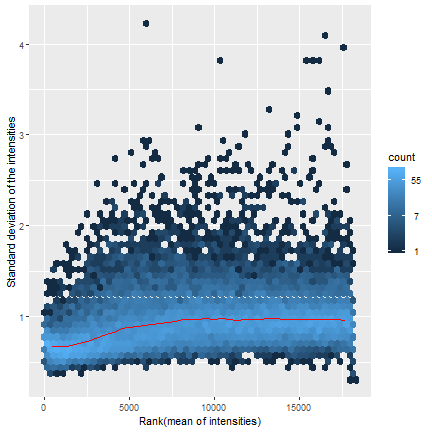

Supplement: S3 Data — Generated by the arrayQualityMetrics package, as described in “Dataset pre-processing and coverage”. Open index.html in either folder to view the detailed report data. (ZIP) [file pcbi.1008608.s003.zip › mRNA/Oslo2_QC_Report/msd.png]

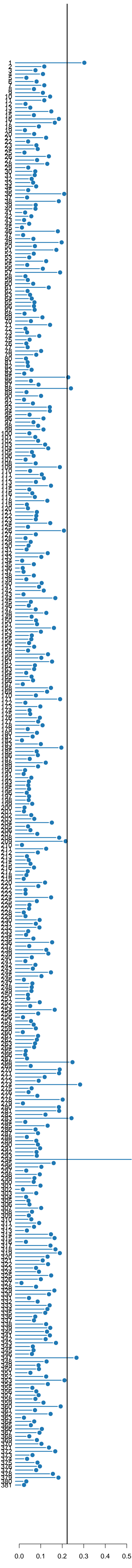

Supplement: S3 Data — Generated by the arrayQualityMetrics package, as described in “Dataset pre-processing and coverage”. Open index.html in either folder to view the detailed report data. (ZIP) [file pcbi.1008608.s003.zip › mRNA/Oslo2_QC_Report/out box.pdf]

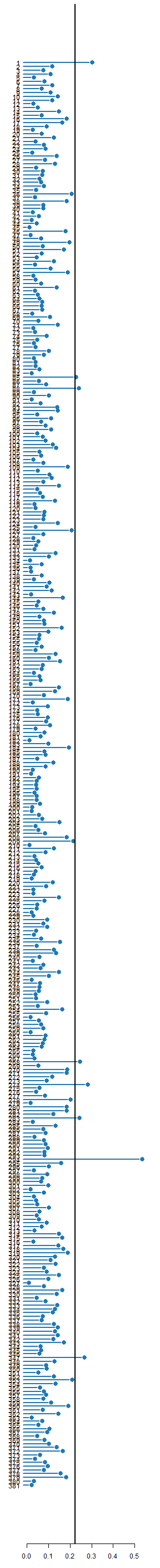

Supplement: S3 Data — Generated by the arrayQualityMetrics package, as described in “Dataset pre-processing and coverage”. Open index.html in either folder to view the detailed report data. (ZIP) [file pcbi.1008608.s003.zip › mRNA/Oslo2_QC_Report/out box.png]

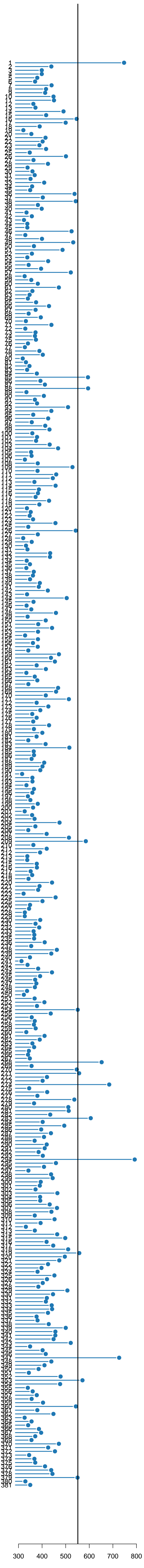

Supplement: S3 Data — Generated by the arrayQualityMetrics package, as described in “Dataset pre-processing and coverage”. Open index.html in either folder to view the detailed report data. (ZIP) [file pcbi.1008608.s003.zip › mRNA/Oslo2_QC_Report/out hm.pdf]

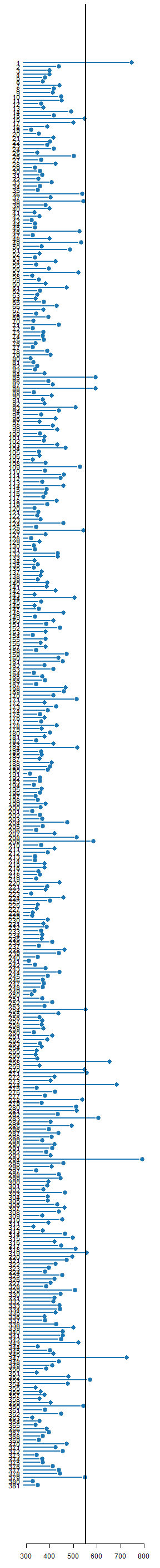

Supplement: S3 Data — Generated by the arrayQualityMetrics package, as described in “Dataset pre-processing and coverage”. Open index.html in either folder to view the detailed report data. (ZIP) [file pcbi.1008608.s003.zip › mRNA/Oslo2_QC_Report/out hm.png]

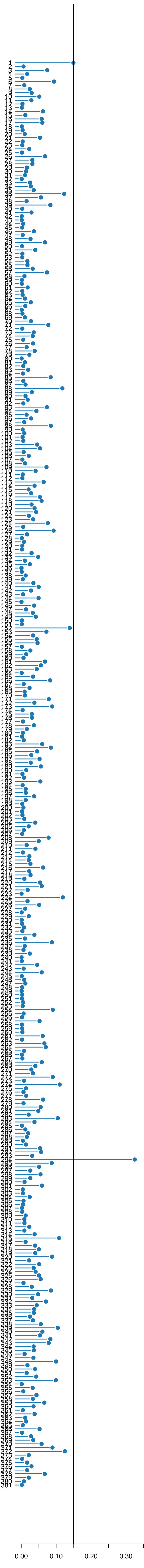

Supplement: S3 Data — Generated by the arrayQualityMetrics package, as described in “Dataset pre-processing and coverage”. Open index.html in either folder to view the detailed report data. (ZIP) [file pcbi.1008608.s003.zip › mRNA/Oslo2_QC_Report/out ma.pdf]

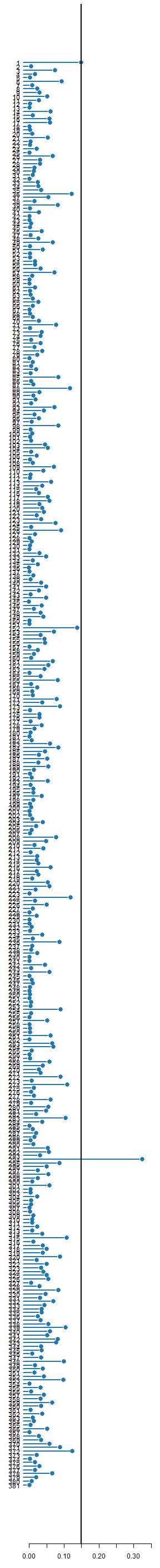

Supplement: S3 Data — Generated by the arrayQualityMetrics package, as described in “Dataset pre-processing and coverage”. Open index.html in either folder to view the detailed report data. (ZIP) [file pcbi.1008608.s003.zip › mRNA/Oslo2_QC_Report/out ma.png]

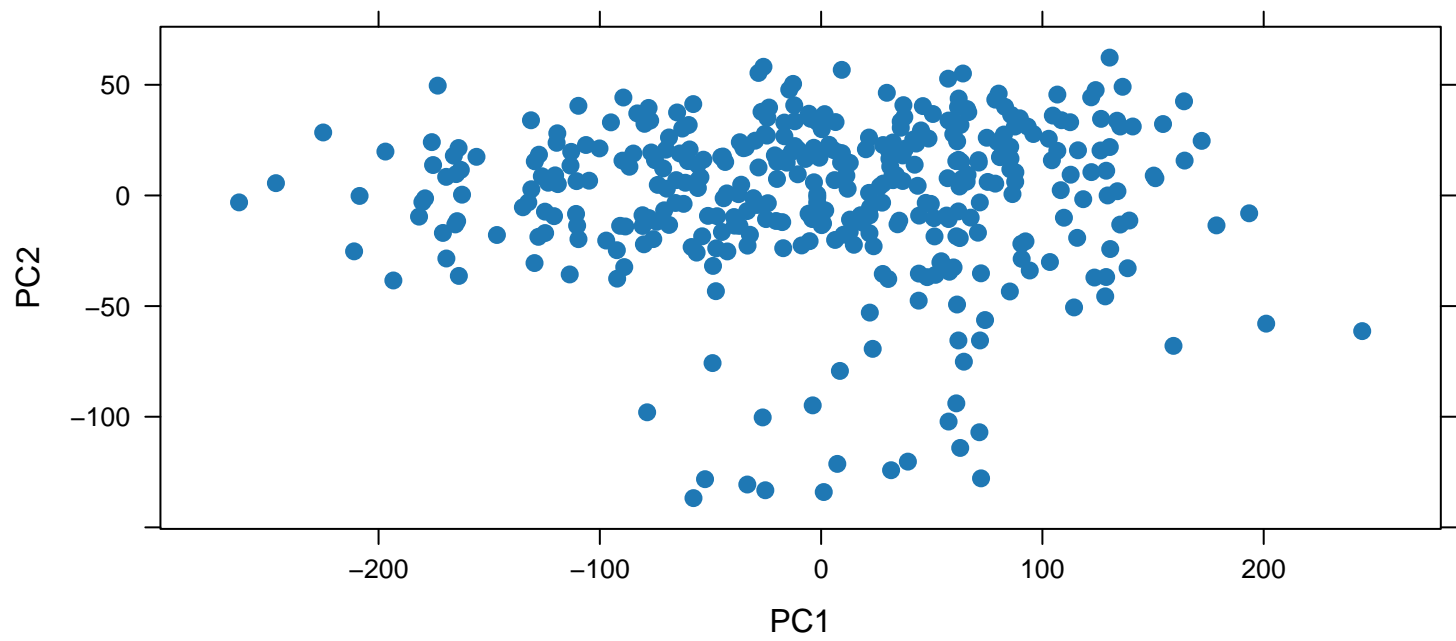

Supplement: S3 Data — Generated by the arrayQualityMetrics package, as described in “Dataset pre-processing and coverage”. Open index.html in either folder to view the detailed report data. (ZIP) [file pcbi.1008608.s003.zip › mRNA/Oslo2_QC_Report/pca.pdf]

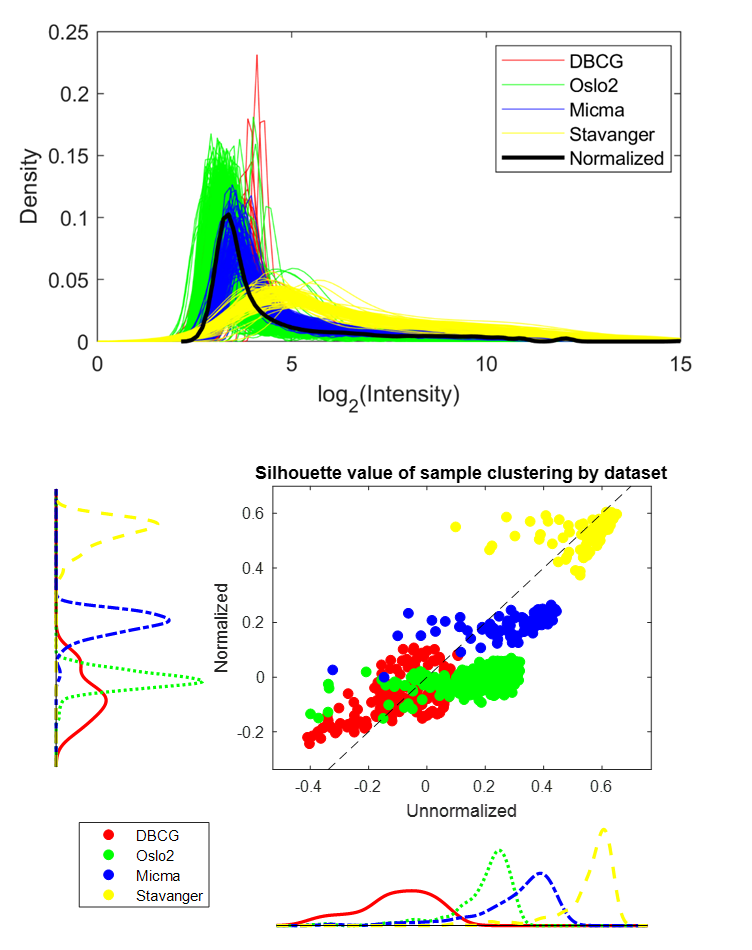

Supplement: S1 Fig — Top) Kernel density estimates of each sample colored by their corresponding dataset. The resulting normalized distribution is overlaid in black. Bottom) Impact of normalization on per-sample silhouette coefficient measured for clustering by dataset. 602/745 samples have lower silhouette coefficients after normalization in comparison to before normalization, demonstrating an overall alleviation of batch effect per dataset. Marginal distributions are shown to highlight differences between datasets. (TIF) [file pcbi.1008608.s004.tif]

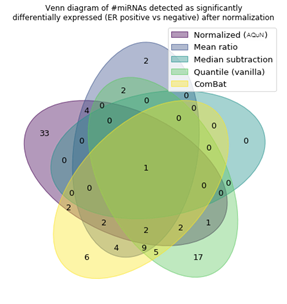

Supplement: S2 Fig — We observe a larger set of unique miRNAs detected by our normalization approach compared to other approaches. (TIF) [file pcbi.1008608.s005.tif]

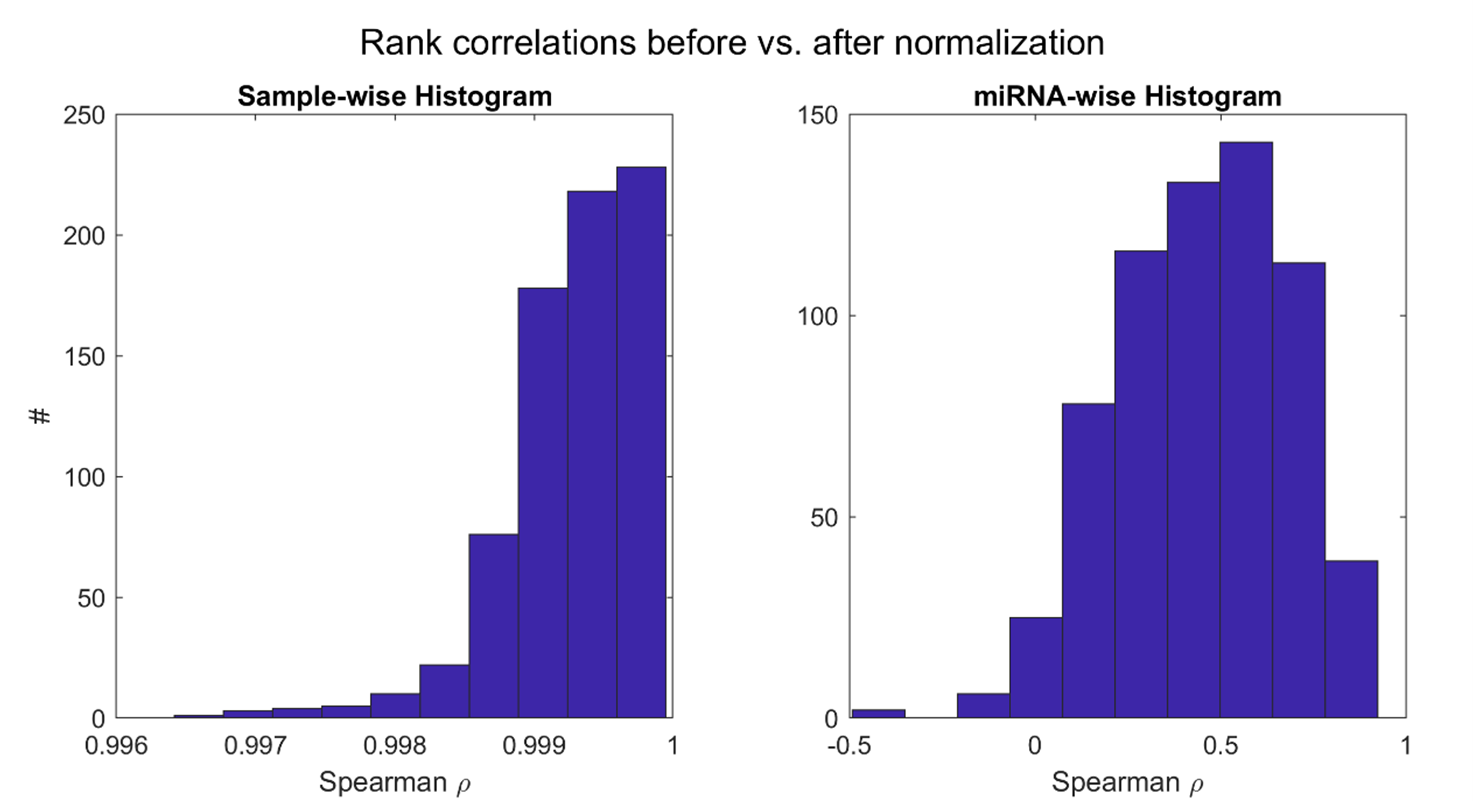

Supplement: S3 Fig — (TIF) [file pcbi.1008608.s006.tif]

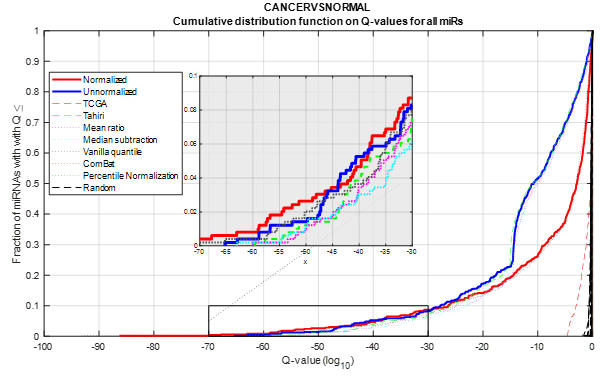

Supplement: S4 Fig — The results shown here follow the same analysis described in Fig 3 in the manuscript. We note the additional evaluated method “Percentile Normalization” was added as it is only relevant in a case-vs-control setup, as evaluated here. Note that the “Percentile Normalization” curve is overlaid by the random permutation curves (dashed black curves). (TIF) [file pcbi.1008608.s007.tif]
