# Supplementary material for: miRNA normalization enables joint analysis of several datasets to increase sensitivity and to reveal novel miRNAs differentially expressed in breast cancer
Source: PLoS Comput Biol. 2021 Feb 10;17(2):e1008608. doi: 10.1371/journal.pcbi.1008608 (PMC7901788; doi:10.1371/journal.pcbi.1008608)
Supplement: S1 Text — (DOCX) [file pcbi.1008608.s015.docx]

Joint one-colored and two-colored analysis.

Stavanger dataset contains a second color with pooled samples deliberately left out of our analysis. Our downstream statistics are rank-based, assuming that, within a margin of error, identical samples measured with different technologies produce similar ranked miRNA vectors. Normalized Stavanger data using a pool reference second channel would cause substantial re-rankings. E.g. housekeeping, or constitutive miRNAs that are highly expressed would effectively “cancel out”, and differently expressed miRNAs compared to the background would emerge instead. Therefore, to avoid an apples-to-oranges comparison, we decided to neglect the background expression data available in Stavanger from our analysis.
