# Supplementary material for: miRNA normalization enables joint analysis of several datasets to increase sensitivity and to reveal novel miRNAs differentially expressed in breast cancer
Source: PLoS Comput Biol. 2021 Feb 10;17(2):e1008608. doi: 10.1371/journal.pcbi.1008608 (PMC7901788; doi:10.1371/journal.pcbi.1008608)
Supplement: S2 Text — (DOCX) [file pcbi.1008608.s016.docx]

Venn diagram showing the differential expression results (comparing estrogen receptor (ER) positive and negative tumors) of all normalization methods considered.
