# Supplementary material for: miRNA normalization enables joint analysis of several datasets to increase sensitivity and to reveal novel miRNAs differentially expressed in breast cancer
Source: PLoS Comput Biol. 2021 Feb 10;17(2):e1008608. doi: 10.1371/journal.pcbi.1008608 (PMC7901788; doi:10.1371/journal.pcbi.1008608)
Supplement: S3 Text — (DOCX) [file pcbi.1008608.s017.docx]

AQuN normalization impact on data rankings

We distinguish between sample-wise (a.k.a. column-wise) and miRNA-wise (a.k.a. row-wise) impact. Sample-wise, we apply a monotonic transformation of raw expression values per sample which should not affect rankings of miRNAs within each sample. As we observe in S3 Fig, Left we see these samples almost fully correlated before and after normalization. The minor differences are owed to two effects – jitter and quantization. Jitter can swap miRNA ranks within a sample, especially for miRNA with low expression compared to our jitter scale. We pre-process the data by min-max normalization and select a jitter scale such that ranks are mostly unaffected by jitter. A stronger impact is due to quantization which replaces values within the same percentile with a cross-sample median, creating ties.

miRNA-wise there are no guarantees of monotonicity, as evident in S3 Fig, Right and as shown in improved results for analyses such as differential expression in Fig 3.
