# Supplementary material for: miRNA normalization enables joint analysis of several datasets to increase sensitivity and to reveal novel miRNAs differentially expressed in breast cancer
Source: PLoS Comput Biol. 2021 Feb 10;17(2):e1008608. doi: 10.1371/journal.pcbi.1008608 (PMC7901788; doi:10.1371/journal.pcbi.1008608)
Supplement: Attachment — Submitted filename: Response_to_reviewers.docx [file pcbi.1008608.s018.docx]

**Reviewer #1:**

In the manuscript (ID PCOMPBIOL-D-20-00931), authors present a non parametric normalization method, called AQuN for Adjusted Quantile Normalization, to combine miRNA expression data from multiple breast cancer datasets into a joint dataset. They detect 33 miRNAs to be differentially expressed in estrogen receptor (ER) positive versus ER negative samples that do not emerge as statistically significant when separately analyzing these datasets. In particular, they perform miRNA mimics assays on breast cancer cell lines to validate the effect of has-miR-193b-5p on cell viability and apoptosis.

Although the article deals with a relevant topic, such as integrative analysis of microRNA expression data, it does contain major issues that require to be addressed:

1) The authors evaluate the impact of the AQuN normalization on batch effect correction based on more significant results obtained in differential expression analysis (lower Q-values) for most miRNAs. This is not conclusive to determine to what extent the impact of normalization is beneficial. It would be more appropriate to apply a test metric for batch correction such as adjusted rand index (ARI) or others to quantify the extent to which AQuN normalization removes batch effects while increasing subtyping accuracy.

**Response**: We agree with the reviewer that there is room to expand the analysis to better determine the impact of normalization on batch effects in the data. However, ARI is a measure that compares cluster assignments with ground truth clusters. To apply ARI to our data would first require clustering it, unnecessarily compounding to the problem of measuring normalization impact on similarity within and between batch groups. We offer an alternative approach to ARI using Silhouette values (Fig 10b. and Supplementary Fig1), which sidesteps the need for clustering and provides a similar view into the impact AQuN has on batch effects.

2) Performances of the proposed AQuN normalization method should be more extensively compared with other existing methods, including the non-parametric approach cited in [22], in terms of their efficacy and efficiency to integrate batches while maintaining tumor subtypes separation.

**Response**: The method described in [22] uses a case/control set up which is less interesting in the context of differential expression (DE) – our main interest. We do compare our results to those obtained from standard quantile normalization approaches, as the reviewer suggests. This, and more, is done in Fig 1C, in the context of several datasets. Moreover – we do consider the extent of observed DE to represent one important component of the quality of the statistical approach. While the reviewer may not agree with this approach, changing it to include other comparison criteria would be beyond the scope of this manuscript.

That said, to alleviate several of the concerns raised by the reviewers we have performed an analysis on another integrated cohort (TCGA , Tahiri) where the method described in [22] (Percentile Normalization) is applied. This analysis also represents a comparison in a different set of cohorts, and therefore addresses the more general suggestion. We added the results of this analysis to the supplementary and address this analysis with a new paragraph in the discussion which reads:

“A potential line of inquiry to follow up on from this study is to compare AQuN results on other sets of cohorts and with other normalization approaches. We have applied a preliminary analysis on a second set of cohorts, consisting of the TCGA [59] and Tahiri [14] cohorts. We performed a differential expression analysis comparing cancer and control samples and include a 5^th^ normalization method previously referenced which is relevant in the context of case-vs-control experimental setups. Our results are presented in Supplementary Fig 4. When sorting miRNA according to their differential expression -log_10_(Q-value), we observe many known oncomiRs [60] are ranked higher and have more significant Q-values (Supplementary Table 1).”

We note that it may be difficult to see the line corresponding to Percentile Normalization [22] in the newly added Supplementary Fig 4 as it is overlaid by the set of Random (in dashed black lines).

3) Figure 2 is difficult to interpret and does not clarify how the impact of normalization on single miRNAs is evaluated.

**Response**: Indeed – we thank the reviewer for directing our attention to this. We have modified the caption of Fig 2 and the associated text to better describe its purpose. We do note that Figure 2 is intended for qualitative data visualization rather than impact quantification.

4) In the miRNA mimics assay, it would be worthy showing the transfection efficiency of hsa-mir-193b by qRT-PCR or other techniques.

**Response**: We have previously confirmed the transfection efficiency of individual miRNA mimics/pre-miRs by Taqman miRNA assays, which detect only mature miRNAs (Leivonen et al. Oncogene 2009). Transfection of mimics/pre-miRs resulted in marked overexpression of mature miRNAs, regardless of the levels of endogenous miRNAs. The data are shown below (Supplementary Figure S2 from Leivonen et al. Oncogene 2009).

*Leivonen et al. 2009, Supplementary Figure S2. Transfection of pre-miRs into MCF-7 cells results in overexpression of the mature miRNAs. MCF-7 cells were transfected with pre-miR constructs (20 nM) and incubated for 48 h. miRNA expression was determined from total cellular RNAs by qRT-PCR using Taqman microRNA assays, which detect mature miRNAs. Results are shown as relative expression normalized to RNU6B small nuclear RNA.*

To address this we have added the following sentence under Materials and methods (under the "Functional experiments"): "The transfection efficiency of miRNA mimics has been determined previously (Leivonen et al. 2009)."

Minor comments

5) A brief description of the AQuN method should be included in the results section, as it is an innovative method and its implementation is a core part of the work.

**Response:** As suggested, we have added a short paragraph on AQuN in the results section, referring the reader to the corresponding methods section for further details:

**“**AQuN is a novel variant of quantile normalization which utilizes quantization in its normalization process, thereby offering an added degree of control over noise that affects sample ranking and is evidently prevalent in miRNA datasets. Details of this method are available in the Methods section. Below we illustrate AQuN’s advantage over standard normalization methods in uncovering, otherwise nascent, signals in the joint dataset.”

We note that Reviewer #2, in fact, had a conflicting comment (minor comment #3). We are happy to further edit in co-ordination with the editor.

6) Figure 1 (top panel) and Fig 6 are difficult to read, it is not clear what the dashed lines that join the points correspond to. Volcano plots with dashed lines are not the ideal visualization to show an overall increase of statistical significance.

**Response:** We agree with the reviewer that this can be improved upon. Our goal was to provide a verbose view of the impact normalization has on significant results rather than provide a summarized view on the overall increase of statistical significance.
We have included the figures in vector art form in the supplementary and encourage readers to use them for an interactive interrogation of our results. We have also added the missing explanation of the dashed arrows to the caption as the reviewer suggests.

7) Table 1 reports data on the top four miRNAs identified by differential expression analysis. Results about all the 33 differentially expressed miRNAs can be report as supplementary material.

**Response:** We have added this list to the supplementary (extendedERtableSubset_33lst.xlsx), as suggested by the reviewer.

**Reviewer #2:**

Summary:
The authors developed a miRNA joint analysis approach based on applying adjusted quantile normalization (AQuN) across samples from various sources. In the merged breast cancer dataset using their approach, they discovered and validated that over-expression of has-miR-193b-5p was a new signature of the estrogen positive (ER) group. In short, this approach aims at leveraging statistical power of publicly available miRNA assays to identify novel biomarkers and preliminary therapeutic targets.

Major Comments:
The main concern of this approach development paper is that the authors haven’t done a comprehensive comparison between the outcomes of their proposed method and the original results in the studies they cited using the same dataset. And some results using their new approach is confusingly contradicted.

**Response**: We are not sure we understand this comment. There is no study that has analyzed these datasets in an integrated manner. In particular, we are not aware of any study that combined results between radically different platforms (in this case Agilent arrays and Exiqon assays). This is precisely the purpose of this work. We do address the concrete points below, including those related to literature studies. We hope that we thus also address this point.

Suggestions for improvements are listed below:
1. From the unnormalized and merged dataset in this paper, hsa-miR-190b does not look like a marker between ER+ and ER- group which is referred in Reference [30]. To evaluate the performance of proposed AQuN, the authors should have applied AQuN on the same dataset as that has been used in Reference [30]. Same for the has-miR-18a-5p.

**Response**: Indeed, when merging the datasets without normalizing, has-miR-190b does not present as substantially differentially expressed between ER+ and ER-, as the reviewer suggests. However, as shown, AQuN normalization recovers the differential expression signal of this previously known marker.

Unfortunately, AQuN cannot be applied as described to the dataset published in [30] as it is provided in summary form (number, median, min, max, cycle threshold) over samples by clinical type. We have attempted to reach out to the authors and request a raw version of their data and, unfortunately, have yet to hear back.

We note that regardless of the above conclusion, AQuN is designed to enable the merging of datasets coming from possibly very different measurement technologies and characteristics. It may also work better within the context of a nominally homogeneous data but this is not the strength we want to emphasize. Specifically – the discussion regarding statistical power is of essence here, underlining the advantage of merging datasets.

2. The authors identified four miRNAs with log2-fold-change above 10% and FDR less than 0.05 using AQuN on the merged dataset. However, only hsa-miR-193b-5p had indeed changed the viability of cells in the functional validation. The authors should clarify the following questions:

**Response**: We would call to attention that hsa-miR-193b-5p has the strongest effect, in our analysis, both in fold change and p-value.

a) The authors said they transduced miRNA mimics into MCF7 cells to validate hsa-miR-601, 424-3p, 936 and 193-5p. Considering three out of four of these miRNAs were identified as significantly downregulated in ER- compared to ER+, isn’t that a KO, instead of transduction, required for each of the three miRNAs?

**Response**: The purpose of the functional experiment was to study the impact of these miRNAs in breast cancer in general (not ER+ vs ER-). Figure 3B actually shows that miR-193b inhibits (by inducing apoptosis) also ER- KPL4 cells, so its tumor suppressive effect is not specific for ER+ only. We appreciate the reviewer´s question and acknowledge that this was written unclear. To address this issue, we have now rephrased the sentence introducing the functional data into: “To study any breast cancer functional significance of these top differentially expressed miRNAs we performed miRNA gain-of-function studies in the MCF-7 breast cancer cell line.”

b) How much hsa-miR-193b-5p was overexpressed in the MCF7 cells in the validation? Was it close to 10%, or much higher than that? If the latter, it might be explained as a cell burden to be toxic, instead of due to the real impact from this miRNA.

**Response**: We here repeat our answer to reviewer #1´s question number 4:

We have previously confirmed the transfection efficiency of individual miRNA mimics/pre-miRs by Taqman miRNA assays, which detect only mature miRNAs (Leivonen et al. Oncogene 2009). Transfection of mimics/pre-miRs resulted in marked overexpression of mature miRNAs, regardless of the levels of endogenous miRNAs. The data are shown below (Supplementary Figure S2 from Leivonen et al. Oncogene 2009).

We agree with the Reviewer that we should be cautious when interpreting the results from miRNA overexpression studies. However, when we overexpress a library of hundreds of miRNAs, only approximately 10% of the miRNAs in the library show a significant effect on cell viability. In addition, we always use scrambled/non-targeting miRNAs as controls. This suggests that in general, miRNA overexpression by transient transfection is not toxic for the cells.

*Leivonen et al. 2009, Supplementary Figure S2. Transfection of pre-miRs into MCF-7 cells results in overexpression of the mature miRNAs. MCF-7 cells were transfected with pre-miR constructs (20 nM) and incubated for 48 h. miRNA expression was determined from total cellular RNAs by qRT-PCR using Taqman microRNA assays, which detect mature miRNAs. Results are shown as relative expression normalized to RNU6B small nuclear RNA.*

c) In Reference [34], hsa-miR-601 was downregulated in breast cancer tissue compared to the control. The authors should have test if their method could confirm this using a case-vs-control setup, instead of ER-positive-vs-ER-negative. The reference is not a support of finding hsa-miR-601 differentially expressed between ER+ and ER-, making it hard to judge the robustness of AQuN. The for hsa-miR-936.

**Response**: The reviewer is correct in that this reference considers miRNA differential expression between breast cancer tissue vs. matched adjacent non-cancerous tissue. The reference was included as it does point to a potential role of this miRNA in breast cancer and thus supports hsa-miR-601 as an interesting candidate for breast cancer in general.

We performed an analysis using AQuN on a cohort of two datasets [TCGA, Tahiri] in a case-control setup to alleviate the reviewer’s concerns and found that miR-601 is under expressed in breast cancer vs normal breast at p<5E^-04^ and miR-936 at p<7^-05^.

|  | Fold Change | Differential Expression |
| --- | --- | --- |
| hsa-miR-601 | -0.71622 | 0.00048149 |
| hsa-miR-936 | -0.29304 | 6.54E-05 |

We also note that these two miRs were not very highly expressed in these two cohorts. Specifically, miR-601 has 1201/1278 samples with 0 expression for this miR, and miR-936 has 1194/1278 such samples.

d) how hsa-miR-936, a tumor-suppressor in ovarian cancer, indicates its role in breast cancer? And it was identified highly expressed in the ER- group by the author’s method again.

**Response**: There is clearly an association between the role of a gene in Cancer vs Normal and its potential role in ER + vs ER -. Evidently, this is not a one to one association, but you do expect many of the genes that are differentially expressed in ER to be differentially expressed in cancer. These properties are not orthogonal and therefore we view hsa-miR-936 involvement in cancer pathogenesis (moreover, in a related type of cancer) as support for our findings, even if not as a conclusive direct validation. We have clarified the text to reflect this distinction and thank the reviewer for pointing it out:

“While these findings do not directly validate our findings in ER differential expression, they support the potential association of these miRNA through related mechanisms of cancer pathogenesis”

3. If I understand correctly, there are totally 655 miRNAs to be studies in the merged dataset. Using MiTEA, the authors declared that no matching between mRNAs and these miRNAs was found when the dataset was unnormalized while 6 matchings between mRNAs and these miRNAs were found after AQuN. That only accounts for ~1% of total miRNA, which looks weird as many miRNAs have robust mRNA targets identified by both in silico and experimental methods. The authors should investigate such a poor matchup rate. Several suggestions below:

**Response**: The biological system investigated here is complex and noisy. A signal of decisive association in 1% is actually a good one. Certainly when comparing to non in the unnormalized data. We also note that the analysis presented in Figure 4 serves as further validation of the results for a single miRNA.

a) How were the mRNA and miRNA data matched? Is that each miRNA dataset has its own paired mRNA data? Or mRNA and miRNA are from totally different resources (e.g. different labs, different cells)? The author should have added these pieces of information in the method section to help the reader better understand their study setup.

**Response**: We thank the reviewer for pointing out this lack of clarity and have modified the text to simplify the details of our analysis:

“For the first analysis we investigated the impact of AQuN normalization on correlations between miRNA and the expression levels of their expected mRNA targets in the Oslo2 dataset.”

“To recapitulate, a matching occurs if the top of two lists of genes overlap to a high degree: the prominent predicted gene targets (by TargetScan) of miRNA $\mu$ and the list of genes ranked according to their sample-wise anti-correlation with their matched expression levels of miRNA $\mu$.”

Each cohort in our study has paired mRNA and miRNA data, i.e. these are collected for the same biological samples. In fact, this matched expression analysis is only possible for such matched data. For each miRNA we rank all mRNAs according to their sample-wise spearman correlation. This list is provided as input to miTEA (URL: <http://cbl-gorilla.cs.technion.ac.il/miTEA/> Paper: <https://academic.oup.com/nar/article/41/3/e45/2902370>) where more details can be found.

b) If the authors apply MiTEA on each dataset (DBCG, Oslo2, Micam and Stavanger) and their paired mRNA data independently, how many matchups could be found?

**Response**: We performed the above analysis, as requested, except for DBCG for which no clinical mapping information is available. Our findings are summarized in Supplementary Table 2.

c) What is the p-value and rank of hsa-miR-193b-5p in the MiTEA analysis?

**Response**: The miTEA webservice filters out miRNA that do not present with p-values that are less than a minimal significance threshold of 0.001. In this case, ranking genes according to their sample-wise-correlation with hsa-miR-193b-5p expression does not contain a significant enrichment of TargetScan miRNA targets at the top of the gene list, under the mHG null model (i.e. p>0.001).

d) The authors should apply other normalization methods on the merged dataset, implement the same sets of analysis in this section (refer to Figure 4 and 5), and compare their performances to that of AQuN.

**Response**: we thank the reviewer for this suggestion. It would indeed represent a more comprehensive study. We hope to perform such analysis, possibly on more and newer datasets, as a future direction.

4. In Figure 6, the authors claimed they observed “an overall increase of statistical confidence”. This seems to be misleading. What does “statistical confidence” refer to here? Does it refer to confidence interval or statistical power? The former one was set to 95% throughout the paper (or maybe 90% as the authors applied one-tailed test) and never changed. If it refers to the latter one, the increase of statistical power could be a directly effect from the increased sample size after merging, regardless of using AQuN or not, making this figure unnecessary. The authors should clarify on this.

**Response**: This is a great point that we did not explain sufficiently. The latter, is what we meant – We modified the caption text following the reviewer’s comment to be much more clear. From figure 1C we validate that this increase in power is not attainable from naively merging the datasets, and from other normalization methods.

Minor Comments:
1. The authors should have always used letters to label each panel of a figure. For example, use Figure 1A, 1B and 1C. But this is inconsistent throughout the paper. In many figures with complicated compositions of panels, they used Top, Middle and Bottom, which were hard for readers to keep on track.

**Response**: We thank the reviewer for alerting us on this possible confusion and have added panel labels as suggested.

2. When compared to Combat normalization, the authors used Tumor grade, subtype, ER, PR, HER2 and TP53 in addition to batch effects. However, it seems that many of these variables share co-linearity relationships. The authors should investigate this before applying normalization on all of them.

**Response**: We thank the reviewer for this comment, we have added previously omitted details, under “Differential expression reveals novel breast-cancer associated miRNA”, describing how we overcome co-linearity in the design matrix by applying the QR decomposition to select a linearly independent subset.

3. The current Result session is a mixture of methods and results. The authors should have separate them better in their corresponding sections.

**Response**: we agree with this comment. However – it is the nature of a methods paper to possibly present this type of mixture. We modified to further separate some methodological details, specifically moving technical details from the joint analysis with mRNA data sections.

4. In Figure 5, the authors should clarify what does P98 mean.

**Response**: We have improved the figure caption to read: “**Fig 5. GOrilla enrichment analysis comparison of hsa-miR-29b correlation with gene expression before and after miRNA normalization with AQuN.** Left) scatter of go term Q-values before and after AQuN. Red dots depicted with “$\geq$P98” are above the 98^th^ percentile of Normalized – Unnormalized Q-values (-log_10_) and green dots are for Unnormalized – Normalized. Right) List of GO terms in the $\geq$P98 group”

5. How was the performance of this normalization approach on the data derived from miRNA sequencing or Nanostring platform? Those are the two major platforms, if not more popular than microarrays, in the filed currently. Or is it suitable on TCGA dataset, which is a much larger cohort? The authors might want to either add a new analysis or comment it in the discussion.

**Response**: We refer the reviewer to our answer for comment 2C which includes an analysis on the TCGA cohort. We note that this cohort is not analyzed with the joint dataset described in the manuscript due to difficulties matching clinical labels. In the future we hope to report on miRNA sequencing and/or Nanostring results.

6. In the discussion, “The test evaluated in this analysis is Wilcoxon rank-sum as applied for our differential expression analysis in section 0”. What is “section 0”?

**Response**: We thank the reviewer and have corrected the faulty cross reference in the text.

7. The authors should clarify why miR-18a-5p and miR-29b-3p were chosen in the a-priori power analysis.

**Response**: miR-29b-3p was selected as it was the top result from our miTEA analysis (Table2, Fig4, Fig5). miR-18a-5p was selected for visual acuity following from Fig 2. We now include this information in the text.

**Have all data underlying the figures and results presented in the manuscript been provided?**
Large-scale datasets should be made available via a public repository as described in the *PLOS Computational Biology* [data availability policy](http://journals.plos.org/ploscompbiol/s/data-availability), and numerical data that underlies graphs or summary statistics should be provided in spreadsheet form as supporting information.

Reviewer #1: **No:** Table 1 reports data on the top four miRNAs identified by differential expression analysis. Results about all the 33 differentially expressed miRNAs can be report as supplementary material.

Reviewer #2: Yes

PLOS authors have the option to publish the peer review history of their article ([what does this mean?](https://journals.plos.org/ploscompbiol/s/editorial-and-peer-review-process#loc-peer-review-history)). If published, this will include your full peer review and any attached files.


If you choose “no”, your identity will remain anonymous but your review may still be made public.


**Do you want your identity to be public for this peer review?** For information about this choice, including consent withdrawal, please see our [Privacy Policy](https://www.plos.org/privacy-policy).

Reviewer #1: No

Reviewer #2: No
